# The interrelationship between calcium-phosphorus homeostasis and bone remodelling. Impact of dietary changes on bone in patients with primary hyperparathyroidism and chronic kidney disease

**DOI:** 10.3389/fbioe.2026.1800350

**Published:** 2026-06-23

**Authors:** Javier Martínez-Reina, José Luis Calvo-Gallego, Rocío Ruiz-Lozano, Peter Pivonka, Ralph Müller

**Affiliations:** 1 Departmento de Ingeniería Mecánica y Fabricación, Universidad de Sevilla, Seville, Spain; 2 Instituto de Biomedicina de Sevilla (IBIS). C/ Antonio Maura Montaner, Sevilla, Spain; 3 School of Mechanical, Medical and Process Engineering, Queensland University of Technology, Brisbane, QLD, Australia; 4 Institute for Biomechanics, ETH Zurich, Zurich, Switzerland

**Keywords:** bone cell population model, bone remodelling, calcium, chronic kidney disease, diet, homeostasis, hyperparathyroidism, phosphorus

## Abstract

**Introduction:**

Calcium–phosphorus homeostasis is closely linked to bone remodelling, and disturbances in either system contribute to bone loss in conditions such as chronic kidney disease and hyperparathyroidism. Existing physiological models, such as the Peterson and Riggs formulation, incorporate systemic Ca/P regulation but often rely on simplified representations of bone turnover. Consequently, current frameworks generally lack the detailed coupling between systemic mineral balance and the cellular mechanisms of bone remodelling required to evaluate how endocrine imbalance, renal dysfunction, or dietary changes jointly affect bone metabolism.

**Methods:**

In this work, we develop an integrated mathematical framework that overhauls the bone compartment of the Peterson and Riggs model by coupling it with a mechanistic cell-population model, while also introducing refined descriptions of systemic mineral fluxes. The model incorporates dynamic PTH regulation, mineral exchange between plasma, bone marrow, and bone matrix, and a revised mineralisation algorithm that links systemic mineral availability to local bone properties.

**Results:**

After calibration against clinical data for vitamin D deficiency, chronic kidney disease, and primary hyperparathyroidism, we use the model to explore how changes in dietary calcium, phosphate, and vitamin D intake influence Ca/P homeostasis and bone density under healthy and pathological conditions.

**Discussion:**

This integrated approach captures multiscale interactions between systemic mineral regulation and cellular bone remodelling, enabling quantitative analysis of disease mechanisms and the impact of dietary interventions on skeletal health.

## 1 Introduction

Calcium–phosphorus (Ca/P) homeostasis and bone remodelling are tightly interconnected physiological processes, and disturbances in either system contribute to significant skeletal deterioration in conditions such as chronic kidney disease (CKD) and hyperparathyroidism (HPT). Because bone serves simultaneously as a structural organ and a mineral reservoir, understanding how endocrine regulation, dietary inputs, and bone turnover interact is essential for explaining disease progression and for evaluating potential therapeutic or nutritional interventions.

Serum Ca is maintained within a narrow physiological range (from 2.1 to 2.6 mM) (Goldstein, 1990). Falling outside those limits can cause neurological and cardiac dysfunction (Shoback et al., 2016) and hence, its control must be very tight. The normal range of serum phosphate ( ${\text{PO}}_{4}$ ) is wider in relative terms, 0.9–1.5 mM (Wagner, 2024), and therefore its homeostasis is not as tightly regulated as Ca homeostasis. Probably, that is also the reason why the symptoms of hypo-hyperphosphatemia are usually only seen in moderate to severe disease states (Chang et al., 2014).

In order to maintain Ca/P homeostasis, the endocrine system performs fine control using three main mechanisms. Ca and P enter the system via the diet through intestinal absorption. Ca/P elimination from blood occurs mainly through renal excretion, and finally bone remodelling can retrieve Ca/P from the bone matrix where it is stored to put it back into the bloodstream. This process is orchestrated by the endocrine system through hormonal pathways. PTH and calcitonin orchestrate calcium homeostasis. In response to hypocalcemia, PTH stimulates bone resorption and renal Ca reabsorption while indirectly boosting intestinal absorption by upregulating calcitriol synthesis. Calcitriol, the active form of vitamin D, directly increases intestinal and renal Ca absorption and stabilizes the system by downregulating PTH secretion through a negative feedback loop.

Several modelling approaches have been developed to capture aspects of this complex system. Physiologically based Ca/P-regulation models, particularly the widely used framework by Peterson and Riggs (2010), describe hormonal control of mineral balance across the different compartments. i.e., the gut, kidney, plasma, and bone. However, these models include only a highly simplified representation of bone remodelling. Conversely, bone cell population models (BCPMs) provide a detailed mechanistic description of the cellular processes governing bone formation and resorption (Martínez-Reina and Pivonka, 2019; Martínez-Reina et al., 2021a; Martínez-Reina et al., 2021b; Martínez-Reina et al., 2022; Ruiz-Lozano et al., 2024), but typically assume constant PTH concentrations and do not incorporate systemic Ca/P homeostasis. As a result, existing models are limited in their ability to evaluate how endocrine dysregulation, renal impairment, or dietary changes jointly influence bone mineral density and bone quality over time.

What is missing is an integrated modelling framework that couples a mechanistic, cell-based bone remodelling model with a systemic Ca/P homeostasis model, allowing bidirectional interactions between endocrine regulation, mineral fluxes, and local bone turnover. Such a model is necessary to assess how CKD-related reductions in glomerular filtration, HPT-driven PTH overproduction, or alterations in calcium, phosphate, or vitamin D intake affect both serum biomarkers and bone structure. Current approaches cannot simultaneously capture these multiscale interactions.

In this work, we address this gap by developing a coupled model that integrates a detailed BCPM with the Ca/P-regulation system of Peterson and Riggs. We incorporate dynamic PTH regulation, mineral exchange between plasma, bone marrow, and bone matrix, and an improved mineralisation algorithm that links systemic mineral availability to tissue-level bone properties. After calibrating the model against clinical data for vitamin D deficiency, CKD, and HPT, we use it to analyse the influence of dietary calcium, phosphate, and vitamin D on Ca/P homeostasis and bone density under both healthy and pathological conditions. This integrated framework provides a quantitative tool for evaluating how systemic and local mechanisms jointly drive skeletal adaptation or deterioration.

## 2 Materials and methods

**FIGURE 1:**
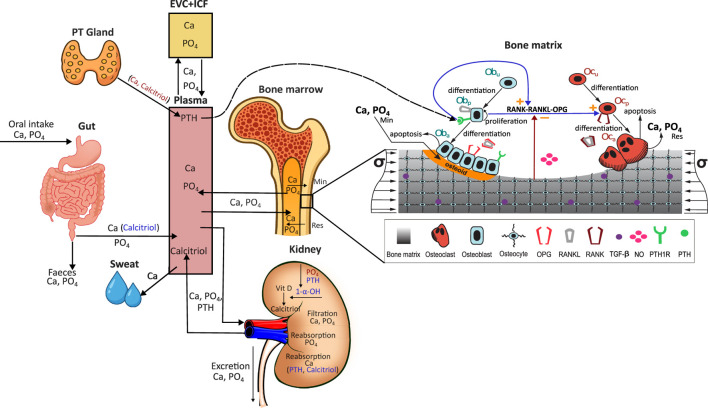
Schematic representation of the coupled model proposed in this work. Hormonal regulation of fluxes are indicated in parentheses: downregulating factors in red, upregulating factors in blue.

The model proposed in this work couples a BCPM with a systemic model of Ca/P homeostasis (Figure 1). The latter is an extension of the model developed by Peterson and Riggs (2010). These authors coupled Ca/P homeostasis with bone remodelling in a simplistic manner. For this reason, we have replaced the bone remodelling algorithm with our previously developed BCPM (Ruiz-Lozano et al., 2024). A brief summary of Peterson and Riggs’ model and the BCPM model are given in Sections 2.1 and 2.2, respectively. The link between both models is two-fold: (1) through the concentration of PTH in plasma, constant in the previous BCPM and now variable and obtained from the Ca/P homeostasis model; (2) through the exchange of minerals between the compartments, which ultimately governs bone matrix mineralisation. This required a revision of the mineralisation algorithm proposed in (Martínez-Reina et al., 2008), which is addressed in Section 2.3.

### 2.1 Ca/P homeostasis model

Peterson and Riggs (Peterson and Riggs, 2010) used the following sigmoid $E_{max}$ functions of a certain variable x:

$$H\left(x,\alpha ,\delta ,\gamma \right)=\frac{\alpha \cdot x^{\gamma }}{\delta^{\gamma }+x^{\gamma }}$$ (1a)

$$H^{-}\left(x,\rho ,\alpha ,\delta ,\gamma \right)=\alpha -\frac{\left(\alpha -\rho \right)\cdot x^{\gamma }}{\delta^{\gamma }+x^{\gamma }}$$ (1b)

$$H^{+}\left(x,\rho ,\alpha ,\delta ,\gamma \right)=\rho +\frac{\left(\alpha -\rho \right)\cdot x^{\gamma }}{\delta^{\gamma }+x^{\gamma }}$$ (1c)

where α and ρ are the maximum and minimum value of the sigmoid functions (being the minimum value equal to 0 in Equation 1a). δ represents the value of x at which the sigmoid function reaches the midpoint between α and ρ . The exponent γ controls the steepness of the transition between α and ρ . For simplicity, we will abbreviate the notation as follows:

$$H_{i,j}=H\left(A_{i},\alpha_{i,j},\delta_{i,j},\gamma_{i,j}\right)$$ (2a)

$$H_{i,j}^{-}=H^{-}\left(A_{i},\rho_{i,j},\alpha_{i,j},\delta_{i,j},\gamma_{i,j}\right)$$ (2b)

$$H_{i,j}^{+}=H^{+}\left(A_{i},\rho_{i,j},\alpha_{i,j},\delta_{i,j},\gamma_{i,j}\right)$$ (2c)

where $A_{i}$ represents the variable i of the systemic model (see Table 1) and the constants $\rho_{i,j}$ , $\alpha_{i,j}$ , $\delta_{i,j}$ and $\gamma_{i,j}$ are specific to the process in which $A_{i}$ affects the variation of $A_{j}$ . If the variable $A_{i}$ affects the flux from compartment $A_{j}$ to $A_{k}$ , the functions are termed $H_{i,j-k}$ , and the constants ρ , α , δ are termed accordingly. The fluxes from $A_{j}$ to $A_{k}$ are termed $J_{j-k}$ and are forced to be non-negative to prevent the reverse flow, $J_{k-j}$ , which could be governed by a different mechanism. The exceptions are $J_{1-4}$ and $J_{3-5}$ , as explained later.

**TABLE 1:** Homeostatic values of the variables of the model. The concentration pM stands for picomolar, i.e., pmol/L.

Compartment	Variable description	Name	Initial value	Units	References
Gut	Ca	${\text{A}}_{1}$	1	mmol	Adjusted
	Ca transporters	${\text{A}}_{2}$	0.5	fraction ∈[0,1]	Peterson and Riggs (2010)
	${\text{PO}}_{4}$	${\text{A}}_{3}$	0.839	mmol	Peterson and Riggs (2010)
Plasma	Ca	${\text{A}}_{4}$	7.35	mmol	Goldstein (1990)
	${\text{PO}}_{4}$	${\text{A}}_{5}$	3.6	mmol	Wagner (2024)
	Calcitriol	${\text{A}}_{6}$	300	pmol	Souberbielle et al. (2015)
	PTH	${\text{A}}_{7}$	10.2	pmol	Yalla et al. (2019)
EVC + ICF	Ca	${\text{A}}_{8}$	127	mmol	Adjusted from (Yeh et al., 2022; Evenepoel et al., 2024)
	${\text{PO}}_{4}$	${\text{A}}_{9}$	1966	mmol	Qadeer and Bashir (2025)
Kidney	1- α -hydroxylase	${\text{A}}_{10}$	30	pmol	Adjusted from (Peterson and Riggs, 2010)
PT gland	Active fraction PT glands	${\text{A}}_{11}$	0.5	fraction ∈[0,1]	Peterson and Riggs (2010)
	PT glands max. capacity	${\text{A}}_{12}$	1	-	Peterson and Riggs (2010)
Bone marrow	Ca	${\text{A}}_{13}$	3.65	mmol	Adjusted from (Hindorf et al., 2010; White et al., 1987; Gurkan and Akkus, 2007)
	${\text{PO}}_{4}$	${\text{A}}_{14}$	1.69	mmol	Stoichiometric with ${\text{A}}_{13}$
Bone matrix	Ca	${\text{A}}_{15}$	25,725	mmol	Adjusted from (Goldstein, 1990; Cashman, 2002)
	${\text{PO}}_{4}$	${\text{A}}_{16}$	11,936	mmol	Stoichiometric with ${\text{A}}_{15}$
Bone (BCPM)	Osteoblast precursors	$Ob_{p}$	2.386 $\cdot 10^{-3}$	pM	Adjusted from (Ruiz-Lozano et al., 2025)
	Active osteoblasts	$Ob_{a}$	3.448 $\cdot 10^{-4}$	pM	Adjusted from (Ruiz-Lozano et al., 2025)
	Osteoclast precursors	$Oc_{p}$	6.864 $\cdot 10^{-3}$	pM	Adjusted from (Ruiz-Lozano et al., 2025)
	Active osteoclasts	$Oc_{a}$	6.896 $\cdot 10^{-5}$	pM	Adjusted from (Ruiz-Lozano et al., 2025)
	Osteocytes	Ot	1.822 $\cdot 10^{-2}$	pM	Adjusted from (Ruiz-Lozano et al., 2025)

#### 2.1.1 Gut compartment

The Ca content in the gut is governed by the differential Equation 3, that considers the Ca intake $\left(D_{1}\right)$ , the flux of Ca to faeces $\left(J_{1-f}\right)$ and the flux to blood plasma $\left(J_{1-4}\right)$ .

$$\frac{dA_{1}}{dt}=\underset{\text{Ca intake}}{\underbrace{D_{1}}}-\underset{\text{Flux to feces}}{\underbrace{J_{1-f}}}-\underset{\text{Flux to plasma}}{\underbrace{J_{1-4}}}$$ (3)

The flux to faeces is proportional to the amount of Ca in the gut:

$$J_{1-f}=k_{1-f}\cdot A_{1}$$ (4)

where the flux rate $k_{1-f}$ is adjusted to produce $J_{1-f}=825$ mg Ca/day under homeostatic conditions and a daily intake $D_{1}=1000\;mg\;Ca/day=25\;mmol/day$ (Strewler, 2003). The flux of Ca into the blood plasma is split into passive and active absorption:

$$J_{1-4}=k_{1-4}\cdot \left(\underset{\text{Passive absorption}}{\underbrace{k_{pas}\cdot A_{1}-\varphi_{1-4}\cdot \frac{A_{4}}{A_{4}^{eq}}}}+\underset{\text{Active absorption}}{\underbrace{\frac{A_{2}}{A_{2}^{eq}}\cdot H_{1,1-4}}}\right)$$ (5)

The first two terms account for non-saturable paracellular (passive) absorption and the third term corresponds to the transcellular saturable absorption (active). This term accounts for the bioavailability of Ca transporters in intestinal enterocytes, i.e., the binding protein calbindin $D_{9k}$ (CaBP), through the factor $A_{2}/A_{2}^{eq}$ .^1^ More details about this relationship can be found in the Supplementary Material.

Sufficient evidence demonstrates a direct relationship between calcitriol levels and CaBP concentration (Fleet, 2022), and therefore with the variable ${\text{A}}_{2}\in \left[0,1\right]$ , whose variation is governed by Equation 6, as in (Peterson and Riggs, 2010):

$$\frac{dA_{2}}{dt}=\underset{\text{Activation of inactive absorbers}}{\underbrace{H_{6,2}^{+}\cdot \left(1-A_{2}\right)}}-\underset{\text{Deactivation of active absorbers}}{\underbrace{H_{6,2}^{-}\cdot A_{2}}}$$ (6)

where the functions $H_{6,2}^{+}$ and $H_{6,2}^{-}$ regulate, respectively, the activation of inactive Ca transporters and the deactivation of active transporters, both mediated by calcitriol levels, ${\text{A}}_{6}$ .

The PO_4_ content in the gut was modelled by Equation 7, similarly to that of Ca:

$$\frac{dA_{3}}{dt}=\underset{\text{Ca intake}}{\underbrace{D_{3}}}-\underset{\text{Flux to feces}}{\underbrace{J_{3-f}}}-\underset{\text{Flux to plasma}}{\underbrace{J_{3-5}}}$$ (7)

where $D_{3}=1400$ mg/day is the daily normal intake of phosphorus (Wagner, 2024). The flux to the faeces, $J_{3-f}$ , is defined by Equation 8, analogously to Equation 4:

$$J_{3-f}=k_{3-f}\cdot A_{3}$$ (8)

The flux to plasma $\left(J_{3-5}\right)$ was simplified in Equation 9 to account only for passive (paracellular) absorption, as in (Peterson and Riggs, 2010), but it was reformulated like the passive absorption in Equation 5, so to include the unidirectional backflux of ${\text{PO}}_{4}$ from blood to gut, thus following Wagner (2024):

$$J_{3-5}=k_{3-5}\cdot A_{3}-k_{5-3}\cdot A_{5}$$ (9)

The constants $k_{3-f}$ , $k_{3-5}$ and $k_{5-3}$ were adjusted so that in homeostatic conditions $J_{3-f}=500\;mg\;P/day$ , $J_{3-5}=1100\;mg\;P/day$ and $J_{5-3}=200\;mg\;P/day$ , respectively (Wagner, 2024).

#### 2.1.2 Ca and ${\text{PO}}_{4}$ in blood plasma (central compartment) and EVC + IVF compartment

Contrary to Peterson and Riggs (2010), we have distinguished blood plasma from the rest of the extracellular fluid, since the concentrations in both may be different due to Gibbs-Donnan equilibrium (Nguyen and Kurtz, 2006), and the values more easily found in the literature to validate our results are blood concentrations. The rest of the extracellular fluid, i.e., the extravascular compartment (EVC), and the intracellular fluid (ICF) will be assumed to act as a reservoir and will be grouped in the same compartment (EVC + ICF, see Figure 1). The Ca content in blood plasma is then governed by Equation 10:

$$\frac{dA_{4}}{dt}=\underset{\begin{array}{c} \text{Flux}\\ \text{from gut} \end{array}}{\underbrace{J_{1-4}}}+\underset{\begin{array}{c} \text{Flux from}\\ \text{bone marrow} \end{array}}{\underbrace{J_{13-4}}}+\underset{\begin{array}{c} \text{Flux from}\\ EVC+ICF \end{array}}{\underbrace{J_{8-4}}}-\underset{\begin{array}{c} \text{Flux to}\\ \text{bone marrow} \end{array}}{\underbrace{J_{4-13}}}-\underset{\begin{array}{c} \text{Flux to}\\ urine \end{array}}{\underbrace{J_{4-u}}}-\underset{\begin{array}{c} \text{Flux to}\\ sweat \end{array}}{\underbrace{J_{4-s}}}-\underset{\begin{array}{c} \text{Flux to}\\ EVC+ICF \end{array}}{\underbrace{J_{4-8}}}$$ (10)

The flux of Ca to urine was defined by Peterson and Riggs (2010) as in Equation 11:

$$J_{4-u}^{old}=\left(2-H_{6,4-u}\right)\cdot \left(\underset{\text{Filtration}}{\underbrace{k_{4-u}\cdot GFR\cdot \frac{A_{4}}{V_{\mathrm{plasma}}}}}-\underset{\text{Reabsorption}}{\underbrace{H_{4,4-u}\cdot H_{7,4-u}}}\right)$$ (11)

and we have reformulated it as in Equation 12:

$$J_{4-u}=H_{6,4-u}^{-}\cdot GFR\cdot \left(\underset{\text{Filtration}}{\underbrace{k_{4-u,\mathrm{fi}lt}\cdot \frac{A_{4}}{V_{\mathrm{plasma}}}}}-\underset{\text{Reabsorption}}{\underbrace{k_{4-u,reabs}\left(A_{4}\right)\cdot H_{7,4-u}^{+}}}\right)$$ (12)

It is worth commenting on the differences between the original and the current formulation. $H_{6,4-u}^{-}$ models the downregulation of Ca excretion by calcitriol and it must be noted that the factor $\left(2-H_{6,4-u}\right)$ (if $\alpha_{6,4-u}=2$ as used in (Peterson and Riggs, 2010)) is equivalent to $H_{6,4-u}^{-}$ with $\rho_{6,4-u}=0$ . Furthermore, the replacement of $H_{7,4-u}$ with $H_{7,4-u}^{+}$ is solely due to the choice of $\rho_{7,4-u}\neq 0$ . Following Parfitt et al. (1993), the expression (12) defines a “renal threshold”, corresponding to reabsorption, above which there is a linear increase in Ca excretion with increased plasma Ca concentration.^2^ This threshold depends on PTH through the function $H_{7,4-u}^{+}$ , so that reabsorption increases with PTH (Parfitt et al., 1993). The threshold also depends on ${\text{A}}_{4}$ through the function $k_{4-u,reabs}$ , which makes the reabsorbed Ca increase with ${\text{A}}_{4}$ and become saturated close to the homeostatic concentration $A_{4}^{eq}$ (Parfitt et al., 1993). The non-linear function $H_{4,4-u}$ , present in Peterson and Riggs’ formulation, was almost constant except for very low serum Ca levels. We have replaced it with the bilinear (saturated) function $k_{4-u,reabs}$ (Equation 13), which better approximates the effect of serum Ca on renal reabsorption as described by Parfitt et al. (1993):

$$k_{4-u,reabs}\left(A_{4}\right)=\left\{\begin{array}{cc} m_{4-u}\cdot A_{4}+n_{4-u} & \;if\;A_{4}<A_{4}^{eq}\\ m_{4-u}\cdot A_{4}^{eq}+n_{4-u} & \;if\;A_{4}\geq A_{4}^{eq} \end{array}\right.$$ (13)

where $m_{4-u}$ and $n_{4-u}$ are constants. It is important to enforce the condition $J_{4-u}\geq 0$ in this case.

The most relevant change in Equation 12 is the influence of the glomerular filtration rate (GFR). In contrast to Peterson and Riggs (2010), who assumed that only the amount of filtered Ca (first term in parentheses) was proportional to GFR, we now establish that this proportionality holds for both terms, since GFR controls the amount of liquid that enters the nephron and is subject to reabsorption. This change was made based on three premises:

1. To be in accordance with the excretion of ${\text{PO}}_{4}$ provided by Peterson and Riggs (2010) where GFR also multiplies both terms, as will be seen later.
2. Because blood is first filtrated in the glomerulus and then some constituents are passed into the tubules, where a fraction of them are reabsorbed and not excreted into urine. Hence, the amount of Ca reabsorbed in the proximal tubule and loop of Henle must be proportional to the filtrated amount, i.e., to GFR, in contrast to the original expression, where the reabsorbed amount was not proportional to GFR.
3. Chronic kidney disease (CKD) cannot be reliably modelled using the original expression. By doing so, CKD resulted in a declining filtration capacity (GFR), which yielded $J_{4-u}=0$ using the original model and when the threshold (reabsortion term) was roughly constant.^3^ This would inevitably lead to severe hypercalcaemia, in contradiction with clinical results. In contrast, Equation 12 leads to a decrease in $J_{4-u}$ with a decreasing GFR, though never reaching a null flux. A comparison of the two formulations in CKD is shown in the Supplementary Material.

We added the flux of Ca to sweat in Equation 10 (not considered in (Peterson and Riggs, 2010)), which was assumed proportional to the amount of Ca present in blood plasma, as described by Equation 14:

$$J_{4-s}=k_{4-s}\cdot A_{4}$$ (14)

where $k_{4-s}$ is the constant flux rate. The exchange of Ca between plasma and the EVC + IVF compartment is given by Equations 15, 16:

$$J_{4-8}=k_{4-8}\cdot A_{4}$$ (15)

$$J_{8-4}=k_{8-4}\cdot A_{8}$$ (16)

$A_{13}$ is the total amount of Ca contained in bone marrow. The exchange of Ca between bone marrow and blood plasma (J_4–13_ and J_13–4_) is governed by Equations 17, 18:

$$J_{4-13}=k_{4-13}\cdot A_{4}\cdot \frac{p}{p_{0}}$$ (17)

$$J_{13-4}=k_{13-4}\cdot A_{13}\cdot \frac{p_{0}}{p}$$ (18)

The exchange of a given species between compartments, such as $J_{4-8}$ and $J_{8-4}$ , should be proportional to the concentration of the exchanged species (Ca in that case) rather than to the amount of that species accumulated in each compartment. To do this, the distribution volume of each compartment (serum and EVC + IVF) must be taken into account, as they are not necessarily the same. However, in the fluxes defined thus far, the volume of each compartment can be indirectly considered in the rate constants, e.g., $k_{4-8}$ and $k_{8-4}$ , since those volumes can be assumed to remain constant. However, in the fluxes $J_{4-13}$ and $J_{13-4}$ , the volume of bone marrow is affected by changes in bone porosity. Hence, the correction factors $p_{0}/p$ and $p/p_{0}$ were introduced to consider the change in bone marrow volume (vascular porosity p) with respect to the homeostatic situation.

The ${\text{PO}}_{4}$ content in the blood plasma is governed by Equation 19:

$$\frac{dA_{5}}{dt}=\underset{\begin{array}{c} \text{Flux from}\\ \text{bone marrow} \end{array}}{\underbrace{J_{14-5}}}-\underset{\begin{array}{c} \text{Flux to}\\ \text{bone marrow} \end{array}}{\underbrace{J_{5-14}}}-\underset{\begin{array}{c} \text{Flux to}\\ urine \end{array}}{\underbrace{J_{5-u}}}+\underset{\begin{array}{c} Flux\\ \text{from gut} \end{array}}{\underbrace{J_{3-5}}}-\underset{\begin{array}{c} \text{Flux to}\\ EVC+ICF \end{array}}{\underbrace{J_{5-9}}}+\underset{\begin{array}{c} \text{Flux from}\\ EVC+ICF \end{array}}{\underbrace{J_{9-5}}}$$ (19)

The exchange of ${\text{PO}}_{4}$ between blood plasma and bone marrow (Equations 20, 21) is defined analogously to that of Ca.

$$J_{5-14}=k_{5-14}\cdot A_{5}\cdot \frac{p}{p_{0}}$$ (20)

$$J_{14-5}=k_{14-5}\cdot A_{14}\cdot \frac{p_{0}}{p}$$ (21)

The exchange between blood serum and EVC + ICF (Equations 22, 23) is also analogous to Ca exchange:

$$J_{5-9}=k_{5-9}\cdot A_{5}$$ (22)

$$J_{9-5}=k_{9-5}\cdot A_{9}$$ (23)

where the constants $k_{5-9}$ and $k_{9-5}$ were chosen to enforce $J_{5-9}=J_{9-5}$ in homeostasis and large enough to model the rapid osmotic equilibrium through capillaries (Hall, 2025), as previously stated. The flux to urine is defined by Equation 24, as in (Peterson and Riggs, 2010):

$$J_{5-u}=k_{5-u}\cdot GFR\cdot \left(\underset{\text{Filtration}}{\underbrace{\frac{A_{5}}{V_{\mathrm{plasma}}}}}-\underset{\text{Reabsortion}}{\underbrace{\varphi_{5-u}}}\right)$$ (24)

where $\varphi_{5-u}$ is a constant threshold. Of note, we have not included the flux of ${\text{PO}}_{4}$ to sweat, as it is scarcely eliminated via this pathway (Shirreffs and Maughan, 1985).

The concentrations of Ca and ${\text{PO}}_{4}$ in the EVC + IVF compartment are governed by the Equations 25, 26:

$$\frac{dA_{8}}{dt}=J_{4-8}-J_{8-4}$$ (25)

$$\frac{dA_{9}}{dt}=J_{5-9}-J_{9-5}$$ (26)

where the fluxes were given in Equations 15, 16, 22 and 23.

#### 2.1.3 Endocrine factors

Ca/P homeostasis is regulated by a number of hormones among which only PTH and vitamin D are considered in this work. Calcitriol $\left(1,25-{\left(OH\right)}_{2}D\right)$ , the active form of vitamin D, regulates Ca intestinal absorption and renal filtration, but also plays a role in PTH production, through the activation and growth of the PT glands. The content of calcitriol in blood plasma is governed by Equation 27, which is analogous to that proposed by Peterson and Riggs (2010):

$$\frac{dA_{6}}{dt}=\underset{\text{Production in the kidney}}{\underbrace{k_{25-OH,D}\cdot A_{10}}}-\underset{\text{Degradation}}{\underbrace{k_{6D}\cdot A_{6}}}$$ (27)

where $k_{6D}=0.1\;h^{-1}$ is the degradation rate of calcitriol, whose half-life in blood serum is between 5 and 10 h (Bailie and Johnson, 2002). The first term models the conversion of calcidiol into calcitriol in the kidney through the enzymatic action of 1- α -hydroxylase ( ${\text{A}}_{10}$ ). We have introduced the conversion factor $k_{25-OH,D}$ to account for variations in calcidiol (or equivalently, vitamin D) levels, in contrast to Peterson and Riggs (2010) who assumed $k_{25-OH,D}=1$ , thus implying that calcitriol production equals the concentration of 1- α -hydroxylase, which does not allow to investigate aspects of vitamin D deficiency and supplementation, as done in this work. For homeostasis, we assumed $k_{25-OH,D}=1$ and calculated $A_{10}^{eq}$ to enforce equilibrium, i.e., $dA_{6}/dt=0$ .

PTH in plasma is governed by Equation 28, which takes into account the activity of the PT glands through the variables ${\text{A}}_{11}$ and ${\text{A}}_{12}$ .

$$\frac{dA_{7}}{dt}=\underset{\text{PTH production by PT glands}}{\underbrace{k_{7S}\cdot \frac{A_{11}}{A_{11}^{eq}}\cdot A_{12}}}-\underset{\text{Degradation}}{\underbrace{k_{7D}\cdot A_{7}}}$$ (28)

where $k_{7D}=10\;h^{-1}$ is the degradation rate of PTH, whose half-life in blood serum is around 4 min (Bieglmayer et al., 2002). $k_{7S}$ is a production rate constant that replaces the sigmoid function of serum Ca used by Peterson and Riggs (2010), which was relatively constant. This change is justified by the fact that the dependence of serum Ca on PTH production is retained through its influence on ${\text{A}}_{11}$ , as reflected in Equation 30. Note that $A_{11}$ was also normalised to the initial and homeostatic value $\left(A_{11}^{eq}=0.5\right)$ as done in (Peterson and Riggs, 2010). $k_{7S}=102\;pmol/h$ was adjusted to enforce homeostatic equilibrium, i.e., $A_{7}=A_{7}^{eq}=10.2\;pmol$ when $A_{11}=A_{11}^{eq}=0.5$ and $A_{12}=A_{12}^{eq}=1$ .

1- α -hydroxylase is produced in the kidney mediated via PTH and inhibited by ${\text{PO}}_{4}$ plasma levels (Parfitt et al., 1993). These effects were modelled in (Peterson and Riggs, 2010) respectively through the functions $H_{7,10}$ and $H_{5,10}^{-}$ , in Equation 29.

$$\frac{dA_{10}}{dt}=\underset{Productionof1-\alpha -hydroxylase}{\underbrace{k_{10S}\cdot H_{7,10}^{+}\cdot H_{5,10}^{-}}}-\underset{\text{Degradation}}{\underbrace{k_{10D}\cdot A_{10}}}$$ (29)

where $k_{10S}$ and $k_{10D}$ are the production and degradation rates, respectively.

The variable ${\text{A}}_{11}$ was termed “PT pool” in (Peterson and Riggs, 2010) and measures the fraction of the PT gland that is active, i.e., PT chief cells, responsible for producing PTH. Its evolution is described by Equation 30:

$$\frac{dA_{11}}{dt}=\underset{\text{Activation of inactive chief cells}}{\underbrace{\left(1-A_{11}\right)\cdot \alpha_{11}\cdot \left(0.85\cdot T_{6,4}^{-}+0.15\right)}}-\underset{\text{Deactivation of active chief cells}}{\underbrace{A_{11}\cdot \alpha_{11}\cdot \left(0.85\cdot T_{6,4}^{+}+0.15\right)}}$$ (30)

where $\alpha_{11}$ is a constant and the regulatory sigmoid functions $T_{6,4}^{\pm }$ quantify the effect of serum Ca and calcitriol levels on PT chief cells, which will be activated if Ca or calcitriol are below normal and deactivated if they are above normal. These functions are given by Equation 31.

$$T_{6,4}^{\pm }=1\pm tanh\left\{b_{T6,4}\;\left[\frac{A_{6}}{V_{\mathrm{plasma}}}-\delta_{T_{6,4}}\cdot {\left(\frac{A_{4}^{eq}}{A_{4}}\right)}^{\gamma_{4,11}}\right]\right\}$$ (31)

where $b_{T6,4}$ , $\delta_{T_{6,4}}$ and $\gamma_{4,11}$ are constants. The activity of PT chief cells, $A_{11}$ , could be affected by temporary changes in calcitriol and Ca serum levels. However, the amount of secreted PTH is also dependent on the PT gland size $\left(A_{12}\right)$ , which is related to the proliferation of PT chief cells and responsible for long-term abnormal production of PTH in pHPT. Its variation is modelled by Equation 32.

$$\frac{dA_{12}}{dt}=\underset{\text{Growth}}{\underbrace{k_{12S}\cdot H_{6,12}^{-}}}-\underset{\text{Reduction}}{\underbrace{k_{12D}\cdot A_{12}}}+\underset{\text{pHPT}}{\underbrace{k_{HPT}}}$$ (32)

where $k_{12S}$ and $k_{12D}$ are the normal production and degradation rates, but are assumed equal, as in (Peterson and Riggs, 2010). The growth rate is modulated by the function $H_{6,12}^{-}$ , which models secondary HPT (sHPT) produced by vitamin D deficit, which otherwise cannot be simulated as the influence of calcitriol on Equation 30 through $\delta_{T_{6,4}}$ is insufficient to account for it. The constant $k_{HPT}$ was added in this work to model primary HPT (pHPT). If $k_{HPT}=0$ , the homeostatic equilibrium of Equation 32 is enforced by ensuring that $H_{6,12}^{-}\left(A_{6}^{eq}\right)=A_{12}^{eq}=1$ .

#### 2.1.4 Exchange of Ca and ${\text{PO}}_{4}$ between plasma, bone marrow and bone matrix

The current formulation of Ca/P exchange between plasma, bone marrow and bone matrix is substantially different to the model presented by Peterson and Riggs (2010). These authors defined the intracellular compartment (equivalent to our EVC + IVF) as an intermediate step between blood plasma and bone matrix. We have put the EVC + IVF in parallel to blood plasma and defined a new compartment, bone marrow, to act as the intermediate step between plasma and bone matrix (see Figure 1). After redefining these compartments, the fluxes implemented here follow the same structure as in (Peterson and Riggs, 2010), but with different meanings and expressions. Thus, the fluxes between ${\text{A}}_{4}$ and ${\text{A}}_{13}$ (for Ca) and between ${\text{A}}_{5}$ and ${\text{A}}_{14}$ (for ${\text{PO}}_{4}$ ) represent the rapid or immediate exchange of minerals between blood plasma and bone marrow. Meanwhile, the fluxes from ${\text{A}}_{13}$ to ${\text{A}}_{15}$ (for Ca) and from ${\text{A}}_{14}$ to ${\text{A}}_{16}$ (for ${\text{PO}}_{4}$ ) represent the slower deposition of minerals present in bone marrow into bone matrix due to the mineralisation process. The reverse fluxes, from ${\text{A}}_{15}$ to ${\text{A}}_{13}$ and from ${\text{A}}_{16}$ to ${\text{A}}_{14}$ , represent the release of minerals from bone matrix due to bone resorption. The Ca balance equations for the bone marrow and bone matrix compartments are given by Equations 33, 34.

$$\frac{dA_{13}}{dt}=\underset{\text{Flux from plasma}}{\underbrace{J_{4-13}}}-\underset{\text{Flux to plasma}}{\underbrace{J_{13-4}}}+\underset{\text{Flux from bone matrix}}{\underbrace{J_{15-13}}}-\underset{\text{Flux to bone matrix}}{\underbrace{J_{13-15}}}$$ (33)

$$\frac{dA_{15}}{dt}=\underset{\text{Flux from bone marrow}}{\underbrace{J_{13-15}}}-\underset{\text{Flux to bone marrow}}{\underbrace{J_{15-13}}}$$ (34)

The corresponding balance equations for PO_4_ are given by Equations 35, 36.

$$\frac{dA_{14}}{dt}=\underset{\text{Flux from plasma}}{\underbrace{J_{5-14}}}-\underset{\text{Flux to plasma}}{\underbrace{J_{14-5}}}+\underset{\text{Flux from bone matrix}}{\underbrace{J_{16-14}}}-\underset{\text{Flux to bone matrix}}{\underbrace{J_{14-16}}}$$ (35)

$$\frac{dA_{16}}{dt}=\underset{\text{Flux from bone marrow}}{\underbrace{J_{14-16}}}-\underset{\text{Flux to bone marrow}}{\underbrace{J_{16-14}}}$$ (36)

These fluxes are described in more detail in Section 2.4, where the effects of mineralisation and bone resorption on the exchange of minerals are addressed.

### 2.2 Bone cell population model

In this section, we briefly summarise the most relevant aspects of the previously developed BCPM (Ruiz-Lozano et al., 2024) that replaces the bone compartment of Peterson and Riggs’ model, with particular emphasis on those aspects affected by PTH, which is the main link between both models. The reader is referred to the Supplementary Material for a more detailed description.

The BCPM considers the interactions between bone cells and takes into account catabolic (RANK–RANKL–OPG) and anabolic (Wnt–Sclerostin (Scl)–LRP5/6) signalling pathways, together with the action of PTH, nitric oxide (NO), transforming growth factor β (TGF- β ) and mechanobiological feedback. Bone mineralisation is taken into account by adapting the algorithm developed previously (Martínez-Reina and Pivonka, 2019; Martínez-Reina et al., 2008). Accumulation and repair of microstructural damage is modelled as in Martínez-Reina et al. (2021b). Bone cell concentrations are the state variables of the model: osteoblast precursor cells ( ${\text{Ob}}_{\text{p}}$ ), active osteoblasts ( ${\text{Ob}}_{\text{a}}$ ), osteoclast precursor cells ( ${\text{Oc}}_{\text{p}}$ ), active osteoclasts ( ${\text{Oc}}_{\text{a}}$ ) and osteocytes (Ot), while the cell pools of uncommitted progenitor cells of both lineages ( ${\text{Ob}}_{\text{u}}$ , ${\text{Oc}}_{\text{u}}$ ) were assumed constant. Equations 37–41 constitute the set of differential equations that govern cell populations:

$$\begin{array}{l} \begin{array}{cc} \frac{d\;Ob_{p}}{dt}= & D_{Ob_{u}}\cdot Ob_{u}\cdot \pi_{act,Ob_{u}}^{TGF-\beta }-D_{Ob_{p}}\cdot Ob_{p}\cdot \pi_{rep,Ob_{p}}^{TGF-\beta }\\ +P_{Ob_{p}}\cdot Ob_{p}\cdot \pi_{act,Ob_{p}}^{Wnt} \end{array} \end{array}$$ (37)

$$\frac{d\;Ob_{a}}{dt}=D_{Ob_{p}}\cdot Ob_{p}\cdot \pi_{rep,Ob_{p}}^{TGF-\beta }-\Delta_{Ob_{a}}\cdot Ob_{a}$$ (38)

$$\frac{d\;Oc_{p}}{dt}=D_{Oc_{u}}\cdot Oc_{u}\cdot \pi_{act,Oc_{u}}^{RANK}-D_{Oc_{p}}\cdot Oc_{p}\cdot \pi_{act,Oc_{p}}^{RANK}$$ (39)

$$\frac{d\;Oc_{a}}{dt}=D_{Oc_{p}}\cdot Oc_{p}\cdot \pi_{act,Oc_{p}}^{RANK}-A_{Oc_{a}}\cdot Oc_{a}\cdot \pi_{act,Oc_{a}}^{TGF-\beta }$$ (40)

$$\frac{d\;Ot}{dt}=\eta \;\frac{d\;f_{bm}}{dt}$$ (41)

where $D_{Ob_{u}}$ , $D_{Ob_{p}}$ , $D_{Oc_{u}}$ and $D_{Oc_{p}}$ are the differentiation rates of ${\text{Ob}}_{\text{u}}$ , ${\text{Ob}}_{\text{p}}$ , ${\text{Oc}}_{\text{u}}$ and ${\text{Oc}}_{\text{p}}$ , respectively; $A_{Oc_{a}}$ is the apoptosis rate of ${\text{Oc}}_{\text{a}}$ and $\Delta_{Ob_{a}}$ is the rate of clearance of active osteoblasts through apoptosis or differentiation into osteocytes. $P_{Ob_{p}}$ is the proliferation rate of ${\text{Ob}}_{\text{p}}$ , a process that is mediated by the Wnt signalling pathway through the activator function $\pi_{act,Ob_{p}}^{Wnt}$ . $\pi_{act,Ob_{u}}^{TGF-\beta }$ , $\pi_{rep,Ob_{p}}^{TGF-\beta }$ and $\pi_{act,Oc_{p}}^{TGF-\beta }$ represent activator and repressor functions related to the binding of TGF- β to its receptor. Similarly, $\pi_{act,Oc_{u}}^{RANK}$ and $\pi_{act,Oc_{p}}^{RANK}$ are the activator functions related to RANK-RANKL binding, which govern osteoclasts differentiation and depend on the concentration of RANKL. This concentration increases with serum PTH levels ( ${\text{A}}_{7}$ ), which thus enhances bone turnover. This is the main change introduced in our BCPM, as PTH concentration was assumed constant in our previous versions of the BCPM. For brevity, the BCPM is explained in full detail in the Supplementary Material. A schematic representation of the BCPM was shown on the right-hand side of Figure 1.

Bone volume fraction, fbm, is defined (Equation 42) as the volume of bone matrix $\left(V_{b}\right)$ per unit volume of the representative element (RVE):

$$f_{bm}=\frac{V_{b}}{V_{\mathrm{RVE}}}=1-p$$ (42)

where p ∈[0,1] is the vascular porosity. Equation 41 implies that the population of osteocytes varies proportionally to $f_{bm}$ , thus assuming that the density of osteocytes, η , is constant within the bone matrix, as done in (Martin et al., 2019). Finally, the variation of fbm is obtained through the balance between resorbed and formed tissue described by Equation 43.

$$\frac{df_{bm}}{dt}=-k_{res}\cdot Oc_{a}+k_{form}\cdot Ob_{a}$$ (43)

where $k_{res}$ and $k_{form}$ are, respectively, the bone resorption and formation rates.

A mechanistic approach is needed to consider the effect of loading on bone density. This is not possible with the original BCPM developed by Lemaire et al. (2004) and implemented by Peterson and Riggs in their model, for two reasons: (1) its phenomenological equations are not mechanistic in nature and do not account for the stress-strain state; (2) the model was formulated on the whole skeletal scale, without considering the specific mechanical stimulus at the RVE under study.

Our BCPM overcomes both limitations. On the one hand, it considers the mechanical stimulus in the RVE on which osteoclast differentiation and osteoblast precursor proliferation depend (see the Supplementary Material for a detailed description of mechanical feedback). In addition, porosity and composition of bone matrix determine bone stiffness (see Section 2.3).

On the other hand, we formulate a mapping procedure to scale the RVE level (local) up to the skeleton level (systemic) in order to derive the fluxes between compartments, which are systemic, while keeping the estimation of mechanical stimulus at the local level. This mapping procedure is explained in the Supplementary Material and results in the application of our model to an RVE with $f_{bm}=44\%$ , representative of the average porosity of the entire skeleton.

### 2.3 Mineralisation of bone matrix. Composition and stiffness

As discussed above, bone stiffness must be considered in a mechanistic model such as the present one. This stiffness depends on bone porosity (calculated using Equation 43) and on the composition of bone matrix, derived from the mineral content and calculated as explained in this section. In addition, the process of bone matrix mineralisation is key to regulate Ca/P homeostasis and completes the formulation of fluxes between the compartments, although it was not considered by Peterson and Riggs (2010).

Bone is often treated as a composite of tissue and pores. Thus, a representative volume element $V_{RVE}$ can be divided into the volume of bone matrix, $V_{b}$ , and the volume of vascular pores, $V_{vas}$ . Bone matrix is composed of an inorganic phase (mineral), an organic phase (mainly collagen) and water (Equation 44). The volume of each phase is designated as $V_{m}$ , $V_{o}$ and $V_{w}$ , respectively:

$$V_{RVE}=V_{b}+V_{vas}=V_{m}+V_{o}+V_{w}+V_{vas}$$ (44)

We define the specific volumes $v_{o}=V_{o}/V_{b}$ , $v_{m}=V_{m}/V_{b}$ and $v_{w}=V_{w}/V_{b}$ , such that $v_{o}+v_{w}+v_{m}=1$ holds. Osteoid, the tissue laid *de novo* by osteblasts, only contains water and organic phase. During the mineralisation process, hydroxyapatite accumulates by displacing water (Hernández et al., 2000), so that $v_{o}$ would remain constant, i.e., $v_{o}=0.36$ (Martin, 1984). Therefore, the mineral content can be measured by $v_{m}$ and provides the tissue density through Equation 45.

$$\rho_{t}=\frac{m_{b}}{V_{b}}=\frac{m_{m}+m_{o}+m_{w}}{V_{b}}=\rho_{m}\;v_{m}+\rho_{o}\;v_{o}+\rho_{w}\;v_{w}$$ (45)

where $m_{b}$ is the mass of bone matrix, while $m_{i}$ and $\rho_{i}$ (with i = m,o,w) are the masses and densities of the three phases, mineral, organic and water, being $\rho_{w}=1.0\;g/cm^{3}\;\rho_{m}=3.0\;g/cm^{3}$ and $\rho_{o}=1.4\;g/cm^{3}$ (Martin, 1984). The apparent density is given by Equation 46:

$$\rho =\frac{m_{b}}{V_{\mathrm{RVE}}}=\frac{m_{b}}{V_{b}}\cdot \frac{V_{b}}{V_{\mathrm{RVE}}}=\rho_{t}\cdot f_{bm}$$ (46)

Bone was assumed to be an isotropic material with a Young’s modulus given by the empirical relationship (Equation 47) obtained by Morgan et al. (2003):

$$E\left(MPa\right)=6850\;\rho^{1.49}$$ (47)

### 2.4 Algorithm of bone mineralisation

In previous works, we have assumed that mineral content increases with time following a certain mineralisation law, $v_{m}\left(t\right)$ . Although complex laws have been proposed, the process can be simplified by assuming a decreasing exponential law (Ruffoni et al., 2007):

$$v_{m}\left(t\right)=v_{m}^{max}\;\left(1-e^{-\kappa \;t}\right)$$ (48)

where κ is a parameter that measures the rate of mineral deposition and $v_{m}^{max}$ is the maximum mineral content, which can be associated with the saturation of bone matrix. In fact, Equation 48 is the solution of the first-order differential Equation 49, with saturation controlled by $v_{m}^{max}$ :

$${\dot{v}}_{m}=\kappa \;\left(v_{m}^{\max}-v_{m}\right)=\kappa \;v_{m}^{\max}\left(1-\frac{v_{m}}{v_{m}^{\max}}\right)$$ (49)

The temporal law $v_{m}\left(t\right)$ of Equation 48, or an analogous one, has been used in previous works, where we implemented a queue algorithm that takes into account the tissue patches that have been formed in the recent history and partially resorbed by osteoclasts. The age of each patch is univocally related to its mineral content by the law $v_{m}\left(t\right)$ , and the mineral content of the RVE is obtained by summing the contents of each patch, i.e., of each element of the queue.

**FIGURE 2:**
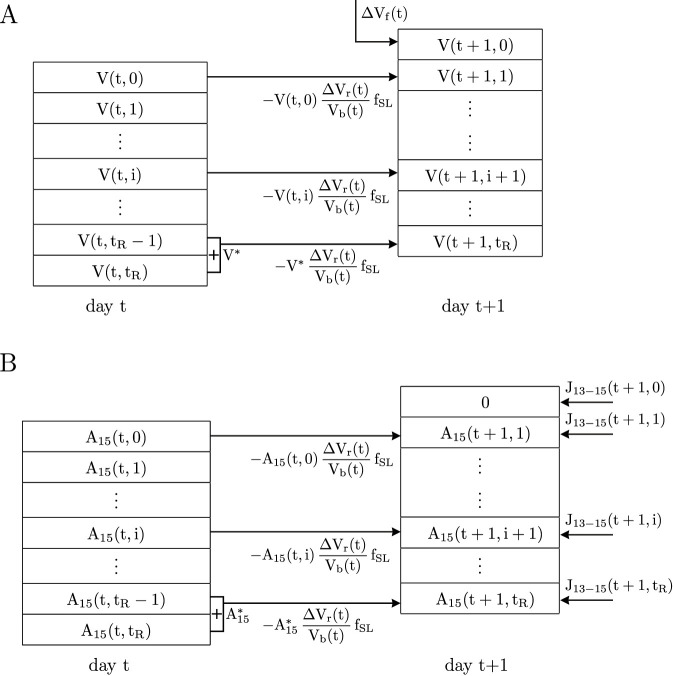
**(A)** Queue of tissue volume. **(B)** Queue of Ca content.

Here we have adapted the queue algorithm proposed in (Martínez-Reina et al., 2008) to account for a new approach to model the mineralisation of bone matrix. We have distinguished two queues. In the queue of Figure 2A, termed “tissue volume queue”, V(t,i) designates the bone tissue volume formed i days ago and still present at day t. Note that the subscript b has been omitted to differentiate this volume from the total volume, i.e., $V_{b}\left(t\right)=\sum_{i=1}^{t_{R}}V\left(t,i\right)$ .

The parameter $t_{R}$ was called residence time in (Martínez-Reina et al., 2008), but it can also be interpreted as a truncation time needed to reduce the computation cost of the algorithm. Thus, $V\left(t,t_{R}\right)$ contains the amount of tissue older than $t_{R}$ , which was assumed in (Martínez-Reina et al., 2008) to be completely mineralised, i.e., having $v_{m}=v_{m}^{max}$ . In (Martínez-Reina et al., 2008), we assumed that the mineral content of each element was determined by its age through the mineralisation law Equation 48. Here, we have defined a different queue for the Ca content, termed “calcium queue”, which allocates $A_{15}\left(t,i\right)$ , the moles of Ca stored in the corresponding element of the “tissue volume queue”, V(t,i) (see Figure 2B). Each day, the queues are updated as follows:

1. The tissue formed today is assigned in Equation 50 to the first element of the “tissue volume queue”:

$$V\left(t+1,0\right)=\Delta V_{f}\left(t\right)=Ob_{a}\;k_{form}$$ (50)

but this element contains no mineral, i.e., $V_{m}\left(t+1,0\right)=0$ .

2. The indices t and i of the rest of elements are increased and bone resorption is taken into account by removing the amount of tissue $\Delta V_{r}\left(t\right)=k_{res}\;Oc_{a}\;\Delta t$ (recall Equation 43), which is taken proportionally from the elements of the queue (Equation 51) depending on the volume each element occupies in the total:

$$V\left(t+1,i+1\right)=V\left(t,i\right)-V\left(t,i\right)\;\frac{\Delta V_{r}\left(t\right)}{V_{b}\left(t\right)}\;f_{SL}$$ (51)

where $f_{SL}$ is a factor used to scale up the model of local bone remodelling, since $\Delta V_{r}\left(t\right)$ is calculated at the RVE but it must be representative of what occurs at the systemic level, i.e., in the entire skeleton. See the Supplementary Material for more details. The amount of Ca that each element contains is removed accordingly (see Equation 52), i.e., proportionally to the amount of Ca stored in each element.

3. Mineralisation of each element is accounted for through $J_{13-15}$ , the flux of mineral from bone marrow, which is provided later in Equation 55

$$A_{15}\left(t+1,i+1\right)=A_{15}\left(t,i\right)-A_{15}\left(t,i\right)\;\frac{\Delta V_{r}\left(t\right)}{V_{b}\left(t\right)}\;f_{SL}+J_{13-15}\left(t+1,i\right)$$ (52)

4. Finally, the mineral content of each element is calculated through Equation 53:

$$v_{m}\left(t+1,i+1\right)=\frac{V_{m}\left(t+1,i+1\right)}{V\left(t+1,i+1\right)}=\frac{A_{15}\left(t+1,i+1\right)}{V\left(t+1,i+1\right)}\cdot \frac{M_{HA}}{\rho_{m}\cdot n_{Ca,HA}}$$ (53)

where $A_{15}\left(t+1,i+1\right)$ is the number of moles of Ca contained in the element V(t+1,i+1) , and $M_{HA}=1004.62$ g/mol and $n_{Ca,HA}=10$ are the molar mass and the number of Ca atoms of pure hydroxyapatite.

Since we have assumed that pure hydroxyapatite is the only component of the mineral phase, the number of moles of ${\text{PO}}_{4}$ contained in bone matrix, ${\text{A}}_{16}$ , is given in Equation 54 by the stoichiometric relationship:

$$A_{16}\left(t,i\right)=0.464\cdot A_{15}\left(t,i\right)$$ (54)

Fluxes $J_{13-15}$ and $J_{14-16}$ are determined by the mineralisation process. Following (Poorhemati and Komarova, 2024), we have assumed that mineral deposition is regulated by the availability of ${\text{PO}}_{4}$ and Ca ions within bone marrow. Specifically, the limiting factor is the compound that is stoichiometrically less abundant. Thus, if $A_{14}>0.464\cdot A_{13}$ , mineralisation is limited by the amount of Ca in bone marrow, ${\text{A}}_{13}$ , and then:

$$J_{13-15}\left(t,i\right)=k_{13-15}\cdot A_{13}\left(t\right)\cdot \frac{p_{0}}{p\left(t\right)}\cdot \frac{V\left(t,i\right)}{V_{b}\left(t\right)}\cdot \left(1-\frac{v_{m}\left(t,i\right)}{v_{m}^{\max}}\right)$$ (55)

while the amount of ${\text{PO}}_{4}$ eposited in the bone matrix is obtained in Equation 56 from the stoichiometric ratio:

$$J_{14-16}\left(t,i\right)=0.464\cdot J_{13-15}\left(t,i\right)$$ (56)

In contrast, if $A_{14}<0.464\cdot A_{13}$ , mineralisation is limited by the amount of PO4 in bone marrow, ${\text{A}}_{14}$ , and then assessed through Equations 57, 58:

$$J_{14-16}\left(t,i\right)=k_{13-15}\cdot A_{14}\left(t\right)\cdot \frac{p_{0}}{p\left(t\right)}\cdot \frac{V\left(t,i\right)}{V_{b}\left(t\right)}\cdot \left(1-\frac{v_{m}\left(t,i\right)}{v_{m}^{\max}}\right)$$ (57)

$$J_{13-15}\left(t,i\right)=\frac{J_{14-16}\left(t,i\right)}{0.464}$$ (58)

where the constant $k_{13-15}$ controls the mineralisation rate in both cases, along with the concentration of the limiting compound: either Ca ( ${\text{A}}_{13}$ ) or ${\text{PO}}_{4}$ ( ${\text{A}}_{14}$ ). It should be noted that ${\text{A}}_{13}$ and ${\text{A}}_{14}$ represent the amounts of Ca and ${\text{PO}}_{4}$ in bone marrow; however, in order for the fluxes to be governed by the concentrations, those amounts must be divided by the volume of bone marrow, i.e., the volume of pores or porosity. In fact, we assume that the relative variations of porosity in the RVE can be scaled up to the whole skeleton and that is the reason to include the factor $\frac{p_{0}}{p\left(t\right)}$ in Equations 55 and 57, with $p_{0}$ being the initial or reference porosity. The factor $\frac{V\left(t,i\right)}{V_{b}\left(t\right)}$ ensures that the elements of the queue with a larger volume can store more mineral, while the limiting factor $\left(1-\frac{v_{m}\left(t,i\right)}{v_{m}^{\max}}\right)$ recovers the idea of saturation from Equation 49.

Fluxes $J_{15-13}$ and $J_{16-14}$ are due to bone resorption, which dissolves the mineral and puts it back into the bone marrow. $J_{15-13}$ is given in Equation 59 by the sum of the Ca amounts removed from each element of the “calcium queue” (see Equation 52):

$$J_{15-13}\left(t\right)=\underset{i}{\sum }A_{15}\left(t,i\right)\;\frac{\Delta V_{r}\left(t\right)}{V_{b}\left(t\right)}\;f_{SL}$$ (59)

and $J_{16-14}$ is given in Equation 60 by the stoichiometric ratio:

$$J_{16-14}\left(t\right)=0.464\cdot J_{15-13}\left(t\right)$$ (60)

The mineral content of the RVE is calculated through Equation 61, which is analogous to Equation 53, but now summing the mineral content of each element of the queue.

$$v_{m}\left(t\right)=\frac{\sum_{i}A_{15}\left(t,i\right)}{\sum_{i}V\left(t,i\right)}\cdot \frac{M_{HA}}{\rho_{m}\cdot n_{Ca,HA}}$$ (61)

This value is replaced in Equations 45 and 46 to obtain the tissue density and the apparent density.

### 2.5 Simulation of diseases

CKD was simulated, as in (Peterson and Riggs, 2010), with a parabolic decrease in GFR over time (Equation 62), now lasting 5 years, from an initial (normal) value of $GFR_{0}=6L/h$ to $GFR_{min}$ , which was varied to simulate different scenarios of CKD severity.

$$GFR\left(t\right)=\left\{\begin{array}{cc} GFR_{0} & \;\;\;\;\;\;\;\;\;\;\;\;if\;t<t_{\text{onset}}\\ \begin{array}{cc} & \left(GFR_{0}-GFR_{\min}\right)\;{\left(\frac{t-t_{\mathrm{onset}}}{5}\right)}^{2}-\\ & 2\;\left(GFR_{0}-GFR_{\min}\right)\;\left(\frac{t-t_{\mathrm{onset}}}{5}\right)+GFR_{0}\\ GFR_{min}\;\;\;\;\;\;\;\;\;\;\;if\;t\geq t_{onset}+5 \end{array}\;if\;t_{\text{onset}}\leq t<t_{\mathrm{onset}}+5 \end{array}\right.$$ (62)

where $t_{onset}$ is the time at the onset of the disease and t is measured in years.

pHPT was simulated through Equation 63 as an abnormal growth of the PT glands, modelled by the term $k_{HPT}$ (Equation 32), which was increased over time using a sigmoid function:

$$k_{\text{HPT}}\left(t\right)=\left\{\begin{array}{cc} 0 & \;if\;t<t_{\text{onset}}\\ k_{\mathrm{HPT}}^{\max}\;\frac{{\left(t-t_{\mathrm{onset}}\right)}^{\gamma_{\mathrm{HPT}}}}{{\left(t-t_{\mathrm{onset}}\right)}^{\gamma_{\mathrm{HPT}}}+{\left(\delta_{\mathrm{HPT}}\right)}^{\gamma_{\mathrm{HPT}}}} & \;if\;t\geq t_{onset} \end{array}\right.$$ (63)

where $\delta_{HPT}=1/2$ year and $\gamma_{HPT}=0.1$ was fitted to produce an abrupt growth of PT-glands at the onset of the disease. The maximum growth rate of PT-glands $k_{HPT}^{max}$ was adjusted to produce serum PTH levels in accordance with clinical data (Rubin et al., 2008; Silverberg et al., 1999).

### 2.6 Calcium, ${\text{PO}}_{4}$ and vitamin D intake

The following diet-related scenarios have been analysed in our simulations: 1,2) Ca-poor or Ca-rich diet, 3,4) P-poor or P-rich diet, and 5,6) vitamin D deficiency or supplementation. These scenarios have been analysed in three conditions: healthy case, CKD, and HPT.

The assumed normal daily Ca intake was ${\text{D}}_{1}$ = 25 mmol/day (Strewler, 2003). Two degrees of Ca intake deficiency were simulated, with a 6 and 12 mmol/day decrease from the normal intake ( ∼25% and ∼50% ). These values were selected according to Warensjö et al. (2011) who detected a higher incidence of fracture and prevalence of osteoporosis in postmenopausal women with a daily Ca intake lower than 19 mmol/day (∼−25%) . Ca supplementary intake is based on pills of 600 mg of calcium carbonate (6 mmol of Ca). The recommended dose for osteoporotic patients is 1 or 2 pills per day, which result in increments of ∼ 25% or ∼ 50% of the normal daily Ca intake. We have simulated both cases.

Changes in vitamin D intake (or production) were simulated by a change in $k_{25-OH,D}^{nom}=1.0$ (see Equation 27), with $k_{25-OH,D}=1.25$ to simulate vitamin D supplementation and $k_{25-OH,D}=0.75$ and $k_{25-OH,D}=0.55$ to simulate, respectively, mild and strong vitamin D deficiency. More precisely, $k_{25-OH,D}=0.55$ was adjusted to produce the calcitriol levels reported by Need et al. (2008) in patients with severe vitamin D deficiency. A summary of the constants used to simulate the diseases and the diet-related scenarios is given in Table 2.

**TABLE 2:** Summary of the constants used to simulate homeostasis (normal), chronic kidney disease (CKD) and hyperparathyroidism (HPT) together with different diet-related interventions.

Scenario	Case	Parameter	Value	Units
Renal condition	Normal	$GFR_{min}$	6	L/h
	Mild CKD - Group 4 [42]	​	4.98	L/h
	Mild/Intermediate CKD - Group 3 [42]	​	3.48	L/h
	Intermediate CKD - Group 2 [42]	​	2.34	L/h
	Severe CKD - Group 1 [42]	​	0.96	L/h
Status of PT glands	Normal	$k_{HPT}^{max}$	0	${\text{h}}^{-1}$
	Mild HPT	​	$2.0\cdot 10^{-3}$	${\text{h}}^{-1}$
	Severe HPT	​	$4.7\cdot 10^{-3}$	${\text{h}}^{-1}$
Ca intake	Normal	${\text{D}}_{1}$	25	mmol/day
	Deficiency (-Ca)	​	19	mmol/day
	Severe deficiency (-- Ca)	​	13	mmol/day
	Supplement (+Ca)	​	31	mmol/day
	Strong supplement (++ Ca)	​	37	mmol/day
${PO}_{4}$ intake	Normal	${\text{D}}_{3}$	45.2	mmol/day
	Decreased intake (– ${\text{PO}}_{4}$ )	​	22.6	mmol/day
	Increased intake (++ ${\text{PO}}_{4}$ )	​	67.7	mmol/day
Vitamin D	Normal	$k_{25-OH,D}$	1	$h^{-1}$
	Mild deficiency (-vit D)	​	0.75	${\text{h}}^{-1}$
	Severe deficiency (--vit D)	​	0.55	${\text{h}}^{-1}$
	Supplement (+vit D)	​	1.25	${\text{h}}^{-1}$

### 2.7 Summary of simulations

Using the model presented above, we have performed the following simulations to analyse the coupling between the regulation of Ca/P homeostasis and the process of bone remodelling.

- In Experiment 1 (abbreviated Exp*1*), we studied the influence of the applied load and the mineralisation rate, $k_{13-15}$ , on the evolution of bone porosity and apparent density.
- In Exp*2*, we analyzed vitamin D deficiency and compared our *in-silico* results with the clinical data reported by Need et al. (2008).
- In Exp*3*, we studied the influence of CKD on different biomarkers through the variation of GFR (see Equation 62) and compared our *in-silico* results with the clinical study from Rix et al. (1999).
- In Exp*4*, we studied pHPT through the abnormal growth of PT glands Equation 63 and adjusted the constant $k_{HPT}^{max}$ to obtain *in-silico* results similar to clinical results on this disease (Rubin et al., 2008; Silverberg et al., 1999).

The experiments above were used to calibrate our model. The following experiments analyse dietary changes alone and in concurrence with CKD and pHPT.

- In Exp*5*, we analysed how dietary changes (Ca, ${\text{PO}}_{4}$ and vitamin D) can alter Ca/P homeostasis and bone density in healthy subjects.
- In Exp*6* we studied the influence of severe CKD (Group 1 (Rix et al., 1999), see Table 2) on bone density and the effect of dietary changes to combat the disease.
- The same study was replicated in Exp*7*, focusing on patients with pHPT.

All experiments started from a homeostasis state with the initial conditions specified in Table 1 which were chosen as explained in the Supplementary Material.

## 3 Results

**FIGURE 3:**
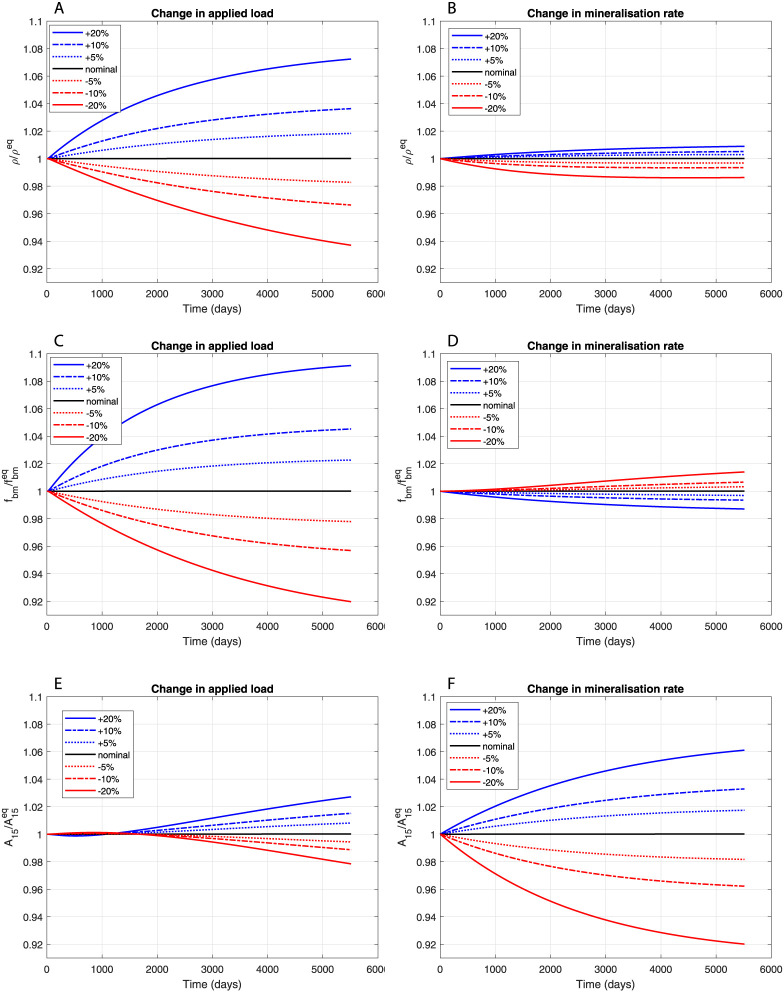
Results of Exp*1*. Temporal evolution of apparent density **(A,B)**, bone volume fraction, $f_{bm}$ **(C,D)** and Ca contained in the skeleton **(E,F)** under changes in the applied load and in the mineralisation rate $k_{13-15}$ .

**FIGURE 4:**
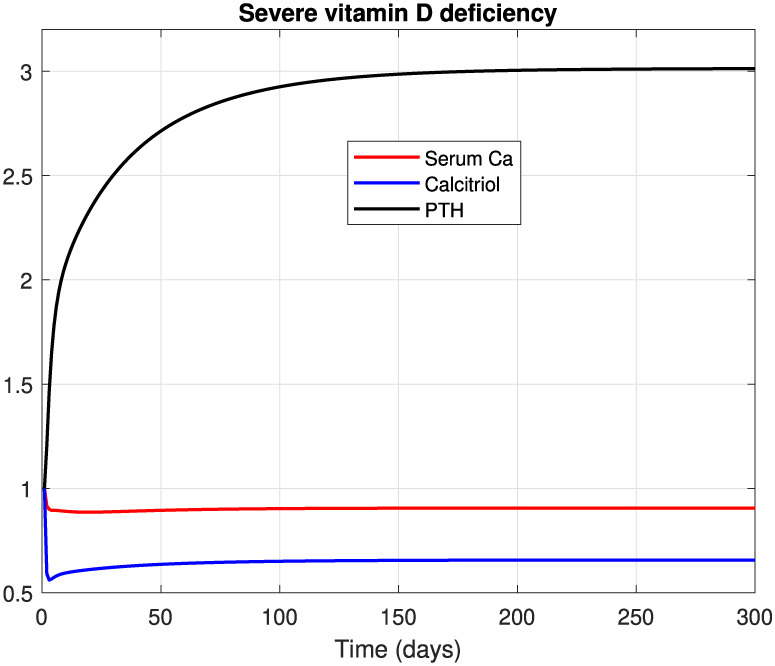
Results of Exp*2*. Temporal evolution of serum Ca, calcitriol and PTH in a case of severe vitamin D deficiency (-- vit D, $k_{25-OH-D}=0.55$ ).

**FIGURE 5:**
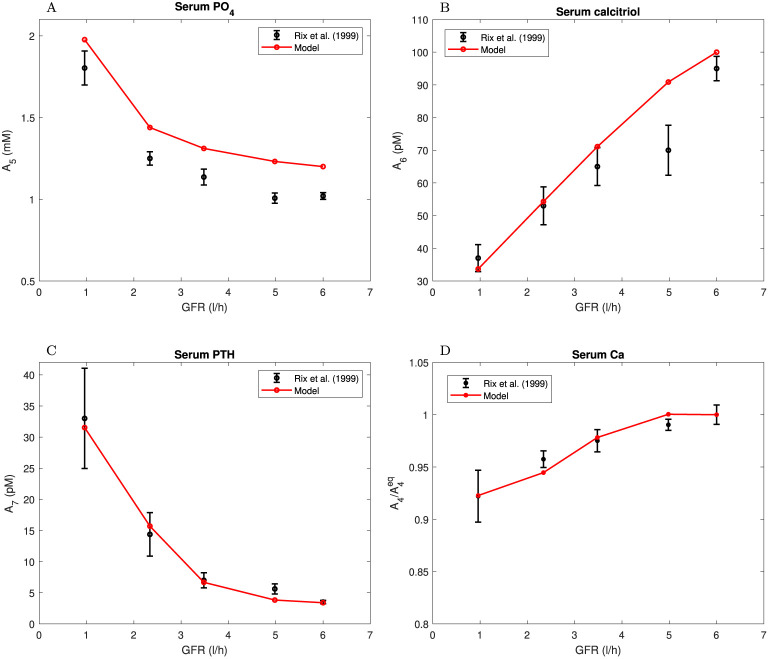
Results of Exp*3*. Comparison of *in-silico* results (after 15 years of simulation) with serum concentrations of ${\text{PO}}_{4}$ **(A)**, calcitriol **(B)**, PTH **(C)** and Ca **(D)** measured by Rix et al. (1999) in patients with different GFR.

**FIGURE 6:**
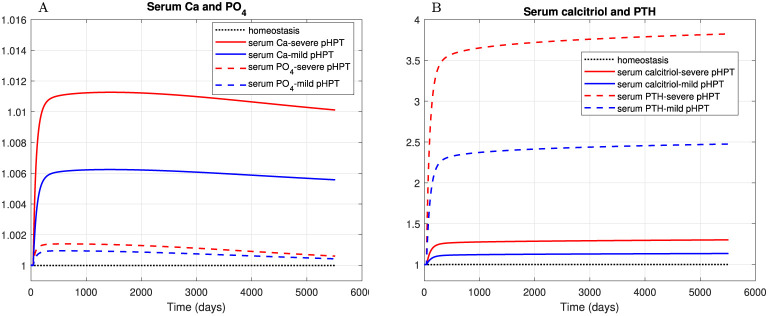
Results of Exp*4*. Temporal evolution of serum calcitriol and PTH **(A)** and serum Ca and ${\text{PO}}_{4}$ **(B)** predicted by the model in patients with primary hyperparathyroidism (pHPT).

As stated above, we conducted Exp*1* to Exp*4* to calibrate our model. This was an iterative process that is briefly summarised in Table 3. Figures 3–6 show the results of the experiments used in the calibration process, for the definitive (calibrated) constants, which can be consulted in the Supplementary Material.

**TABLE 3:** Summary of the calibration procedure.

Step	Exp	Calibrated variables	Goal of the calibration. Comments
1	Exp*1*	Load, $k_{13-15}$	Homeostasis is achieved, i.e., no changes in porosity and apparent density are obtained if the simulation starts from a homeostatic situation, characterised by $f_{bm}=44\%$ and $A_{15}^{eq}=25725$ mmol (Goldstein, 1990; Cashman, 2002). Figure 3 shows the influence of load and $k_{13-15}$ on apparent density, $f_{bm}$ and skeletal Ca $\left(A_{15}\right)$ . Nominal stands for the set of calibrated constants that maintains homeostasis
2	Exp*2*	$k_{25-OH,D}$ and constants of $H_{6,2}^{\pm }$	Reproducing the clinical results from Need et al. (2008) on individuals with severe vitamin D deficiency. $k_{25-OH,D}=0.55$ was adjusted to produce a serum calcitriol concentration equal to 64% of the normal value (Need et al., 2008) (Figure 4). The function $H_{6,2}^{\pm }$ (Equation 5) was adjusted to obtain a 40% reduction in Ca intestinal absorption $\left(J_{1-4}\right)$ due to calcitriol deficiency (Need et al., 2008). This resulted in serum Ca concentrations in the range 2.17–2.22 mM (around 10% reduction with respect to normal, Figure 4), while those authors measured 2.22 ± 0.2 mM for the severe deficiency group
3	Exp*3*	Constants of $H_{5,10}^{-}$ , $H_{7,10}^{+}$ , $T_{6,4}^{\pm }$ , $H_{6,4-u}^{-}$ and $k_{4-u,reabs}\left(A_{4}\right)$	Reproducing the clinical results of Rix et al. (Rix et al., 1999) in CKD patients. Figure 5 shows the calibration results of ${\text{PO}}_{4}$ , calcitriol, PTH, and Ca. GFR was too high (8.64 L/h) for the control group in (Rix et al., 1999), since healthy normal values of GFR are within 5.4–7.2 L/h (GFR above 7.2 L/h is considered hyperfiltration (Ruggenenti et al., 2012)). For this reason, we adjusted GFR of the control group to 6 L/h
4	Exp*2*	Constants of $H_{6,12}^{-}$	Although $H_{6,12}^{-}$ also affects Exp*3*, it was kept constant in Step 3 and calibrated here to reproduce the sHPT observed in vitamin D deficient patients (see Figure 4). This PTH increase cannot be explained by a higher activity of PT chief cells alone (i.e., $T_{6,4}^{\pm }$ ), as PTH would be limited to a 2-fold increase (see Equations 30 and 31), while the clinical study reported a 3-fold increase (Need et al., 2008). That is the reason to include $H_{6,12}^{-}$ in Equation 32, to account for hyperplasia of PT glands in vitamin D-deficiency (Slatopolsky, 1998)
5	Exp*4*	$k_{HPT}^{max}$	$k_{HPT}^{max}=4.7\cdot 10^{-3}\;h^{-1}$ was fitted to simulate PT glands hyperplasia due to pHPT, causing a x3.8 increase in serum PTH w.r.t. the normal value [38, 39]. A lower value, $k_{HPT}^{max}=2.0\cdot 10^{-3}\;h^{-1}$ was also used to simulate mild pHPT, leading to a x2.5 PTH increase (Figure 6B)
6	​	​	Steps 2-5 were repeated as the involved constants are interrelated

**FIGURE 7:**
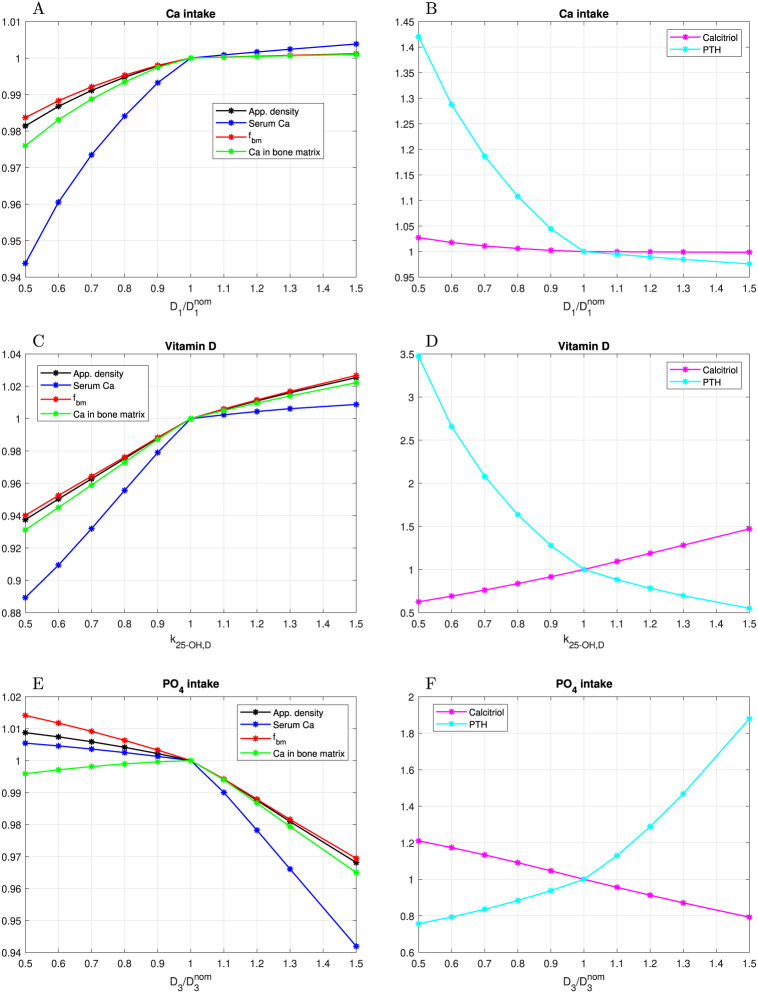
Results of Exp*5*. Influence of dietary changes on healthy individuals. Ca intake ( ${\text{D}}_{1}$ ), calcidiol availability $k_{25-OH,D}$ , and ${\text{PO}}_{4}$ intake ( ${\text{D}}_{3}$ ) were changed proportionally to their nominal values. The results shown (normalized w.r.t. initial values) correspond to 5 years of dietary change. Influence of dietary changes on apparent density, serum Ca, f_bm_, and Ca contained in bone matrix (**A,C,E**) and on serum calcitriol and PTH (**B,D,F**).

Once the model was calibrated, we analysed dietary changes in healthy subjects (Exp*5*) and the results are summarised in Figure 7. This figure represents the values of a number of variables reached after 5 years of dietary change, normalized with respect to the value obtained for a normal diet. It can be seen that a Ca intake above normal has a negligible effect on the results and vitamin D supplementation only a minor effect. In contrast, deficiencies of both could have a strong and negative impact on bone and serum Ca levels. ${\text{PO}}_{4}$ intake has the opposite effect, as an increased intake seems detrimental for bone, while a reduction has a slight though beneficial effect.

**FIGURE 8:**
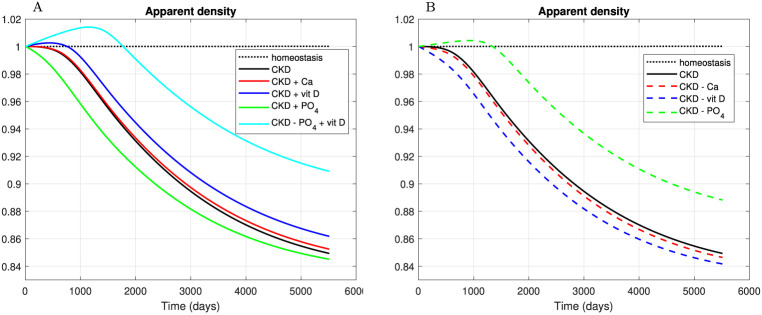
Results of Exp*6* – Temporal evolution of apparent density during simulations of severe CKD. Comparison of the effect of different dietary supplements **(A)** and insufficient diets **(B)**.

Figure 8 analyses the results of Exp*6*, i.e., the bone response to severe CKD (Group 1 of (Rix et al., 1999), see Table 2) under several dietary scenarios. Figure 8A shows the cases involving dietary supplements and Figure 8B the cases involving insufficient diets. The first observation is a serious loss of bone mass during the disease (solid black line), of around 15% over 15 years, as a consequence of the sHPT that CKD produces (recall Figure 5C). Changes in calcium intake have barely any incidence on bone mass, neither in one direction nor the other. Variations in vitamin D have a slightly greater incidence, but are also irrelevant. What does significantly affect bone mass is the change in ${\text{PO}}_{4}$ intake. An increase has a slight counterproductive effect, but the most interesting finding is that the reduction in ${\text{PO}}_{4}$ intake intake notably alleviates bone loss (dashed green line), especially if accompanied by a vitamin D supplement (solid light blue line), since both actions manage to counteract the main cause of bone loss: the decrease in calcitriol.

**FIGURE 9:**
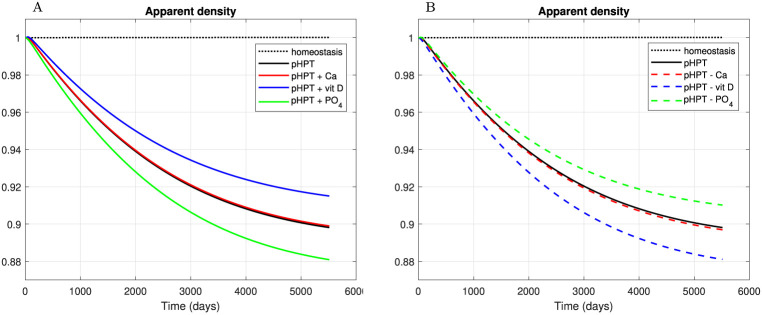
Results of Exp*7* – Temporal evolution of apparent density during simulations of pHPT. Comparison of the effect of different dietary supplements **(A)** and insufficient diets **(B)**.

Figure 9 shows the results of Exp*7*, i.e., the bone response to severe pHPT (see Table 2) under dietary changes. With a normal diet, the simulated bone loss approximates 10%. Once again, the effect of Ca intake on bone loss is negligible, whereas changes in vitamin D and ${\text{PO}}_{4}$ intake now exhibit a greater effect than in CKD, with variations of approximately 2%. Specifically, supplementing vitamin D and reducing ${\text{PO}}_{4}$ intake is beneficial, and *vice versa*.

## 4 Discussion

The coupled model developed in this work allowed us to investigate the relationship between dietary changes and certain bone diseases. This connection is established through the regulation of Ca/P homeostasis, with bone remodelling being one of the key underlying mechanisms.

The model was calibrated to ensure Ca/P homeostasis through the balance of all considered endocrine factors, including an equilibrium of the bone remodelling process that maintained constant BMD under a specific mechanical stimulus. Additionally, the model was calibrated to reproduce clinical results of vitamin D deficiency (Need et al., 2008), pHPT (Rubin et al., 2008; Silverberg et al., 1999) and CKD (Rix et al., 1999). All these objectives were met satisfactorily, except for the prediction of ${\text{PO}}_{4}$ levels in CKD. The results obtained in the simulation overestimate the clinical data, although they follow the same increasing trend as GFR decreases. This may be due to the simplified model of ${\text{PO}}_{4}$ homeostasis which followed the equations of Peterson and Riggs (2010). However, the effect of fibroblast growth factor-23 (FGF-23) on the regulation of ${\text{PO}}_{4}$ homeostasis was not considered despite its important role (Sá et al., 2011). This limitation may also explain why the model failed to simulate hypophosphatemia observed clinically in pHPT (Bhadada et al., 2024).

After calibration, dietary changes were analysed in healthy subjects (Figure 7). Serum Ca levels are tightly regulated by different mechanisms. One of these is calcitriol-driven intestinal absorption, which occurs via two mechanisms: the passive, non-saturable component (paracellular), which only accounts for 16% of the total (McCormick, 2002), and the active calcitriol-driven component, which is saturable, and does not increase much with Ca intake, but rather with calcitriol levels, that remain almost constant if Ca intake is increased. Therefore, a Ca-rich diet does not translate into a noticeable increase in the amount of Ca absorbed in the intestine and Ca is eliminated via faeces, with little effect on the rest of variables, particularly those related to bone (Figure 7A). More precisely, if $f_{bm}$ remains constant and extra Ca does not reach bone marrow, the limited mineralisation yields no increase in skeletal Ca.

A deficit in Ca intake causes a drop in serum Ca, which activates PTH and calcitriol production (Figure 7B), to enhance Ca intestinal absorption. The PTH increase boosts bone turnover to release Ca from bone matrix and compensate for the serum Ca drop. This leads to an increase in porosity and a long-term skeletal Ca loss, if Ca deficit is sustained over time. The value of Ca intake deficit (−25%) was chosen according to the study of Warensjö et al. (2011) who conducted a longitudinal study in a cohort of more than 60k women. The authors divided the cohort into quintiles according to Ca intake, with −25% being the limit of the lowest quintile. These authors reported a higher rate of first fractures and a prevalence of osteoporosis only in the lowest quintile, with no significant differences between the rest. That conclusion would be supported by our results that show a negligible influence of extra Ca intake on bone density (Figure 7A). In cases of Ca deficiency, the effect on bone density is less than 1% up to the limit of that quintile (−25% Ca intake) and more pronounced within that group (−50%), reflecting a higher risk of fracture in that quintile and no differences in the rest. These results were confirmed by a systematic review of the literature (Bolland et al., 2015), which found no clinical trial evidence that increased Ca intake from dietary sources prevented fractures. Cano et al. (2018) reached a similar conclusion, as intake below the recommended amount may increase fragility fracture risk; however, there is no consistent evidence that Ca supplementation at or above the recommended levels reduces this risk. From our *in-silico* trials, it can be deduced that it also does not improve bone density in patients with CKD and HPT (see Figures 8, 9).

Regarding vitamin D, our results showed that its influence on bone density is more pronounced than that of Ca intake, particularly in deficiency cases. Calcitriol decline reduces Ca absorption and therefore, Ca concentration in serum and bone marrow, thus limiting the amount of mineral available for bone mineralisation ( ${\text{A}}_{15}$ drops by 15%, see Figure 7C green line). The notable drop in serum calcitriol causes sHPT (Figure 7D), as confirmed in (Need et al., 2008). sHPT has also been confirmed in Ca deficiency (Agarwal et al., 2009), but is more noticeable in vitamin D deficiency, according to our results (x3.5 against x1.5). This sHPT enhances bone resorption and its concurrence with reduced mineralisation produces a gradual but steady Ca loss in the skeleton, up to 7% in 5 years in severe deficiency (Figure 7C).

Vitamin D supplementation has a slightly greater effect than Ca-rich diet, due to the increase in calcitriol concentration. This increases Ca intestinal absorption and lowers PTH levels, which, in turn, slows down bone turnover, leading to a reduction in porosity. Although serum Ca does not increase very notably, due to the saturation of intestinal absorption, its small rise, combined with the increase in the amount of bone matrix, allows for greater mineral accumulation in the skeleton and an increase in bone density.

The benefit of vitamin D supplementation on bone density is not only noticeable in healthy people, but even more so in people suffering from the diseases analysed here, especially pHPT ( 1.7% increase with respect to no supplementation, see Figure 9A). In this case, increased calcitriol levels substantially reduces PTH production and counteracts the disease and its effect on bone mass. The benefit of vitamin D supplementation on bone density is lower in CKD ( 1.2% increase with respect to no supplementation). In this case, the vitamin D supplementation of 25% (+vit D) is insufficient to restore calcitriol levels.

Phosphorus intake has a considerable impact on bone density. An excess of phosphorus has notorious negative effects, while a reduction has positive, though limited, impact on healthy subjects. ${\text{PO}}_{4}$ -rich diet reduces calcitriol production in the kidney and this causes sHPT (Figure 7F), which in turn, increases bone resorption and/or decreases bone formation (Vorland et al., 2017), leading to moderate bone loss (around 3% in 5 years, see Figure 7E; and 5% in 15 years, see Supplementary Material). This issue is particularly important in CKD patients (with abnormally high ${\text{PO}}_{4}$ levels), who are advised to reduce phosphorus intake (Raju and Saxena, 2025). Our simulations show that reducing ${\text{PO}}_{4}$ intake plus vitamin D supplementation has a synergistic effect on restoring calcitriol levels: 1.2% increase, if vitamin D supplementation is used; 3.9% increase, if ${\text{PO}}_{4}$ intake is reduced by 50%; and 6% when both interventions are implemented (see Figure 8).

Patients with CKD suffer from what has been termed CKD-mineral bone disorder (CKD-MBD), a set of biochemical alterations that begin in the earliest stages of CKD and worsen with progressive kidney disease (Isakova et al., 2011). These include elevated FGF-23, which may be a sensitive early biomarker of disordered phosphorus metabolism (Isakova et al., 2011), but was not considered in our model. This may trigger the rest of biochemical alterations: increased PTH levels, decreased calcitriol, severe hyperphosphataemia and hypocalcaemia (Hill-Gallant and Spiegel, 2017). After calibration, our model was able to capture all of these effects (see Figure 5), in contrast to Peterson and Riggs (2010), whose model could not predict hypocalcaemia and only predicted minor hyperphosphataemia.

We simulated pHPT through an abnormal growth of the PT glands (proliferation of PT chief cells) during 5 years, leading to increased PTH production and consequent bone loss. Rubin et al. (2008) performed a 15-year prospective longitudinal follow-up study in patients with pHPT. This study was quite comprehensive and measured different variables that can be used to validate the results of Figure 9. Bone loss after 15 years varied from 5% on average in the lumbar spine to 10% in the femoral neck, which is similar to our results (Figure 9); however, it was significantly higher in the radius ( ∼ 35%).

Our calibration of Exp*4* allowed us to improve the simulations of pHPT compared to the results of Peterson and Riggs (2010), at least regarding the calcitriol concentration. Our trials predicted an increase of 30% relative to the mean value of the normal range, which is close to the value of 50% value observed clinically (Rubin et al., 2008; Silverberg et al., 1999), while those authors predicted no significant changes. The increase in serum Ca predicted in (Peterson and Riggs, 2010) was 15%, which is higher than the clinically measured average increase of 6% (Rubin et al., 2008; Silverberg et al., 1999), which in turn is higher than our prediction of 1%. In both models, only small variations (slightly above normal) are obtained in the serum ${\text{PO}}_{4}$ concentration, while clinical studies indicate hypophosphatemia (Bhadada et al., 2024). This may be due to the fact that the ${\text{PO}}_{4}$ homeostasis model might be oversimplified by not considering the regulatory effect of FGF-23.

Regarding the effect of dietary changes in pHPT patients, it is again observed that Ca intake has little influence on bone density. The vitamin D supplement has a slightly greater effect than in CKD patients (an increment of 1.7% compared to the no-diet-change case), while the decrease in ${\text{PO}}_{4}$ intake has a much smaller effect now (an increment of only 1.2%).

This study has several limitations, discussed below, primarily arising from necessary model simplifications. Future work addressing these points will be essential to enhance predictive reliability and broaden the model’s applicability. This future work includes: 1) the consideration of ageing in bone response, e.g., following the procedure described by Ruiz-Lozano et al. (2014), 2) a revision of the simplified model of ${\text{PO}}_{4}$ homeostasis and the omission of FGF-23; and 3) the simplistic way of modelling vitamin D metabolism proposed by Peterson and Riggs (2010). This was corrected only partially by introducing the factor $k_{25-OH,D}$ in Equation 27, but will need further revision.

The bone response at the RVE under study was extrapolated to the skeleton by assuming that local bone turnover in the RVE is the same, on average, as in the entire skeleton, except for a correction that considers the differences in bone turnover of cortical and trabecular bone and the average amount of each type of tissue in the skeleton (see Supplementary Material). This correction assumes that bone turnover is uniform across the skeleton, which is not true, and should be nuanced to overcome that limitation in order to bridge local bone remodelling and systemic outputs.

We only considered osteoclastic resorption and osteoblastic formation. However, this is not the only bone turnover mechanism. Osteocytes can also resorb the perilacunar matrix, in a process known as osteocytic osteolysis (Tsourdi et al., 2018), and to replace the removed matrix (Wysolmerski, 2013). This mechanism of osteocytic osteolysis can be very relevant to control Ca/P homeostasis as it is quicker and, therefore, it could be more efficient to buffer variations in serum levels than osteoclastic resorption.

Calcitonin is a hormone secreted by the C-cells of the thyroid gland in response to high serum Ca levels to prevent hypercalcaemia. Calcitonin has an immediate effect on decreasing osteoclast activity and also stimulates renal calcitriol production (Felsenfeld and Levine, 2015). This gives it a fundamental role in the control of Ca homeostasis and makes its inclusion in future versions of the model mandatory.

Equation 41 represents a considerable simplification of the model, as it implies that the density of osteocytes contained in the bone matrix is constant, when in reality there may be inter-subject differences and, more importantly, it may decrease with age, which also causes deterioration of the canalicular network reducing the ability of osteocytes to transport factors to the bone surface (Yılmaz et al., 2025).

Finally, it should be noted that the present framework focuses primarily on the bone-serum exchange axis and does not explicitly account for extra-skeletal mineral deposition. Under conditions of persistent hypercalcemia or elevated calcium-phosphate products—common in advanced CKD and pHPT—minerals may accumulate in non-osseous tissues. These pathological processes, which include vascular and cardiac valve calcification, nephrocalcinosis, and metastatic calcification in soft tissues, act as unregulated mineral sinks. While beyond the current scope of this work, incorporating these secondary compartments in future iterations would allow for a more comprehensive analysis of how systemic mineral dysregulation affects both skeletal integrity and cardiovascular health. In conclusion, this work presents an integrated modelling framework that couples systemic Ca/P homeostasis with a mechanistic bone cell population model, enabling quantitative analysis of how endocrine dysregulation, renal impairment, and dietary modifications influence bone remodelling. The model successfully reproduces clinical trends in vitamin D deficiency, CKD, and HPT, and provides new insights into how changes in calcium, phosphate, and vitamin D intake affect both mineral homeostasis and skeletal health. Therefore, this framework offers a valuable tool for exploring disease mechanisms and evaluating potential therapeutic or nutritional interventions.

## Data Availability

The original contributions presented in the study are included in the article/Supplementary Material, further inquiries can be directed to the corresponding author.

## Footnotes

1 The homeostatic values are designated as $A_{i}^{eq}$ and are equal to the initial values $\left(A_{i}^{eq}=A_{i}\left(t=0\right)\right)$ since all simulations start from a homeostatic situation.

2 We recall that variable ${\text{A}}_{4}$ represents the amount of Ca in blood plasma. Hence, the concentration, like all other serum concentrations, is obtained by dividing by $V_{\mathrm{plasma}}=3L$ (Hahn and Zdolsek, 2025).

3 It is important to note that fluxes are unidirectional, i.e., negative fluxes are not allowed, and if this occurs, they are set equal to zero.
