# Supplementary material for: The interrelationship between calcium-phosphorus homeostasis and bone remodelling. Impact of dietary changes on bone in patients with primary hyperparathyroidism and chronic kidney disease
Source: Front Bioeng Biotechnol. 2026 Jun 23;14:1800350. doi: 10.3389/fbioe.2026.1800350 (PMC13365903; doi:10.3389/fbioe.2026.1800350)
Supplement: Supplementary file 1 [file DataSheet1.pdf]

Supplementary Material to: The interrelationship between calcium-phosphorus homeostasis and bone remodelling. Impact of dietary changes on bone in patients with primary hyperparathyrodism and chronic kidney disease

Javier Martínez-Reina[a,d], José Luis Calvo-Gallego[a,d], Rocío Ruiz-Lozano[a,d], Peter Pivonka[b], Ralph Müller[c,*]

[a]*Departmento de Ingeniería Mecánica y Fabricación, Universidad de Sevilla, Seville 41092, Spain*
[b]*School of Mechanical, Medical and Process Engineering, Queensland University of Technology, QLD 4000, Australia.*
[c]*Institute for Biomechanics, ETH Zurich, 8092 Zurich, Switzerland*
[d]*Instituto de Biomedicina de Sevilla (IBIS). C/ Antonio Maura Montaner, 41013 Sevilla, Spain*

---

1. Introduction

In this document, we explain in detail the bone cell population model (BCPM) developed in previous works (Section 2) and coupled to the Ca/P homeostasis model in the current work. We also comment some changes introduced in the Ca/P homeostasis model with respect to the model developed by Peterson and Riggs [1] and the procedure followed to adjust the constants of the model (Section 3). In Section 4, we define a factor used in the model to establish the relationship between local and systemic variables. Finally, we show other results that are not shown in the main document (Section 5) and provide the constants of the model (Section 6).

2. Bone cell population model (BCPM)

2.1. Competitive binding in BCPM

Many biological processes are controlled by binding of biochemical factors which act as receptor and ligand. In some of them two or more ligands compete to bind to the receptor. This is the case of Wnt and sclerostin that compete to bind to LRP5/6 to control the proliferation of osteoblast precursors and also the case of RANK and OPG which compete to bind to RANKL to control the differentiation of osteoclast precursors into mature active osteoclasts.

Let us consider separately the binding of a given receptor R to its ligands A and B to form, respectively, the complexes A–R and B–R. Let us consider for each species X=A,B,R a production term $\mathrm{P_X}$ and a degradation term $\mathrm{D_X}$, along with a degradation term for the complex $\mathrm{D_{X-Y}}$. Let $\mathrm{K^r_{X-Y}}$ and $\mathrm{K^f_{X-Y}}$ be the reverse and forward binding reaction constants, respectively.

$$\begin{array}{ccccccc} \downarrow \mathrm{P_R} & & \downarrow \mathrm{P_A} & & & & \\ \mathrm{R} & + & A & \underset{\mathrm{K^r_{A-R}}}{\overset{\mathrm{K^f_{A-R}}}{\rightleftharpoons}} & \mathrm{A-R} & \overset{\mathrm{D_{A-R}}}{\rightharpoonup} & \emptyset \\ \downarrow \mathrm{D_R} & & \downarrow \mathrm{D_A} & & & & \end{array} \tag{1}$$

---

*Corresponding author: jmreina@us.es
Email addresses: jmreina@us.es (Javier Martínez-Reina), joselucalvo@us.es (José Luis Calvo-Gallego), rrlozano@us.es (Rocío Ruiz-Lozano), peter.pivonka@qut.edu.au (Peter Pivonka), ram@ethz.ch (Ralph Müller)

$$\begin{array}{ccccccc} \downarrow P_R & & \downarrow P_B & & & & \\ R & + & B & \underset{K^r_{B-R}}{\overset{K^f_{B-R}}{\rightleftharpoons}} & B-R & \overset{D_{B-R}}{\rightharpoonup} & \emptyset \\ \downarrow D_R & & \downarrow D_B & & & & \end{array} \tag{2}$$

The law of mass action provides the following set of differential equations:

$$\frac{d\,[A-R]}{dt} = K^f_{A-R}\,[A]\,[R] - K^r_{A-R}\,[A-R] - \tilde{D}_{A-R}\,[A-R] \tag{3a}$$

$$\frac{d\,[B-R]}{dt} = K^f_{B-R}\,[B]\,[R] - K^r_{B-R}\,[B-R] - \tilde{D}_{B-R}\,[B-R] \tag{3b}$$

$$\frac{d\,[A]}{dt} = P_A - \tilde{D}_A\,[A] + K^r_{A-R}\,[A-R] - K^f_{A-R}\,[A]\,[R] \tag{3c}$$

$$\frac{d\,[B]}{dt} = P_B - \tilde{D}_B\,[B] + K^r_{B-R}\,[B-R] - K^f_{B-R}\,[B]\,[R] \tag{3d}$$

$$\frac{d\,[R]}{dt} = P_R - \tilde{D}_R\,[R] + K^r_{A-R}\,[A-R] + K^r_{B-R}\,[B-R] - K^f_{A-R}\,[A]\,[R] - K^f_{B-R}\,[B]\,[R] \tag{3e}$$

where $[X]$ and $[X-Y]$ represent, respectively, the concentration of species X and complex X-Y. The degradation terms are assumed proportional to the concentration of the species, i.e. $D_X = \tilde{D}_X\,[X]$, with $\tilde{D}_X$ being the degradation rate.

Following Pivonka et al. [2] we assume that the binding reactions are much faster than the cell responses they produce and hence a quasi-steady state can be assumed, implying that the time derivatives of Eqs. (3) are null. This condition in Eqs. (3a) and (3b) yield:

$$[A-R] = \frac{[A]\,[R]}{K_{A-R}} \tag{4a}$$

$$[B-R] = \frac{[B]\,[R]}{K_{B-R}} \tag{4b}$$

where:

$$K_{A-R} = \frac{K^r_{A-R} + \tilde{D}_{A-R}}{K^f_{A-R}} \tag{5a}$$

$$K_{B-R} = \frac{K^r_{B-R} + \tilde{D}_{B-R}}{K^f_{B-R}} \tag{5b}$$

The stationarity condition of Eqs. (3c)-(3e) yields:

$$[A] = \frac{P_A}{\tilde{D}_A + \frac{\tilde{D}_{A-R}}{K_{A-R}}\,[R]} \tag{6a}$$

$$[B] = \frac{P_B}{\tilde{D}_B + \frac{\tilde{D}_{B-R}}{K_{B-R}}\,[R]} \tag{6b}$$

$$[R] = \frac{P_R}{\tilde{D}_R + \frac{\tilde{D}_{A-R}}{K_{A-R}}\,[A] + \frac{\tilde{D}_{B-R}}{K_{B-R}}\,[B]} \tag{6c}$$

If the degradation ($\tilde{D}_X$,$\tilde{D}_{X-Y}$), production (P_X) and dissociation constants, (K_{X-Y}) are known, Eqs. (6) constitutes a non-linear system of three equations with three unknowns, namely [A], [B], [R].

The total concentration of a receptor is the sum of the concentrations of the receptor which is found free and bound to ligands, i.e.:

$$[\mathrm{R}]_{\mathrm{tot}} = [\mathrm{R}] + [\mathrm{A}-\mathrm{R}] + [\mathrm{B}-\mathrm{R}] = [\mathrm{R}]\left(1 + \frac{[\mathrm{A}]}{\mathrm{K}_{\mathrm{A-R}}} + \frac{[\mathrm{B}]}{\mathrm{K}_{\mathrm{B-R}}}\right) \tag{7}$$

where Eqs. (4) have been used. The stationarity condition is equivalent to establish that the production rate of a species must equal the degradation rate, including all its forms, free and bound. For instance, in the case of the receptor that can bind to different ligands, this condition reads:

$$\mathrm{P}_{\mathrm{R}} = \tilde{\mathrm{D}}_{\mathrm{R}}\,[\mathrm{R}] + \sum_{\mathrm{L}} \tilde{\mathrm{D}}_{\mathrm{L-R}}\,[\mathrm{L}-\mathrm{R}] \tag{8}$$

which can be obtained from Eqs. (3a), (3b) and (3e) by imposing that the stationarity condition is met, ($\frac{\mathrm{d}\,[\mathrm{R}]}{\mathrm{dt}} = \frac{\mathrm{d}\,[\mathrm{L-R}]}{\mathrm{dt}} = 0 \quad \forall\, \mathrm{L} = \mathrm{A}, \mathrm{B}$). In the case of a ligand, that only binds to the receptor, that condition reads:

$$\mathrm{P}_{\mathrm{L}} = \tilde{\mathrm{D}}_{\mathrm{L}}\,[\mathrm{L}] + \tilde{\mathrm{D}}_{\mathrm{L-R}}\,[\mathrm{L}-\mathrm{R}] \tag{9}$$

The degradation rates are usually assumed as constants but the production rates are modelled in a more complex way. For instance, the production rate of ligand L can be split into a term corresponding to endogenous production, $\mathrm{P}_{\mathrm{L,b}}$, and a term accounting for external dosage, $\mathrm{P}_{\mathrm{L,d}}$:

$$\mathrm{P}_{\mathrm{L}} = \mathrm{P}_{\mathrm{L,b}} + \mathrm{P}_{\mathrm{L,d}} \tag{10}$$

The endogenous production is sometimes modelled by the following equation:

$$\mathrm{P}_{\mathrm{L,b}} = \sum_{\mathrm{X,Y}} \beta_{\mathrm{L,Y}}\, \pi^{\mathrm{X}}_{\mathrm{act/rep,Y}}\, \mathrm{Y}\left(1 - \frac{[\mathrm{L}]}{[\mathrm{L}]_{\mathrm{max}}}\right) \tag{11}$$

where Y is the concentration of the cell type Y producing L with a production rate $\beta_{\mathrm{L,Y}}$, regulated by the species X through the activator or repressor function $\pi^{\mathrm{X}}_{\mathrm{act/rep,Y}}$. The parenthesis establishes a saturation condition in such a way that ligand is not produced if its concentration reaches the maximum or saturation value, $[\mathrm{L}]_{\mathrm{max}}$.

As described by Pivonka et al. [3] the activation of a certain biological process regulated by the formation of the complex L–R is given by the ratio between the receptors R occupied by ligands L and the total number of ligands. In case of a single ligand the latter expressions yield the first-order Hill activator and repressor functions:

$$\pi^{\mathrm{L}}_{\mathrm{act,Y}} = \frac{[\mathrm{R}]}{[\mathrm{R}] + \mathrm{K}^{\mathrm{L-R}}_{\mathrm{act,Y}}} \tag{12}$$

$$\pi^{\mathrm{L}}_{\mathrm{rep,Y}} = \frac{\mathrm{K}^{\mathrm{L-R}}_{\mathrm{rep,Y}}}{\mathrm{K}^{\mathrm{L-R}}_{\mathrm{rep,Y}} + [\mathrm{R}]} \tag{13}$$

Alternatively, the cellular response could be defined as proportional to the fraction of receptors that are occupied by a certain ligand, as occurs in the Wnt-Scl-LRP5/6 signalling pathway. In this case:

$$\pi^{\mathrm{R}}_{\mathrm{act}} = \frac{[\mathrm{A}-\mathrm{R}]}{[\mathrm{R}] + [\mathrm{A}-\mathrm{R}] + [\mathrm{B}-\mathrm{R}]} \tag{14}$$

2.2. *Competitive RANK-RANKL-OPG binding*

The RANK-RANKL-OPG signalling pathway controls the differentiation of uncommitted osteoclast progenitors and osteoclasts maturation, respectively through $\pi^{\mathrm{RANK}}_{\mathrm{act,Oc_u}}$ and $\pi^{\mathrm{RANK}}_{\mathrm{act,Oc_p}}$:

$$\pi^{\mathrm{RANK}}_{\mathrm{act,X}} = \frac{[\mathrm{RANKL}]}{\mathrm{K}^{\mathrm{RANKL}}_{\mathrm{act,X}} + [\mathrm{RANKL}]} \quad \text{with } \mathrm{X} = \mathrm{Oc_u}, \mathrm{Oc_p} \tag{15}$$

with $\mathrm{K}^{\mathrm{RANKL}}_{\mathrm{act,X}}$ representing two constants corresponding to $\mathrm{X} = \mathrm{Oc_u}\,, \mathrm{Oc_p}$. Thus, an imbalance in that pathway, such as that occurring after menopause, may result in the development of osteoporosis. Following Martin et al. [4], the concentrations of OPG, RANK and RANKL are given by the following equations:

$$[\mathrm{OPG}] = \frac{\mathrm{P_{OPG}}}{\tilde{\mathrm{D}}_{\mathrm{OPG}} + \frac{\tilde{\mathrm{D}}_{\mathrm{OPG-RANKL}}\,[\mathrm{RANKL}]}{\mathrm{K_{OPG-RANKL}}}} \tag{16}$$

$$[\mathrm{RANK}] = \frac{\mathrm{N}^{\mathrm{RANK}}_{\mathrm{Oc_p}}\,\mathrm{Oc_p}}{1 + \frac{[\mathrm{RANK}]}{\mathrm{K_{RANK-RANKL}}}} \tag{17}$$

$$[\mathrm{RANKL}] = \frac{\mathrm{P_{RANKL}}}{\tilde{\mathrm{D}}_{\mathrm{RANKL}} + \frac{\tilde{\mathrm{D}}_{\mathrm{OPG-RANKL}}}{\mathrm{K_{OPG-RANKL}}} \cdot [\mathrm{OPG}] + \frac{\tilde{\mathrm{D}}_{\mathrm{RANK-RANKL}}}{\mathrm{K_{RANK-RANKL}}} \cdot [\mathrm{RANK}]} \tag{18}$$

where $\tilde{\mathrm{D}}_{\mathrm{X}}$ and $\tilde{\mathrm{D}}_{\mathrm{X-Y}}$ are the degradation rates of the factor X and the complex X-Y, respectively; $\mathrm{K_{X-Y}}$ is the dissociation constant of the complex X-Y and $\mathrm{N}^{\mathrm{RANK}}_{\mathrm{Oc_p}}$ is the number of RANK receptors per osteoclast precursor. $\mathrm{P_{OPG}}$ is the production rate of OPG by active osteoblasts and follows Eq. (11):

$$\mathrm{P_{OPG}} = \beta_{\mathrm{OPG,Ob_a}} \cdot \pi^{\mathrm{PTH}}_{\mathrm{rep,Ob_a}} \cdot \mathrm{Ob_a} \cdot \left(1 - \frac{[\mathrm{OPG}]}{[\mathrm{OPG}]_{\mathrm{max}}}\right) \tag{19}$$

where $\beta_{\mathrm{OPG,Ob_a}}$ is the OPG production rate, $\pi^{\mathrm{PTH}}_{\mathrm{rep,Ob_a}}$ is the repressor function that quantifies the effect of PTH on the production of OPG and $[\mathrm{OPG}]_{\mathrm{max}}$ is the saturation concentration of OPG above which no further production takes place. $\mathrm{P_{RANKL}}$ is the RANKL production rate in Eq. (18) and is given by:

$$\mathrm{P_{RANKL}} = \mathrm{P}^{\mathrm{RANKL}}_{\mathrm{Ob_p}} + \mathrm{P}^{\mathrm{RANKL}}_{\mathrm{Ot}} \tag{20}$$

Following [5, 6] we have assumed that RANKL is expressed by osteocytes ($\mathrm{P}^{\mathrm{RANKL}}_{\mathrm{Ot}}$) and osteoblast precursors ($\mathrm{P}^{\mathrm{RANKL}}_{\mathrm{Ob_p}}$) and these terms are given, respectively, by:

$$\mathrm{P}^{\mathrm{RANKL}}_{\mathrm{Ob_p}} = \beta_{\mathrm{RANKL,Ob_p}} \cdot \pi^{\mathrm{ED}}_{\mathrm{RANKL}} \cdot \pi^{\mathrm{PTH,NO}}_{\mathrm{act/rep,RANKL}} \cdot \left(1 - \frac{[\mathrm{RANKL}]_{\mathrm{tot}}}{[\mathrm{RANKL}]_{\mathrm{max}}}\right) \cdot \mathrm{Ob_p} \tag{21}$$

$$\mathrm{P}^{\mathrm{RANKL}}_{\mathrm{Ot}} = \beta_{\mathrm{RANKL,Ot}} \cdot \pi^{\mathrm{ED}}_{\mathrm{RANKL}} \cdot \pi^{\mathrm{dam}}_{\mathrm{act,RANKL}} \cdot \pi^{\mathrm{Scl}}_{\mathrm{act,RANKL}} \cdot \left(1 - \frac{[\mathrm{RANKL}]_{\mathrm{tot}}}{[\mathrm{RANKL}]_{\mathrm{max}}}\right) \cdot \mathrm{Ot} \tag{22}$$

where $[\mathrm{RANKL}]_{\mathrm{max}}$ is the maximum (or saturation) concentration of RANKL, $[\mathrm{RANKL}]_{\mathrm{tot}}$ is its total concentration (free and bound to its ligands), $\beta_{\mathrm{RANKL,Ob_p}}$ and $\beta_{\mathrm{RANKL,Ot}}$ are the RANKL production rates of each cell, $\pi^{\mathrm{PTH,NO}}_{\mathrm{act/rep,RANKL}}$ is a co-regulatory function that takes into account the upregulation of RANKL transcription by PTH and its inhibition by NO (Martin et al. [4]). $\pi^{\mathrm{dam}}_{\mathrm{act,RANKL}}$ is an activator function accounting for the upregulation of RANKL expression by osteocytes due to the presence of microstructural damage (Martínez-Reina et al. [7]). $\pi^{\mathrm{ED}}_{\mathrm{RANKL}}$ is an activator function that models the increase in RANKL expression due to the oestrogen deficiency occurring after menopause.

Finally, $[\mathrm{RANKL}]_{\mathrm{tot}}$ is given by the following:

$$[\mathrm{RANKL}]_{\mathrm{tot}} = [\mathrm{RANKL}] \cdot \left(1 + \frac{[\mathrm{OPG}]}{\mathrm{K_{OPG-RANKL}}} + \frac{[\mathrm{RANK}]}{\mathrm{K_{RANK-RANKL}}}\right) \tag{23}$$

2.3. Competitive binding Wnt–Scl–LRP5/6

The equations in section 2.1 can be used to describe the competitive binding Wnt–Scl–LRP5/6. Wnt signaling is an anabolic pathway promoting the proliferation of osteoblast precursors and hence bone formation. Extracellular Wnt binds to Frizzled and the lipoprotein receptor-related protein LRP5/6, so triggering intracellular activation of β-catenin. Sclerostin, produced by osteocytes, modulates the signaling pathway by its interaction with LRP5/6 receptors. This interaction prevents the formation of the Wnt-Frizzled-LRP5/6 complex and therefore hinders preosteoblasts proliferation. Competitive Wnt–Scl–LRP5/6 binding is modelled as follows. First, Eq. (7) reads for LRP5/6:

$$[\mathrm{LRP5/6}]_{\mathrm{tot}} = [\mathrm{LRP5/6}] \cdot \left(1 + \frac{[\mathrm{Wnt}]}{\mathrm{K}_{\mathrm{Wnt-LRP5/6}}} + \frac{[\mathrm{Scl}]}{\mathrm{K}_{\mathrm{Scl-LRP5/6}}}\right) \tag{24}$$

Following Martin et al. [4] we assumed that the total number of LRP5/6 receptors per osteoblast precursor ($\mathrm{N}_{\mathrm{OB_p}}^{\mathrm{LRP5/6}}$) is constant and thus:

$$[\mathrm{LRP5/6}]_{\mathrm{tot}} = \mathrm{N}_{\mathrm{OB_p}}^{\mathrm{LRP5/6}}\, \mathrm{Ob_p} \tag{25}$$

Solving for [LRP5/6] in Eq. (24):

$$[\mathrm{LRP5/6}] = \frac{\mathrm{N}_{\mathrm{OB_p}}^{\mathrm{LRP5/6}}\, \mathrm{Ob_p}}{1 + \frac{[\mathrm{Wnt}]}{\mathrm{K}_{\mathrm{Wnt-LRP5/6}}} + \frac{[\mathrm{Scl}]}{\mathrm{K}_{\mathrm{Scl-LRP5/6}}}} \tag{26}$$

Eqs. (6) can be used to obtain the concentration of [Scl]:

$$[\mathrm{Scl}] = \frac{\mathrm{P}_{\mathrm{Scl}}}{\tilde{\mathrm{D}}_{\mathrm{Scl}} + \frac{\tilde{\mathrm{D}}_{\mathrm{Scl-LRP5/6}}}{\mathrm{K}_{\mathrm{Scl-LRP5/6}}}\,[\mathrm{LRP5/6}]} \tag{27}$$

where $\tilde{\mathrm{D}}_{\mathrm{Scl}}$ and $\tilde{\mathrm{D}}_{\mathrm{Scl-LRP5/6}}$ are the degradation rates of Scl and the Scl-LRP5/6 complex, respectively, and $\mathrm{K}_{\mathrm{Scl-LRP5/6}}$ is the dissociation constant of the complex. The production of Scl is divided into the endogenous production and the external dosage:

$$\mathrm{P}_{\mathrm{Scl}} = \mathrm{P}_{\mathrm{Scl,b}} + \mathrm{P}_{\mathrm{Scl,d}} \tag{28}$$

and the endogenous production is given by Eq. (11), which in this case reads:

$$\mathrm{P}_{\mathrm{Scl,b}} = \beta_{\mathrm{Scl,Ot}}\, \pi^{\varepsilon}_{\mathrm{rep,Scl}}\, \mathrm{Ot} \left(1 - \frac{[\mathrm{Scl}]}{[\mathrm{Scl}]_{\mathrm{max}}}\right) \tag{29}$$

where $\beta_{\mathrm{Scl,Ot}}$ is the production rate of Scl by osteocytes, $\pi^{\varepsilon}_{\mathrm{rep,Scl}}$ is a Hill-type function that models the downregulating effect of mechanical stimulus on Scl production (see Section 2.6), and $[\mathrm{Scl}]_{\mathrm{max}}$ is the maximum (or saturation) concentration of Scl. Substituting Eqs. (29), (28) and (26) into (27) produces a quadratic equation in [Scl]. After solving this equation, [Scl] can be replaced in (26) to yield [LPR5/6], given that [Wnt] is assumed constant. Finally, using Eqs. (14) and (25), the activator function in the $\mathrm{Ob_p}$ proliferation term can be calculated as:

$$\pi^{\mathrm{Wnt}}_{\mathrm{act,Ob_p}} = \frac{[\mathrm{Wnt-LRP5/6}]}{[\mathrm{LRP5/6}]_{\mathrm{tot}}} = \frac{[\mathrm{Wnt}]\,[\mathrm{LRP5/6}]}{\mathrm{K}_{\mathrm{Wnt-LRP5/6}}\,[\mathrm{LRP5/6}]_{\mathrm{tot}}} \tag{30}$$

2.4. Catabolic role of sclerostin

Wijenayaka et al. [8] showed that elevated Scl concentrations also increase RANKL levels, suggesting an effect on the catabolic RANK-RANKL-OPG pathway. The effect of Scl on RANKL is represented in this model through the factor $\pi^{\mathrm{Scl}}_{\mathrm{act,RANKL}}$, a sigmoid function defined in Eq. (31):

$$\pi^{\mathrm{Scl}}_{\mathrm{act,RANKL}} = 1 + (\alpha_{\mathrm{Scl}} - 1)\,\frac{[\mathrm{Scl}]^{\gamma_{\mathrm{Scl}}}}{[\mathrm{Scl}]^{\gamma_{\mathrm{Scl}}} + \delta_{\mathrm{Scl}}^{\gamma_{\mathrm{Scl}}}} \tag{31}$$

where α_{Scl}, γ_{Scl} and δ_{Scl} are constants. This factor ranges within $\pi^{\mathrm{Scl}}_{\mathrm{act,RANKL}} \in [1, \alpha_{\mathrm{Scl}}]$. The sigmoid nature of this factor ensures that its values exceed 1 only under conditions of elevated Scl concentrations, capturing its effect on RANKL dynamics. This factor affects the production of RANKL by osteocytes (see Eq. (22)).

2.5. Co-regulation of RANKL via PTH and NO concentration

RANKL transcription is upregulated by parathyroid hormone (PTH) and downregulated by nitric oxide (NO). In the model developed by Martin et al. [4] this antagonistic influence was merged into a co-regulatory function capturing both effects.

$$\pi^{\mathrm{PTH,NO}}_{\mathrm{act/rep,RANKL}} = \lambda_{\mathrm{s}}\left(\pi^{\mathrm{PTH}}_{\mathrm{act,RANKL}} + \pi^{\mathrm{NO}}_{\mathrm{rep,RANKL}}\right) + \lambda_{\mathrm{c}}\,\pi^{\mathrm{PTH}}_{\mathrm{act,RANKL}} \cdot \pi^{\mathrm{NO}}_{\mathrm{rep,RANKL}} \tag{32}$$

where λ_{s} and λ_{c} are constants. The activator function accounting for the effect of PTH is:

$$\pi^{\mathrm{PTH}}_{\mathrm{act,RANKL}} = \frac{[\mathrm{PTH}]}{[\mathrm{PTH}] + \mathrm{K}^{\mathrm{PTH}}_{\mathrm{act}}} \tag{33}$$

and the repressor effect on OPG (see Eq. (19)) is accounted for through the function:

$$\pi^{\mathrm{PTH}}_{\mathrm{rep,Ob_a}} = \frac{\mathrm{K}^{\mathrm{PTH}}_{\mathrm{rep}}}{[\mathrm{PTH}] + \mathrm{K}^{\mathrm{PTH}}_{\mathrm{rep}}} \tag{34}$$

being $\mathrm{K}^{\mathrm{PTH}}_{\mathrm{act}}$ and $\mathrm{K}^{\mathrm{PTH}}_{\mathrm{rep}}$ constants. In contrast to previous models in which the concentration of PTH was assumed constant, now it is identified with serum PTH concentration, i.e. $[\mathrm{PTH}]=\mathrm{A}_7$. On the other hand, the factor corresponding to nitric oxide is:

$$\pi^{\mathrm{NO}}_{\mathrm{rep,RANKL}} = \frac{\mathrm{K}^{\mathrm{NO}}_{\mathrm{rep}}}{[\mathrm{NO}] + \mathrm{K}^{\mathrm{NO}}_{\mathrm{rep}}} \tag{35}$$

with $\mathrm{K}^{\mathrm{NO}}_{\mathrm{rep}}$ a constant and the concentration of NO given by:

$$[\mathrm{NO}] = \frac{\mathrm{P}_{\mathrm{NO,d}} + \beta_{\mathrm{NO,Ot}}\,\pi^{\varepsilon}_{\mathrm{act,NO}}\,\mathrm{Ot}}{\tilde{\mathrm{D}}_{\mathrm{NO}} + \frac{\beta_{\mathrm{NO,Ot}}\,\pi^{\varepsilon}_{\mathrm{act,NO}}\,\mathrm{Ot}}{[\mathrm{NO}]_{\max}}} \tag{36}$$

which also comes from Eq. (8) in the absence of ligands. The external dosage of nitric oxide $\mathrm{P}_{\mathrm{NO,d}}$ is set to zero in this study, $\beta_{\mathrm{NO,Ot}}$, $\tilde{\mathrm{D}}_{\mathrm{NO}}$ and $[\mathrm{NO}]_{\max}$ are, respectively, the endogenous production and degradation rate of nitric oxide and its maximum content. The factor $\pi^{\varepsilon}_{\mathrm{act,NO}}$ is the mechanical feedback activator function that accounts for the production of NO by osteocytes. This function and the repressor function affecting the production of sclerostin by osteocytes are defined next.

2.6. Mechanical feedback

Mechanical feedback is a key feature of the bone remodelling process as was first hypothesized by Frost [9]. A low mechanical stimulus leads to bone mass loss, while a high mechanical stimulus can produce bone mass gain if it does not exceed certain limits, what could lead to bone fracture. This idea has traditionally been implemented in phenomenological models, but was first modelled in a BCPM by Pivonka et al. [10] and continued in subsequent models of Pivonka's collaborators, as the present one. In this model, mechanical stimulus dowregulates the production of Scl by osteocytes through the repressor function $\pi^{\varepsilon}_{\mathrm{rep,Scl}}$ (that appears in Eq. (29)), and upregulates the production of NO through the activator function $\pi^{\varepsilon}_{\mathrm{act,NO}}$ (that appears in Eq. (36)). These functions are defined as follows:

$$\pi^{\varepsilon}_{\mathrm{act,NO}} = \rho_{\mathrm{act}} + \frac{(\alpha_{\mathrm{act}} - \rho_{\mathrm{act}})\,(|\varepsilon|_{\mathrm{max}})^{\gamma_{\mathrm{act}}}}{\delta_{\mathrm{act}}^{\gamma_{\mathrm{act}}} + (|\varepsilon|_{\mathrm{max}})^{\gamma_{\mathrm{act}}}} \tag{37}$$

$$\pi^{\varepsilon}_{\mathrm{rep,Scl}} = \alpha_{\mathrm{rep}} - \frac{(\alpha_{\mathrm{rep}} - \rho_{\mathrm{rep}})\,(|\varepsilon|_{\mathrm{max}})^{\gamma_{\mathrm{act}}}}{\delta_{\mathrm{rep}}^{\gamma_{\mathrm{rep}}} + (|\varepsilon|_{\mathrm{max}})^{\gamma_{\mathrm{act}}}} \tag{38}$$

where $\rho_{\sim}$ and $\alpha_{\sim}$ are, respectively, the minimum and maximum anticipated response, ther exponents $\gamma_{\sim}$ are sigmoidicity constants that modify the steepness of the response and $\delta_{\sim}$ is the value of the stimulus that produces the half-maximal response. In the model proposed in [11] the variable used to measure the mechanical stimulus was the maximum principal strain in absolute value, $|\varepsilon|_{\mathrm{max}}$, following the conclusions of [12].

2.7. Damage

Targeted bone remodelling theories hypothesise that one of the major functions of bone remodelling is to remove microcracks from bone matrix, so avoiding an excessive accumulation of the latter, which could result in macroscopic failure [13]. The accumulation of microcracks in a particular volume of material is addressed here using a Continuum Damage Mechanics approach [14]. This theory introduces a damage variable, d, which is linked to the density of microcracks in a volume of material and to the loss of stiffness through Eq. (39). This variable is such that d$\in [0,1]$, with d=0 corresponding to an undamaged state and d=1 to a local fracture or failure situation:

$$\mathbf{C} = (1 - \mathrm{d})\,\mathbf{C}_0 \tag{39}$$

where $\mathbf{C}$ and $\mathbf{C}_0$ are, respectively, the stiffness tensors of damaged and undamaged bone [14]. In the isotropic damage theory, Eq. (39) can be rewritten in terms of the respective Young's moduli, E and E_0, as $\mathrm{E} = (1 - \mathrm{d})\,\mathrm{E}_0$ [15, 16].

A balance of microdamage is considered through the accumulation due to fatigue loading and the removal due to bone remodelling, as osteoclasts resorb the damaged tissue, while the osteoid deposited by osteoblasts is initially intact. The evolution law for damage can be expressed as:

$$\dot{\mathrm{d}} = \dot{\mathrm{d}}_{\mathrm{A}} - \dot{\mathrm{d}}_{\mathrm{R}} \tag{40}$$

where $\dot{\mathrm{d}}_{\mathrm{A}}$ is the rate of damage accumulation by fatigue loading and $\dot{\mathrm{d}}_{\mathrm{R}}$ is the rate of damage removal by bone remodelling. The latter is assessed by assuming that damage is uniformly distributed throughout the representative volume element (RVE). So, the amount of repaired damage is proportional to the damage present in that volume and to the volume of tissue being resorbed, $\dot{\mathrm{V}}_{\mathrm{r}}$, through the fraction that this volume represents within the bone matrix volume:

$$\dot{\mathrm{d}}_{\mathrm{R}} = \mathrm{d}\,\frac{\dot{\mathrm{V}}_{\mathrm{r}}}{\mathrm{V}_{\mathrm{bm}}} = \mathrm{d}\,\frac{\mathrm{k}_{\mathrm{res}} \cdot \mathrm{Oc}_{\mathrm{a}}}{\mathrm{f}_{\mathrm{bm}}} \tag{41}$$

Damage accumulation is evaluated following the procedure described in [17, 18]. This procedure makes use of the results of the experimental fatigue tests performed by Pattin et al. [16], who provided the evolution

of damage with the strain level and the number of cycles. This evolution was mathematically modelled by García-Aznar et al. [19] to yield the following differential equation under tensile stresses:

$$\dot{d}_a = \dot{N} \, \frac{C_1}{C_2 \, \gamma_f} \, (1-d)^{1-\gamma_f} \, \varepsilon_{max}^{\delta_f} \exp\Big(-C_2 \, (1-d)^{\gamma_f} \Big) \tag{42}$$

where $\dot{N}$ is the number of cycles applied per unit time and ε_{max} is the maximum principal strain expressed in $\mu\varepsilon$.[1] The rest of parameters and constants of the model are:

$$\begin{aligned} C_1 &= \frac{e^{C_2} - 1}{K_f([Ca])}; & \delta_f &= 14.1; \\ \gamma_f &= -0.018(\varepsilon_{max} - 4100) + 12; & C_2 &= -20; \end{aligned} \tag{43}$$

where $K_f([Ca])$ is a function of the mineral content which will be defined next. The experimental tests perfomed by Pattin et al. [16] included an estimation of fatigue life, N_f, which was related to the deformation by the following expression:

$$N_f = \frac{K_f}{\varepsilon_{max}^{\delta_f}} \tag{44}$$

where K_f was assumed constant and equal to $1.445 \cdot 10^{53}$ in tension. Martínez-Reina et al. [17] introduced a correction in K_f to consider the degradation of the fatigue properties with the increase in mineral content. A life $N_f = 10^7$ cycles was assigned to the fatigue limit, which is usually assumed to occur for a given fraction of the ultimate tensile strain, ε_u/β, where the parameter β depends on the type of material [20] and $\beta = 2$ was assumed for bone [17] with good results. So, K_f was obtained from Eq. (44) as:

$$K_f([Ca]) = 10^7 \left(\frac{\varepsilon_u([Ca])}{\beta} \right)^{\delta_f} \tag{45}$$

where the ultimate tensile strain depends on the calcium concentration of bone matrix, [Ca], as Currey [21] showed. The following regression was fitted in [17] from the experimental results presented by Currey [21]:

$$\log \varepsilon_u = 31.452 - 11.341 \log [Ca] \tag{46}$$

where ε_u is expressed in $\mu\varepsilon$ and the concentration [Ca] is expressed in mg of calcium per g of bone matrix. This concentration is directly related to the ash fraction, α, which is another measure of mineral content.[2] More precisely, the relation $[Ca] = 398.8\,\alpha$ was assumed, based on the molecular weights of hydroxyapatite and type I collagen [17].

2.8. Upregulation of RANKL expressed by osteocytes due to microstructural damage

As proposed in [7] we have assumed that RANKL expression by osteocytes is upregulated by the presence of microstructural damage in the bone matrix through the factor $\pi_{act,RANKL}^{dam}$, which is defined as a sigmoid function of damage, d:

$$\pi_{act,RANKL}^{dam} = \rho_{dam} + (\alpha_{dam} - \rho_{dam}) \, (1 + \delta_{dam}^{\gamma}) \, \frac{d^{\gamma}}{\delta_{dam}^{\gamma} + d^{\gamma}} \tag{47}$$

where δ_{dam} is a constant that represents the level of damage producing the half response between the minimum value, ρ_{dam} (corresponding to d=0) and the maximum value, α_{dam} (corresponding to d=1), and finally γ is the shape factor of the sigmoid function.

[1] In the damage model proposed by Martínez-Reina et al. [18], cracks were assumed to grow normal to the maximum strain direction and only under tensile strains.

[2] Ash fraction is given by $\alpha = \frac{\rho_m \, v_m}{\rho_m \, v_m + \rho_o \, v_o}$.

2.9. Regulatory role of TGF-β

TGF−β is stored in the bone matrix and released during resorption by osteoclasts. Its concentration is calculated following Pivonka et al. [2]:

$$[\mathrm{TGF}-\beta] = \frac{\alpha_{\mathrm{TGF}-\beta}\, \mathrm{k_{res}}\, \mathrm{Oc_a}}{\tilde{\mathrm{D}}_{\mathrm{TGF}-\beta}} \tag{48}$$

where $\alpha_{\mathrm{TGF}-\beta}$ is the concentration of TGF−β in bone matrix and $\tilde{\mathrm{D}}_{\mathrm{TGF}-\beta}$ is the TGF−β degradation rate. The concentration of TGF−β is used to define the activator/repressor functions in Eqs. (2)-(5) of the main document. These functions control the upregulation of the differentiation of $\mathrm{Ob_u}$ into $\mathrm{Ob_p}$, the upregulation of osteoclast apoptosis and the downregulation of the differentiation of $\mathrm{Ob_p}$ into $\mathrm{Ob_a}$:

$$\pi^{\mathrm{TGF}-\beta}_{\mathrm{act,Ob_u}} = \pi^{\mathrm{TGF}-\beta}_{\mathrm{act,Oc_a}} = \frac{[\mathrm{TGF}-\beta]}{\mathrm{K}^{\mathrm{TGF}-\beta}_{\mathrm{act}} + [\mathrm{TGF}-\beta]} \tag{49}$$

$$\pi^{\mathrm{TGF}-\beta}_{\mathrm{rep,Ob_p}} = \frac{\mathrm{K}^{\mathrm{TGF}-\beta}_{\mathrm{rep}}}{\mathrm{K}^{\mathrm{TGF}-\beta}_{\mathrm{rep}} + [\mathrm{TGF}-\beta]} \tag{50}$$

with $\mathrm{K}^{\mathrm{TGF}-\beta}_{\mathrm{act}}$ and $\mathrm{K}^{\mathrm{TGF}-\beta}_{\mathrm{rep}}$ the activation and repression constants, respectively.

2.10. Proliferation of osteoblast precursors

Recall the differential equation of osteoblast precursors given in the main document.

$$\frac{\mathrm{d\,Ob_p}}{\mathrm{dt}} = \mathrm{D_{Ob_u}} \cdot \mathrm{Ob_u} \cdot \pi^{\mathrm{TGF}-\beta}_{\mathrm{act,Ob_u}} - \mathrm{D_{Ob_p}} \cdot \mathrm{Ob_p} \cdot \pi^{\mathrm{TGF}-\beta}_{\mathrm{rep,Ob_p}} + \mathrm{P_{Ob_p}} \cdot \mathrm{Ob_p} \cdot \pi^{\mathrm{Wnt}}_{\mathrm{act,Ob_p}} \tag{51}$$

We can rewrite this equation as follows.

$$\frac{\mathrm{d\,Ob_p}}{\mathrm{dt}} = \mathcal{D}_{\mathrm{Ob_u}} \cdot \mathrm{Ob_u} - \mathcal{D}_{\mathrm{Ob_p}} \cdot \mathrm{Ob_p} + \mathcal{P}_{\mathrm{Ob_p}} \cdot \mathrm{Ob_p} \tag{52}$$

where the terms in the right-hand side correspond, respectively, to the differentiation of $\mathrm{Ob_u}$ into $\mathrm{Ob_p}$, the differentiation of $\mathrm{Ob_p}$ into $\mathrm{Ob_a}$ and the proliferation of $\mathrm{Ob_p}$ at a rate $\mathcal{P}_{\mathrm{Ob_p}}$ which is determined by $\mathrm{P_{Ob_p}}$ and the Wnt–Scl–LRP5/6 signalling pathway through $\pi^{\mathrm{Wnt}}_{\mathrm{act,Ob_p}}$ (see Eq. (51)). As discussed in Buenzli et al. [22], a necessary condition for the $\mathrm{Ob_p}$ population to stay bounded and to converge to a meaningful steady-state (with finite, positive cell densities) is that:

$$\mathcal{P}_{\mathrm{Ob_p}} - \mathcal{D}_{Ob_p} < 0 \quad \text{as} \quad t \longrightarrow \infty \tag{53}$$

Following Buenzli et al. [22] $\mathrm{P_{Ob_p}}$ was defined considering a saturation factor:

$$\mathrm{P_{Ob_p}} = \begin{cases} \mathrm{P^0_{Ob_p}} \left(1 - \frac{\mathrm{Ob_p}}{\mathrm{Ob_p^{sat}}}\right) & \text{if} \quad \mathrm{Ob_p} < \mathrm{Ob_p^{sat}} \\ 0 & \text{if} \quad \mathrm{Ob_p} \geq \mathrm{Ob_p^{sat}} \end{cases} \tag{54}$$

where $\mathrm{P^0_{Ob_p}}$ is a constant and $\mathrm{Ob_p^{sat}}$ is the maximum concentration of osteoblast precursors above which no proliferation occurs. This saturation and the choice of $\mathrm{P^0_{Ob_p}}$ (see Table 1) ensures that Eq. (53) is fulfilled.

3. Changes introduced in the Peterson and Riggs' model and adjustment of constants

3.1. Ca and PO_4 in blood plasma (central compartment) and EVC+IVF compartment

Contrary to Peterson and Riggs [1], we have distinguished blood plasma from the rest of the extracellular fluid, since the concentrations in both may be different due to Gibbs-Donnan equilibrium [23], and the values more easily found in the literature to validate our results are blood concentrations. The rest of the extracellular fluid, i.e. the extravascular compartment (EVC), and the intracellular fluid (ICF) will be assumed to act as a reservoir and will be grouped in the same compartment (EVC+ICF, see Fig. 1 of the main document) without distinguishing between their concentrations, since only the total amount of species stored in this joint reservoir is relevant for blood plasma equilibrium. The exchange rates between blood plasma and ECV+ICF will be assumed to be large enough to model the rapid osmotic equilibrium through capillaries [24]. The Ca content in blood plasma is then governed by the equation:

$$\frac{\mathrm{dA}_4}{\mathrm{dt}} = \underbrace{\mathrm{J}_{1-4}}_{\substack{\text{Flux}\\\text{from gut}}} + \underbrace{\mathrm{J}_{13-4}}_{\substack{\text{Flux from}\\\text{bone marrow}}} + \underbrace{\mathrm{J}_{8-4}}_{\substack{\text{Flux from}\\\text{EVC+ICF}}} - \underbrace{\mathrm{J}_{4-13}}_{\substack{\text{Flux to}\\\text{bone marrow}}} - \underbrace{\mathrm{J}_{4-\mathrm{u}}}_{\substack{\text{Flux to}\\\text{urine}}} - \underbrace{\mathrm{J}_{4-\mathrm{s}}}_{\substack{\text{Flux to}\\\text{sweat}}} - \underbrace{\mathrm{J}_{4-8}}_{\substack{\text{Flux to}\\\text{EVC+ICF}}} \tag{55}$$

3.2. Adjustment of flux J_{1-4}

Recall the expression of flux J_{1-4}.

$$\mathrm{J}_{1-4} = \mathrm{k}_{1-4} \cdot \left(\underbrace{\mathrm{k}_{\mathrm{pas}} \cdot \mathrm{A}_1 - \varphi_{1-4} \cdot \frac{\mathrm{A}_4}{\mathrm{A}_4^{\mathrm{eq}}}}_{\text{Passive absorption}} + \underbrace{\frac{\mathrm{A}_2}{\mathrm{A}_2^{\mathrm{eq}}} \cdot \mathrm{H}_{1,1-4}}_{\text{Active absorption}} \right) \tag{56}$$

The constants $\alpha_{1,1-4}$, $\delta_{1,1-4}$, and $\gamma_{1,1-4}$ of function $\mathrm{H}_{1,1-4}$ were fitted to reproduce the shape of the curve provided in [25] (Fig. 3 in that work) for the active absorption and assuming a homeostatic situation ($\mathrm{A}_2/\mathrm{A}_2^{\mathrm{eq}} = 1$). The same was done for constants $\mathrm{k}_{\mathrm{pas}}$ and φ_{1-4} by fitting the passive absorption for a homeostatic situation ($\mathrm{A}_4/\mathrm{A}_4^{\mathrm{eq}} = 1$). Adjusting the shape of the curves did not produce a unique fitting, which was obtained by imposing that passive absorption represents a 16% of total absorption (midpoint of the range [8 , 23]%, given in [26]) for the reference value $\mathrm{A}_1 = 1\,\mathrm{mmol}$. Finally, the rate k_{1-4} was adjusted to produce a net flux $\mathrm{J}_{1-4} = 175\,\mathrm{mgCa/day}$ [25].

3.3. Adjustment of functions $\mathrm{H}^{+}_{6,2}$ and $\mathrm{H}^{-}_{6,2}$

Recall the differential equation that governs the activation of Ca transporters.

$$\frac{\mathrm{dA}_2}{\mathrm{dt}} = \underbrace{\mathrm{H}^{+}_{6,2} \cdot (1 - \mathrm{A}_2)}_{\text{Activation of inactive absorbers}} - \underbrace{\mathrm{H}^{-}_{6,2} \cdot \mathrm{A}_2}_{\text{Deactivation of active absorbers}} \tag{57}$$

The stationarity condition ($\mathrm{dA}_2/\mathrm{dt} = 0$) leads to the following condition, given that the constants $\rho_{6,2}$, $\alpha_{6,2}$, $\delta_{6,2}$ and $\gamma_{6,2}$ are the same in the functions $\mathrm{H}^{+}_{6,2}$ and $\mathrm{H}^{-}_{6,2}$:

$$\mathrm{A}_2^{\mathrm{eq}} = \frac{\mathrm{H}^{+}_{6,2}}{\rho_{6,2} + \alpha_{6,2}} \tag{58}$$

We have adjusted the previous constants in terms of the reduction in Ca absorption under vitamin D insufficiency leading to abnormal values of serum calcitriol.

Vitamin D insufficiency does not generally cause malabsorption of Ca because serum calcitriol, which is the major determinant of Ca absorption, is maintained by secondary hyperparathyroidism. However, there must be a calcidiol level below which the serum calcitriol can no longer be sustained and Ca absorption is affected. Need et al. [27] measured Ca absorption, calcidiol and calcitriol levels in patients divided in four groups according to their serum calcidiol concentration: 0-10, 11-20, 21-30 and 31-40 nM. They only found significant differences of calcitriol levels and Ca absorption rates in the 0-10 nM group, concluding that this absorption is directly to calcitriol levels. For this group, both calcitriol levels and Ca absorption were reduced by 36% in average. Therefore, we have reduced flux J_{1-4} (Eq. (70)) in that percentage due to a reduction in

the fraction of Ca transporters, A_2, while A_1 is kept constant and the serum Ca concentration is reduced to 95% ($A_4/A_4(t_0) = 0.95$) of the nominal value, as observed by Need et al. [27] in the deficiency group (0-10 nM). Thus, the ratio of Ca absorption between the deficiency and the normal case is:

$$\frac{J_{1-4}^{def}}{J_{1-4}^{normal}} = 0.6 = \frac{k_{pas} \cdot A_1 - \varphi_{1-4} \cdot 0.95 + \frac{A_2^{def}}{A_2(t_0)} \cdot H_{1,1-4}}{k_{pas} \cdot A_1 - \varphi_{1-4} \cdot 1 + 1 \cdot H_{1,1-4}} \quad \Rightarrow \quad A_2^{def} = 0.284 \tag{59}$$

If equilibrium is forced (Eq. (58)) for that value of $A_2^{def} = 0.284$ under the reduced concentration of serum calcitriol $A_6^{def} = 0.64 \cdot A_6^{normal}$ [27]:

$$\frac{\rho_{6,2}}{\alpha_{6,2}} = \frac{A_2^{def} - \frac{0.64^{\gamma_{6,2}}}{0.64^{\gamma_{6,2}}+1}}{\frac{1}{0.64^{\gamma_{6,2}}+1} - A_2^{def}} \tag{60}$$

This quotient is negative (and therefore meaningless) for $\gamma_{6,2} \leq 2$. We chose a small value of the sigmoidicity ($\gamma_{6,2} = 3.0$) to ensure gradual variations of A_2 with varying calcitriol levels. For that value of $\gamma_{6,2}$ and assuming $\alpha_{6,2} = 1$, Eq. (60) yields $\rho_{6,2} = 0.0986$.

3.4. Production of calcitriol

The content of calcitriol in the blood plasma was governed by the following equation:

$$\frac{dA_6}{dt} = \underbrace{k_{25-OH,D} \cdot A_{10}}_{\text{Production in the kidney}} - \underbrace{k_{6D} \cdot A_6}_{\text{Degradation of calcitriol}} \tag{61}$$

where $k_{6D} = 0.1\,h^{-1}$ is the degradation rate of calcitriol, whose half-life in blood serum is between 5 and 10 hours [28]. The conversion factor $k_{25-OH,D}$ can account for variations in vitamin D intake and is assumed to equal 1 in normal conditions as in Peterson and Riggs' model [1]. Therefore, the stationarity condition of Eq. (61) for the homeostatic case implies:

$$A_{10}^{eq} = \frac{k_{6D} \cdot A_6^{eq}}{k_{25-OH,D}} = 38.1\,\text{pmol} \tag{62}$$

3.5. Production of 1-α-hydroxylase. Functions $H_{7,10}$ *and* $H_{5,10}^{-}$

1-α-hydroxylase is produced in the kidney in a process upregulated by PTH and inhibited by PO_4 plasma levels [29]. These effects were modelled by Peterson and Riggs through the functions $H_{7,10}$ and $H_{5,10}^{-}$, respectively.

$$\frac{dA_{10}}{dt} = \underbrace{k_{10S} \cdot H_{7,10} \cdot H_{5,10}^{-}}_{\text{Production of 1-}\alpha\text{ -hydroxylase}} - \underbrace{k_{10D} \cdot A_{10}}_{\text{Degradation}} \tag{63}$$

Regarding the function $H_{7,10}$, the constant $\gamma_{7,10} = 0.1112$ was taken from Peterson and Riggs [1] as was $\delta_{7,10} = 1.549 \cdot V_{plasma}$ pmol after adjusting for the new plasma volume, while $\alpha_{7,10} = 1.916$ was adjusted so that $H_{7,10} = 1$ for the new homeostatic value $A_7^{eq} = 10.2\,\text{pmol}$. Analogously for the function $H_{5,10}^{-}$, the constants $\alpha_{5,10} = 1.525$ and $\delta_{5,10} = 1.302 \cdot V_{plasma}$ pmol were taken from Peterson and Riggs [1] (the latter adjusted for the new plasma volume), $\gamma_{5,10}$ was changed from 8 to 4 to produce a more gradual sigmoid response and $\rho_{5,10} = 0.273$ was adjusted so that $H_{5,10}^{-} = 1$ for the new homeostatic value $A_5^{eq} = 3.6\,\text{mmol}$. After fixing $H_{7,10} = H_{5,10}^{-} = 1$ for the homeostatic situation, $k_{10D} = 0.05\,h^{-1}$ was taken from Peterson and Riggs [1] and $k_{10S} = 1.905\,\text{pmol} \cdot h^{-1}$ was adjusted to enforce homeostatic equilibrium for the homeostatic value $A_{10}^{eq} = 38.1\,\text{pmol}$.

3.6. Growth and activity of PT glands

The activation of PT glands was governed by the same equation proposed by Peterson and Riggs [1].

$$\frac{dA_{11}}{dt} = \underbrace{(1 - A_{11}) \cdot \alpha_{11} \cdot (0.85 \cdot T^{-}_{6,4} + 0.15)}_{\text{Activation of inactive cells}} - \underbrace{A_{11} \cdot \alpha_{11} \cdot (0.85 \cdot T^{+}_{6,4} + 0.15)}_{\text{Deactivation of active cells}} \tag{64}$$

where the regulatory sigmoid functions $T^{\pm}_{6,4}$ are:

$$T^{\pm}_{6,4} = 1 \pm \tanh\left\{ b_{T6,4} \left[\frac{A_6}{V_{plasma}} - \delta_{T_{6,4}} \cdot \left(\frac{A_4(t_0)}{A_4} \right)^{\gamma_{4,11}} \right] \right\} \tag{65}$$

The constants $\alpha_{11} = 0.01$, $b_{T6,4} = 0.03$ were taken from Peterson and Riggs [1]; $\gamma_{4,11}$ was unspecified by those authors and assumed equal to 0.9 to provide a smooth dependence of PT glands activity on serum Ca level, as this is very tightly controlled. Finally, $\delta_{T6,4} = \frac{A_6^{eq}}{V_{plasma}}$ to enforce that $T^{\pm}_{6,4} = 1$ and hence $\frac{dA_{11}}{dt} = 0$ in homeostatic conditions ($A_{11}^{eq} = 0.5$).

The size of PT glands was governed by the following equation in Peterson and Riggs' model:

$$\frac{dA_{12}}{dt} = \underbrace{k_{12} \cdot H^{-}_{6,12}}_{\text{Growth}} - \underbrace{k_{12} \cdot A_{12}}_{\text{Reduction}} \tag{66}$$

Here we have removed the function $H^{-}_{6,12}$, since the effect of calcitriol on PTH production was already considered through the functions $T^{\pm}_{6,4}$. We have also added a term k_{HPT} to model primary hyperparathyroidism (pHPT), thus resulting in:

$$\frac{dA_{12}}{dt} = \underbrace{k_{12}}_{\text{Growth}} - \underbrace{k_{12} \cdot A_{12}}_{\text{Reduction}} + \underbrace{k_{HPT}}_{\text{pHPT}} \tag{67}$$

Without pHPT ($k_{HPT} = 0$), the equilibrium of Eq. (66) ensures $A_{12}^{eq} = 1$, i.e. normal size of PT glands with no influence on the production of PTH. In case of pHPT $A_{12}^{eq} = \frac{k_{12}+k_{HPT}}{k_{12}}$. Thus, the constants k_{12} and k_{HPT} control the equilibrium size of the PT glands and therefore the PTH production in pHPT and were fitted using clinical data [30, 31].

3.7. Fluxes of Ca and PO_4 from or into blood serum

The equilibrium equation of serum Ca was:

$$\frac{dA_4}{dt} = \underbrace{J_{1-4}}_{\substack{\text{Flux}\\ \text{from gut}}} + \underbrace{J_{13-4}}_{\substack{\text{Flux from}\\ \text{bone marrow}}} + \underbrace{J_{8-4}}_{\substack{\text{Flux from}\\ \text{EVC+ICF}}} - \underbrace{J_{4-13}}_{\substack{\text{Flux to}\\ \text{bone marrow}}} - \underbrace{J_{4-u}}_{\substack{\text{Flux to}\\ \text{urine}}} - \underbrace{J_{4-s}}_{\substack{\text{Flux to}\\ \text{sweat}}} - \underbrace{J_{4-8}}_{\substack{\text{Flux to}\\ \text{EVC+ICF}}} \tag{68}$$

and the equation corresponding to serum PO_4 was:

$$\frac{dA_5}{dt} = \underbrace{J_{14-5}}_{\substack{\text{Flux from}\\ \text{bone marrow}}} - \underbrace{J_{5-14}}_{\substack{\text{Flux to}\\ \text{bone marrow}}} - \underbrace{J_{5-u}}_{\substack{\text{Flux to}\\ \text{urine}}} + \underbrace{J_{3-5}}_{\substack{\text{Flux}\\ \text{from gut}}} - \underbrace{J_{5-9}}_{\substack{\text{Flux to}\\ \text{EVC+ICF}}} + \underbrace{J_{9-5}}_{\substack{\text{Flux from}\\ \text{EVC+ICF}}} \tag{69}$$

3.7.1. Flux of Ca from gut into blood serum

The flux of Ca into the blood plasma is given by:

$$J_{1-4} = k_{1-4} \cdot \left(\underbrace{k_{pas} \cdot A_1 - \varphi_{1-4} \cdot \frac{A_4}{A_4^{eq}}}_{\text{Passive absorption}} + \underbrace{\frac{A_2}{A_2^{eq}} \cdot H_{1,1-4}}_{\text{Active absorption}} \right) \tag{70}$$

where the first two terms account for non-saturable passive (paracellular) absorption and the third term corresponds to the active saturable absorption (transcellular). The first term considers the unidirectional

flux of Ca from the intestinal lumen to the blood as a linear function (k_{pas}) of the luminal Ca concentration (or equivalently of A_1).[3] The second term (φ_{1-4}) models the unidirectional back-flux of Ca from blood into the gut, which is largely determined by the level of extracellular Ca (A_4), compared to the homeostatic (and initial) value (A_4^{eq}) and tends to be relatively insensitive to changes in dietary Ca intake [25].

The third term (active absorption) is saturable and dependent on the luminal Ca concentration through the function $H_{1,1-4}$. It also accounts for the bioavailability of Ca transporters in intestinal enterocytes, i.e., the binding protein calbindin D_{9k} (CaBP), through the factor A_2/A_2^{eq}. The inclusion of this factor agrees with Bronner [32] who reported a linear dependency between CaBP concentration and transcellular Ca absorption. We used the fraction of active transporters as a variable in our model instead of the CaBP concentration itself, following Peterson and Riggs [1] who also normalised that fraction to the initial and homeostatic value ($A_2^{eq} = 0.5$).

3.7.2. Flux of Ca from blood serum into sweat

This flux is given by the following expression:

$$J_{4-s} = k_{4-s} \cdot A_4 \tag{71}$$

where k_{4-s} was adjusted so that the normal flux of Ca to sweat under homeostatic conditions (A_4=7.35 mmol) is $J_{4-s} = 1.5$ mmol/day [33], yielding $k_{4-s} = 8.5 \cdot 10^{-3}\,h^{-1}$.

3.7.3. Flux of Ca from blood serum into urine

The flux of Ca to urine defined by Peterson and Riggs [1] was reformulated to solve what, in our opinion, are some inconsistencies in the original expression. This expression was:

$$J_{4-u} = (2 - H_{6,4-u}) \cdot \left(\underbrace{k_{4-u} \cdot GFR \cdot \frac{A_4}{V_{plasma}}}_{\text{Filtration}} - \underbrace{H_{4,4-u} \cdot H_{7,4-u}}_{\text{Reabsorption}} \right) \tag{72}$$

and the one proposed here is:

$$J_{4-u} = H^{-}_{6,4-u} \cdot GFR \cdot \left(\underbrace{k_{4-u,filt} \cdot \frac{A_4}{V_{plasma}}}_{\text{Filtration}} - \underbrace{k_{4-u,reabs}(A_4) \cdot H^{+}_{7,4-u}}_{\text{Reabsorption}} \right) \tag{73}$$

First, it must be noted that the factor $(2 - H_{6,4-u})$ (if $\alpha_{6,4-u} = 2$ as used by [1]) is equivalent to $H^{-}_{6,4-u}$ with $\rho_{6,4-u} = 0$ and thus, this is not a relevant change. The most relevant one is that GFR only multiplied the filtration term in the expression proposed by Peterson and Riggs [1] (Eq. (72)) and now it multiplies also the reabsorption term. This change was made based on three premises:

1. To be in accordance with the excretion of PO_4 provided by Peterson and Riggs [1] where GFR also multiplies both terms.
2. Because blood is first filtrated in the glomerulus and then some constituents are passed into the tubules, where a fraction of them are reabsorbed and not excreted into urine. Hence, the amount of Ca reabsorbed in the proximal tubule and loop of Henle must be proportional to the filtrated amount, i.e. to GFR, in contrast to the original expression, where the reabsorbed amount was not proportional to GFR.

[3]It is plausible to work with quantities (A_1) instead of concentrations, without needing to know the distribution volume of the intestinal lumen, which can be considered in the constant k_{pas}, which is adjusted according to other reasons and independently of that distribution volume. For this reason, A_1 can be used as a measure of luminal Ca concentration.

3. Chronic kidney disease (CKD) cannot be reliably modelled using the original expression. By doing so, CKD resulted in a declining filtration capacity (GFR), which yielded $J_{4-u} = 0$ using the original model and when the threshold (reabsortion term) was roughly constant.[4] This would inevitably lead to severe hypercalcaemia, in contradiction with clinical results. In contrast, Eq. (73) leads to a decrease in J_{4-u} with a decreasing GFR, though never reaching a null flux. Fig. 1 compares the current model, implementing Eq. (73) with a model that incorporated Eq. (72) instead. It can be seen that the latter produces a remarkable decline in the Ca flux to urine during the development of the disease, which becomes zero after approximately 2 years. As a consequence, severe hypercalcemia, which is likely incompatible with life, is obtained.

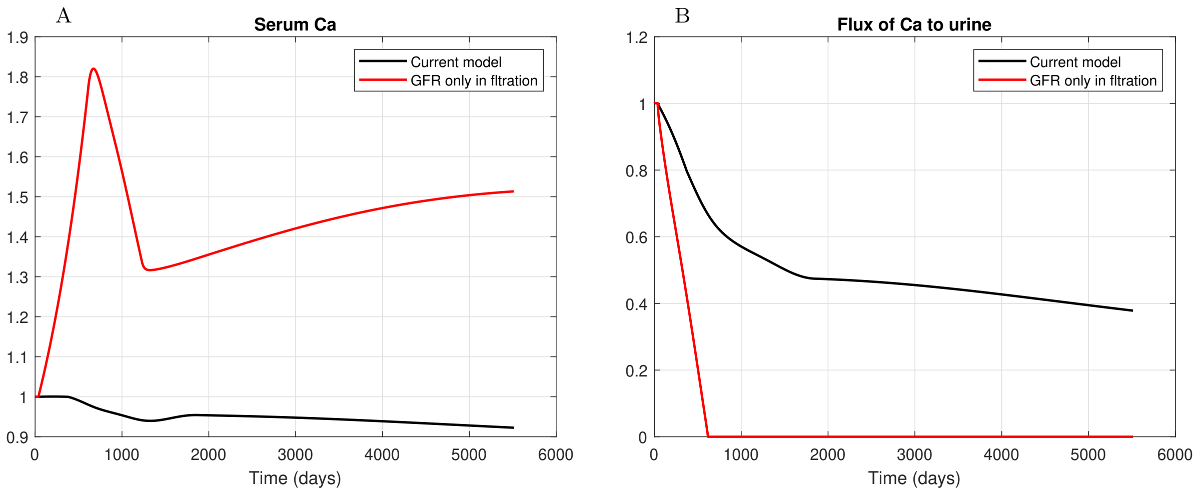

Figure 1: Comparison of the results of the current model, implementing Eq. (73) for the Ca flux to urine (black) with the results of a model that incorporated Eq. (72) instead (red).

The reabsorption term also differs from that originally proposed by Peterson and Riggs [1], who considered a non-linear function of serum Ca, $H_{4,4-u}$, which was almost constant except for very low serum Ca levels, when it decreased though very slightly. Therefore, its influence was very limited and has been replaced by a constant, $k_{4-u,reabs}$. Parfitt [29] highlighted the importance of PTH on reabsorbed Ca and this dependence has been retained, though the original function $H_{7,4-u}$ has been replaced with $H^{+}_{7,4-u}$, by introducing a new constant $\rho_{7,4-u} \neq 0$, as will be commented on later.

The values of $\alpha_{6,4-u} = 2$ and $\gamma_{6,4-u} = 8$ were taken from [1], and $\delta_{6,4-u}$ was chosen following those authors, i.e. equal to the homeostatic value of calcitriol, A_6^{eq}, which has now changed. This way, using $\alpha_{6,4-u} = 2$ enforces that the factor $(2 - H_{6,4-u})$ is equal to 1 in the homeostatic situation and decreases from that value with increasing serum calcitriol, thus diminishing the amount of Ca excreted by urine.

The constant $k_{4-u,filt}$ was adjusted so that the amount of Ca filtrated in the kidney was 10000 mg/day [25] under homeostatic conditions ($(2 - H_{6,4-u}) = 1$ and $H^{+}_{7,4-u} = 1$) and a normal GFR = 6 L/h.

The amount of Ca reabsorbed in the kidney is around 9825 mg/day according to Strewler [25], which would yield $J_{4-u} = 175$ mg/day. This flux equals the value of J_{1-4} provided by the same author and used here to adjust intestinal absorption, and this would ensure equilibrium, but only if J_{4-s} did not exist. Indeed, in homeostatic conditions $J_{4-8} = J_{8-4}$ and $J_{4-13} = J_{13-4}$ as will be seen later, and the equilibrium of Eq. (68) is guaranteed only if $J_{1-4} = J_{4-u} + J_{4-s}$. Strewler [25] did not consider J_{4-s}, and to restore equilibrium, we have assumed $J_{4-s} = 60$ mg/day [33] included in the 175 mg/day that are excreted according to Strewler [25]. Thus, the amount excreted via urine would be 115 mg/day in homeostatic conditions and, if 10000

[4]It is important to note that fluxes are unidirectional, i.e. negative fluxes are not allowed, and if this occurs, they are set equal to zero.

mg/day are filtrated, the reabsorbed amount would be 9885 mg/day, closer to 99% of the filtrated amount, as stated by Kim [34]. Therefore, the constant $k_{4-u,reabs} = 1.716\,mM$ was adjusted by enforcing that the amount of Ca reabsorbed was 9885 mg/day under homeostatic conditions ($(2 - H_{6,4-u}) = 1$ and $H^{+}_{7,4-u} = 1$) and a normal GFR = 6 L/h.

The constants of function $H^{+}_{7,4-u}$ were chosen as follows. $\alpha_{7,4-u} = 1.06$ was taken from [1], implying that a maximum increase of 6% in Ca reabsorption can be achieved by increasing serum PTH levels. The constant $\gamma_{7,4-u}$ was not specified by [1] and a value of 1 has been assumed to produce a gradual increase in Ca reabsorption. The constant $\rho_{7,4-u} = 0.9$ has been introduced in the new model to establish a minimum Ca reabsorption when serum PTH is reduced.[5] Finally, $\delta_{7,4-u} = 6.12\,pmol$ was adjusted to produce $H^{+}_{7,4-u} = 1$ for the homeostatic value $A_7^{eq} = 10.2\,pmol$.

3.7.4. Flux of PO_4 from blood serum into urine J_{5-u}

We have used the same expression proposed by Peterson and Riggs [1] for this flux:

$$J_{5-u} = k_{5-u} \cdot GFR \cdot \left(\underbrace{\frac{A_5}{V_{plasma}}}_{\text{Filtration}} - \underbrace{\varphi_{5-u}}_{\text{Reabsorption}} \right) \tag{74}$$

The amounts of P filtrated and reabsorbed in the kidneys are 7000 mg/day and 6100 mg/day, respectively [35].[6] These values allow us to estimate the constants $k_{5-u} = 1.307$ and $\varphi_{5-u} = 1.046\,mM$ for the homeostatic situation $A_5 = A_5^{eq} = 3.6\,mmol$ and a normal glomerular filtration rate, GFR=6 L/h.

3.7.5. Exchange of Ca and PO_4 between blood serum and EVC+ICF compartment

We must note that the current formulation of Ca/P exchange between blood serum, bone marrow and bone matrix is substantially different to the model presented by Peterson and Riggs [1]. These authors termed the Ca contained in the intracellular compartment as “immediately exchangeable Ca” and the Ca contained in the bone compartment as “non-immediately exchangeable Ca”. They did the same for PO_4. Thus, they established the flux of both ions from blood serum to the intracellular compartment and from here to the bone compartment. We believe that it is more reasonable to define the intracellular compartment as a separate “non-bone” compartment (thus following Evenepoel et al. [33]) rather than as an intermediate compartment between blood serum and bone matrix. This intermediate compartment should correspond to bone marrow, not considered in [1] and added by us to the model (see Fig. 1 of the main document).

The fluxes of Ca between blood serum and EVC+ICF compartment were modelled as:

$$J_{4-8} = k_{4-8} \cdot A_4 \tag{75}$$

$$J_{8-4} = k_{8-4} \cdot A_8 \tag{76}$$

The flux rates k_{4-8} and k_{8-4} were adjusted to produce an exchange rate $J_{4-8} = J_{8-4} = 25\,mmol/day$ for the homeostatic values $A_4^{eq} = 7.35\,mmol$ and $A_8^{eq} = 127\,mmol$. This procedure yielded $k_{4-8} = 0.1417\,h^{-1}$ and $k_{8-4} = 0.0082\,h^{-1}$.

The fluxes of PO_4 between plasma and the EVC+IVF compartment were:

$$J_{5-9} = k_{5-9} \cdot A_5 \tag{77}$$

$$J_{9-5} = k_{9-5} \cdot A_9 \tag{78}$$

The flux rates k_{5-9} and k_{9-5} were adjusted to enforce the homeostatic equilibrium ($J_{5-9} = J_{9-5}$) for the homeostatic values of $A_5^{eq} = 3.6\,mmol$ and $A_9^{eq} = 1966\,mmol$, i.e.

[5] Note that $H_{7,4-u}$ in Eq. (72) implies that $\rho_{7,4-u} = 0$ and this means that renal Ca reabsorption could be virtually eliminated if PTH levels drop significantly.

[6] It must be noted that the amounts mentioned in [35] correspond to phosphorus while our variables measure the amounts of phosphate; therefore, they are converted to mol before being assigned to PO4 amounts.

$$\frac{k_{5-9}}{k_{9-5}} = \frac{A_9^{eq}}{A_5^{eq}} = 546 \tag{79}$$

No data were found in the literature about these fluxes and therefore, we arbitrarily chose $k_{5-9} = 0.1417\,h^{-1}$, equal to k_{4-8}. This results in a very low value of $k_{9-5} = 2.6 \cdot 10^{-4}\,h^{-1}$, which implies that little PO_4 is exchanged to the blood serum from the EVC+IVF compartment, since it is mostly present as part of cell membranes, ATP and proteins, i.e. not directly exchangeable.

3.7.6. Exchange of Ca and PO_4 between blood serum and bone marrow

The fluxes of Ca between these compartments were modelled as:

$$J_{4-13} = k_{4-13} \cdot A_4 \cdot \frac{p}{p_0} \tag{80}$$

$$J_{13-4} = k_{13-4} \cdot A_{13} \cdot \frac{p_0}{p} \tag{81}$$

where p and p_0 are the current and initial (or reference) porosity, as explained in tye main document. Exchange of Ca between bone and plasma is $J_{4-13} = J_{13-4} = 12.5\,\text{mmol/day}$ [25]. This condition was used to adjust k_{4-13} and k_{13-4} in order to fulfil that condition for a homeostatic situation given by $A_4^{eq} = 7.35\,\text{mmol}$ $A_{13}^{eq} = 3.65\,\text{mmol}$ and $p = p_0$. This yielded $k_{4-13} = 7.086 \cdot 10^{-2}\,h^{-1}$ and $k_{13-4} = 0.1427\,h^{-1}$.

The exchange of PO_4 between blood plasma and bone marrow was defined analogously to that of Ca:

$$J_{5-14} = k_{5-14} \cdot A_5 \cdot \frac{p}{p_0} \tag{82}$$

$$J_{14-5} = k_{14-5} \cdot A_{14} \cdot \frac{p_0}{p} \tag{83}$$

and the rate constants were adjusted analogously from the homeostatic exchange rate $J_{5-14} = J_{14-5} = 200\,\text{mg P/day}$ [35], yielding $k_{5-14} = 7.467 \cdot 10^{-2}\,h^{-1}$ and $k_{14-5} = 0.159\,h^{-1}$.

3.8. Amount of Ca in the different compartments

Ca accounts for 1-2 % of adult human body weight [36]. If we assume a body weight of 70 kg, this gives us 1050 g of Ca in the body. Over 99 % of total body Ca is found in the teeth and bones [36], which leaves around 10 g (250 mmol) for the rest. 98 % of total Ca is stored in the skeleton [37], which makes 1029 g ($A_{15} = 25725$ mmol). The amount of Ca in the plasma is calculated from the average concentration, 2.45 mM [37], and the volume of blood serum (3 L), which yields $A_4 = 7.35$ mmol.

For the bone marrow compartment we have assumed a homeostatic value of Ca content A_{13}=3.65 mmol, based on the following data. The amount of bone marrow in a healthy adult male is approximately 3.65 kg [38]. The density of bone marrow is between 0.9 (fatty marrow [39]) and 1.06 g/ml (red bone marrow [40]) and we have assumed 1 g/ml, which provides 3.65 L of bone marrow. Finally, the concentration of Ca in the bone marrow is within 0.5 and 1.6 mM [41] and we have assumed a mean value of 1 mM. This would leave 239 mmol for the EVC+IVF compartment and the rest of tissues, mainly muscles, from which 27 mmol would be stored in the EVC, if we assumed the same concentration of Ca in the EVC than in blood serum (2.45 mM) and a volume $V_{EVC} = V_{ECF} - V_{plasma} = 11$ L, where the total volume of extracellular fluid $V_{ECF} = 14$ L is taken from [1]. Finally, the amount of Ca stored in the ICF is 100 mmol, according to both Peterson and Riggs [1] and Evenepoel et al. [33], and therefore, A_8=127 mmol.

3.9. Amount of PO_4 in the different compartments

The homeostatic content of PO_4 stored in bone matrix was calculated from the Ca content, A_{15}, and assuming the corresponding stoichiometric ratio (1:0.464) of hydroxyapatite as done in Peterson and Riggs [1], thus yielding A_{16}=11936 mmol.

The homeostatic content of PO_4 in EVC+IVF was calculated from A_{16} and following Qadeer and Bashir [42], who stated that 85% of phosphate is contained in bones (A_{16}), 1% in the extracellular fluid and 14% is distributed in other tissues where it is an important component of cell membranes, nucleic acids, ATP and intracellular signaling proteins. This 14% has been assigned to the EVC+IVF (A_9=1966 mmol), though only

a small fraction of it is truly exchangeable with blood serum, and therefore the constant k_{9-5} was chosen very small to account for this marginal exchange.

Wagner [35] provides the range 0.9 - 1.5 mM for the concentration of PO_4 in blood serum. We have chosen the mid point 1.2 mM, which is in agreement with the range provided by Qadeer and Bashir [42] for phosphorus (2.5 - 4.5 mg/dL). With the assumed volume of blood serum (3L), that concentration yields $A_5 = 3.6$ mmol. The homeostatic concentration of PO_4 in bone matrix is assumed to be related to the Ca concentration through the hydroxyapatite stoichiometric ratio as assumed by Peterson and Riggs [1] in both, the immediately exchangeable and non-immediately exchangeable compartments, i.e. $A_{14} = 0.464 \cdot A_{13} = 1.69$ mmol.

4. Factor to control systemic-local transition

We have analysed a RVE of $f_{bm}^{aver} = 44\%$ which we assume to represent the entire skeleton. That value comes from assuming that 80% of the skeleton is cortical bone and the rest is trabecular bone [43] and assuming the typical values $f_{bm}^{cort} = 95\%$ [44] and $f_{bm}^{trab} = 14\%$ [45]. Then, the average bone volume fraction in the skeleton would be:

$$f_{bm}^{aver} = \frac{V_b^{skel}}{V^{skel}} = \frac{V_b^{skel}}{V^{cort} + V^{trab}} = \frac{1}{\frac{V_b^{cort}}{f_{bm}^{cort} \cdot V_b^{skel}} + \frac{V_b^{trab}}{f_{bm}^{cort} \cdot V_b^{skel}}} = \frac{1}{\frac{0.8}{0.95} + \frac{0.2}{0.14}} = 0.44 \tag{84}$$

The transition from local to systemic is governed by the factor f_{SL}. Thus, the bone turnover rate (BToR) is given by:

$$\mathrm{BToR} = \frac{\dot{V}_r}{V_b} f_{SL} = \frac{Oc_a \cdot k_{res} \cdot V_{RVE}}{0.44 \cdot V_{RVE}} f_{SL} \tag{85}$$

Replacing the homeostatic value obtained in our simulations, $Oc_a = 6.92 \cdot 10^{-5}$ pM, and the constant $k_{res} = 200\,\%\,\mathrm{day}^{-1}\,\mathrm{pM}^{-1}$, the amount of tissue resorbed in 1 year would be 5.05% if $f_{SL} = 1$. This means that ~ 8.7 years would be needed to renew the total 44% if $f_{SL} = 1$. Langdahl et al. [43] stated that the adult skeleton is renewed every 10 years, while Jee [46] stated that 3% of the cortical bone and 26% of the trabecular bone are renewed yearly. With the respective proportions mentioned above (80% and 20%), 7.6% of the skeleton would be renewed every year in the latter case, and ~ 13.2 years would be required to renew the entire skeleton. Therefore, f_{SL} must be between 0.66 and 0.87 to be in accordance with these data. The value $f_{SL} = 0.75$ was adopted.

Next, we recalibrated $H_{6,4-u}^{-}$ (in particular $\rho_{6,4-u}$) to adjust serum Ca in cases where calcitriol rises, e.g. in pHPT (*Exp4*), when $\rho_{6,4-u}$ does play a role. We kept the other parameters in $H_{6,4-u}^{-}$ approximately constant, as they play a major role when calcitriol decreases, i.e. vitamin D deficiency (*Exp2*) and CKD (*Exp3*). With this readjustment, our simulations predicted a small increase in serum Ca levels, which are consistent with clinical studies [30, 31] that only found Ca levels slightly above the normal range (maximum 6% above the upper limit). In contrast, pHPT leads to hypophosphatemia [47], not observed in our simulations, which predicted a negligible increase in PO_4 levels (see Fig. 6A of the main document).

Once the model was calibrated, we analysed dietary changes in healthy subjects (*Exp5*) and the results are summarised in Fig. 7 of the main document. This figure represents the values of a number of variables reached after 5 years of dietary change, normalised with respect to the value obtained for a normal diet. It can be seen that a Ca intake above normal has a negligible effect on the results and vitamin D supplementation only a minor effect. In contrast, deficiencies of both could have a strong and negative impact on bone and serum Ca levels. PO_4 intake has the opposite effect, as an increased intake seems detrimental for bone, while a reduction has a slight though beneficial effect.

5. Results

CKD was analysed in *Exp3* in the main document by studying the serum levels of Ca, PO_4, calcitriol and PTH in patients with different GFR. Only the values of these biomarkers at the end of the simulation (comprising 5 years of progressive GFR decline followed by 10 years) were presented. Now, in Fig. 2 we

present the temporal evolution of those biomarkers in a group of patients, Group 1 of [48] with severe CKD. We can observe that biomarkers change notably during the development of the disease (5 years) to stabilise afterward, with the exception of PTH, which continues to increase although at a slower rate.

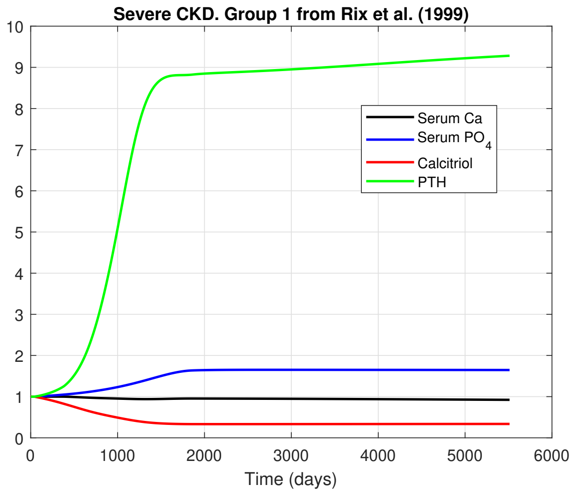

Figure 2: Temporal evolution of serum Ca, serum PO_4, calcitriol and PTH (normalised to the initial value) in a case of severe CKD (Group 1 of [48], see Table 2 of the main document).

Fig. 5 shows some results of *Exp5* (dietary changes in healthy subjects) not shown in the main document. There we presented the values of apparent density, serum Ca, calcitriol, and PTH after 5 years of dietary changes, i.e. for different values of Ca intake (D_1), vitamin D supplementation or deficiency through $k_{25-OH,D}$, and PO_4 intake (D_3). Now, we present two analogous plots for 10 and 15 years after the dietary changes in Figs. 3 and 4, respectively. We also present the temporal evolution of apparent density, f_{bm}, the population of active osteoclasts, serum Ca, calcitriol and PTH, normalised to their initial values and for different dietary changes (Fig. 5). We can observe that dietary changes affect almost immediately the concentrations of serum calcitriol and PTH, and the population of active osteoclasts, which remain almost constant if no further dietary changes occur. Serum Ca undergoes negligible variations with increased Ca intake, vitamin D supplementation and PO_4 reduction, and a slow but steady decrease with Ca and vitamin D deficiency and PO_4 increased intake. Bone volume fraction, f_{bm}, and apparent density change more gradually and steadily over time, increasing with PO_4 reduction and vitamin D supplementation and decreasing with a Ca-deficient diet, vitamin D deficiency and PO_4-rich diet. No noticeable changes were observed for Ca-rich diets.

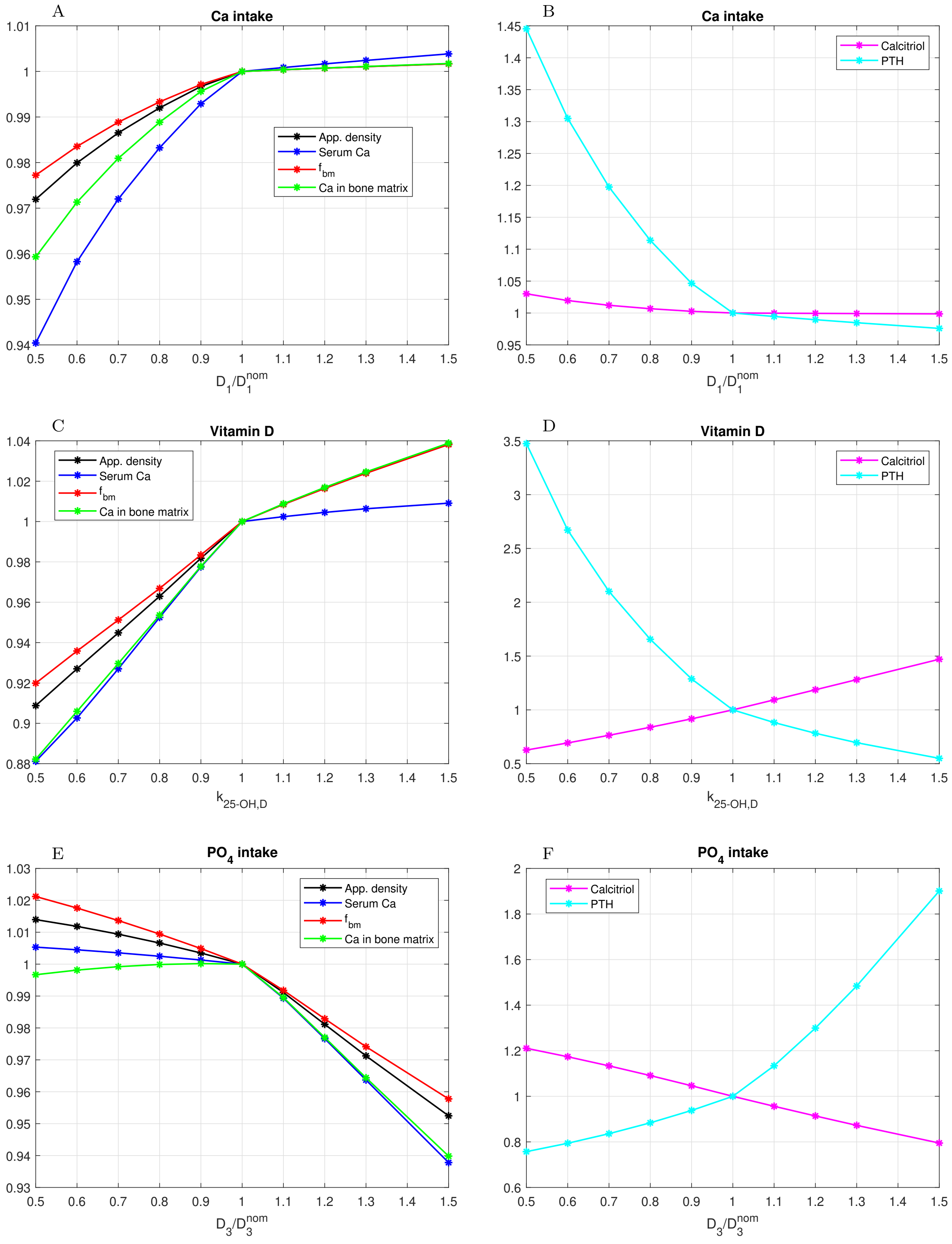

Figure 3: Results of *Exp5* – Influence of dietary changes on healthy individuals. Ca intake (D_1), the constant $k_{25-OH,D}$, related to calcidiol availability, and PO_4 intake (D_3) were changed proportionally to their nominal values. The values of apparent density, serum Ca, f_{bm}, Ca accumulated in bone matrix, calcitriol and PTH after 10 years of simulation are presented.

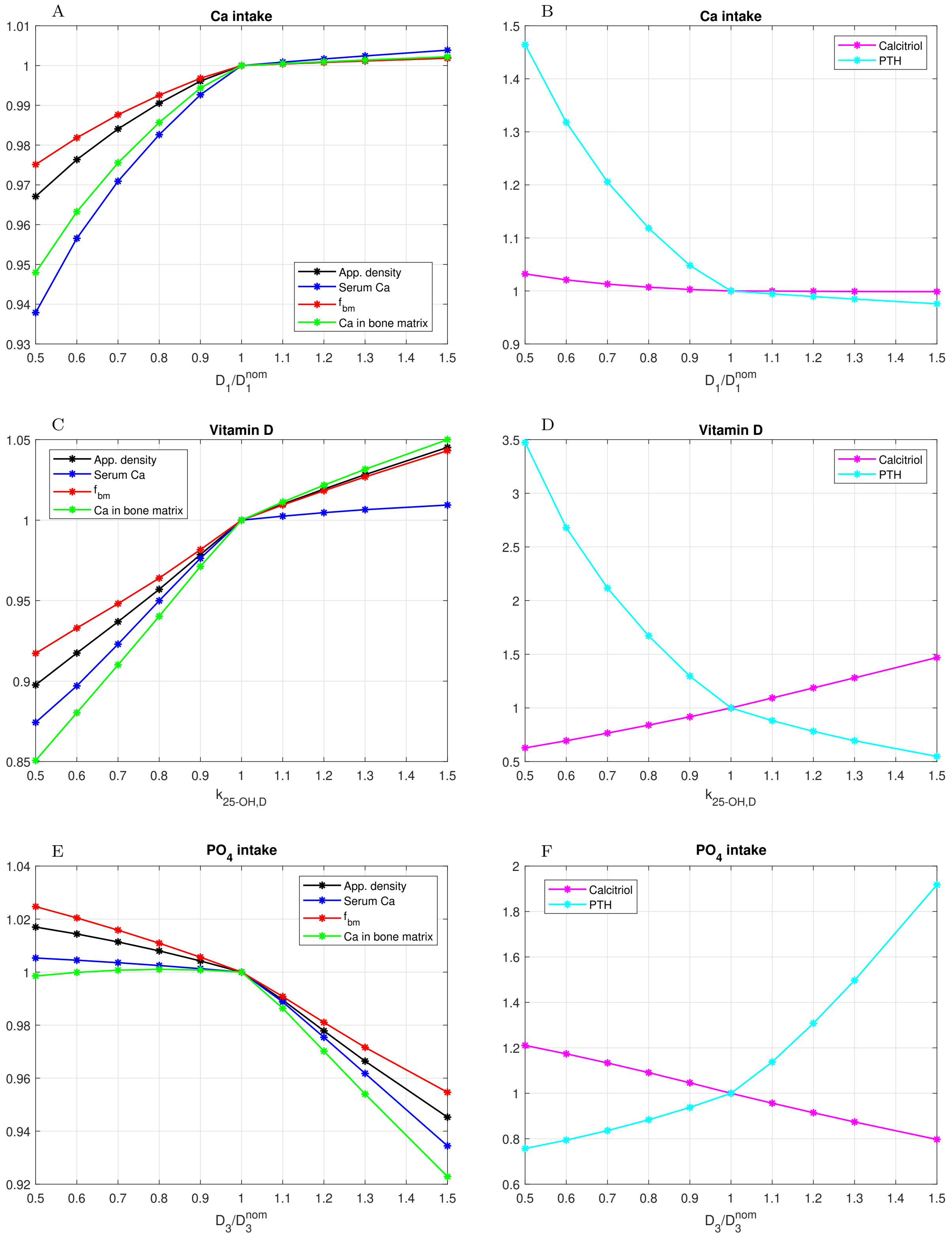

Figure 4: Results of *Exp5* – Influence of dietary changes on healthy individuals. Ca intake (D_1), the constant $k_{25-OH,D}$, related to calcidiol availability, and PO_4 intake (D_3) were changed proportionally to their nominal values. The values of apparent density, serum Ca, f_{bm}, Ca accumulated in bone matrix, calcitriol and PTH after 15 years of simulation are presented.

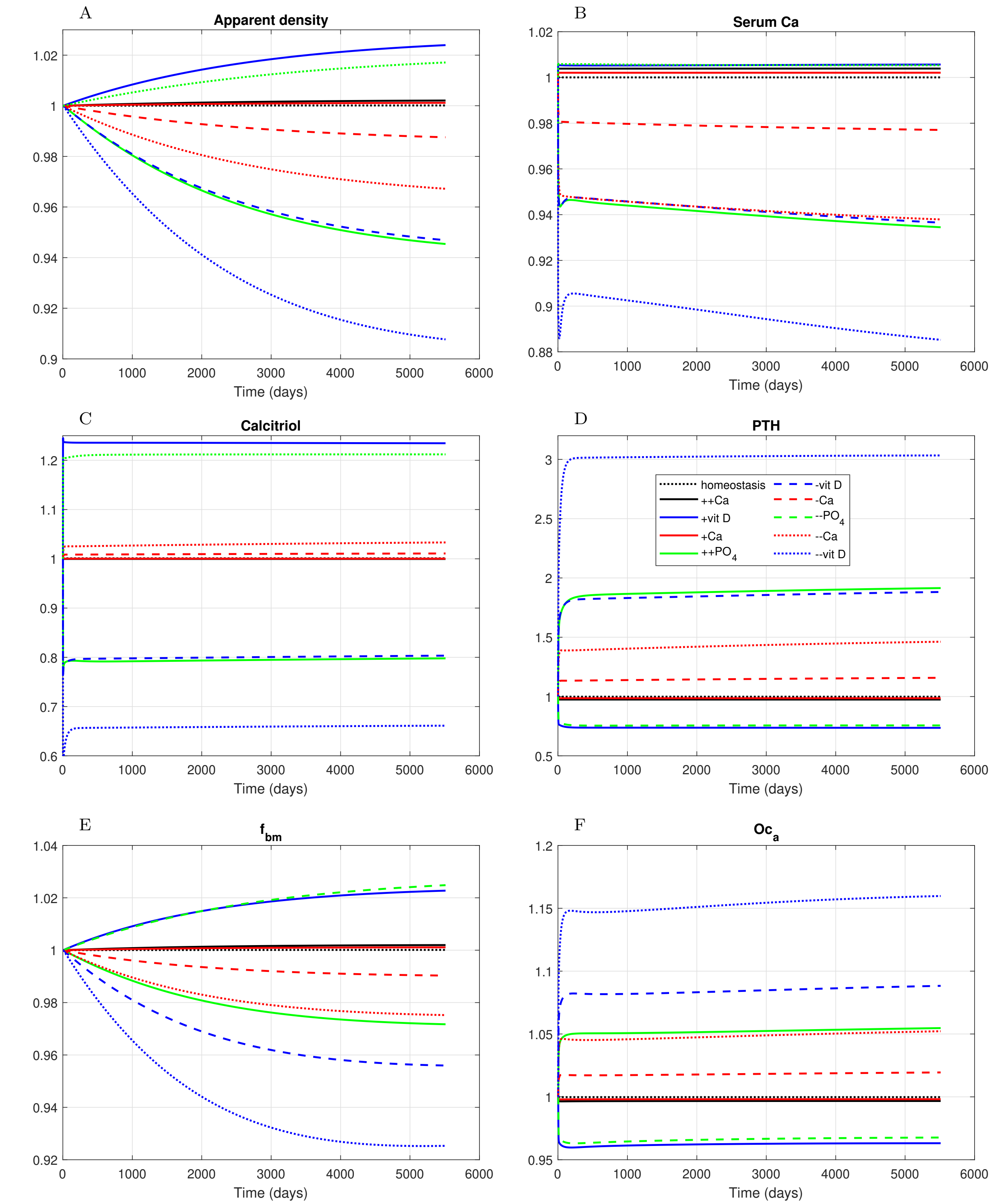

Figure 5: Results of *Exp5* – of dietary changes in healthy subjects. From left to right, temporal evolution of normalised apparent density, serum Ca, calcitriol and PTH in a healthy individual under changes in Ca, PO $_4$ and vitamin D intake: 1) 50% increase in Ca intake (++Ca, solid black); 2) vitamin D supplementation, $k_{25-OH,D} = 1.25$ (+vit D solid blue); 3) 25% increase in Ca intake (++Ca, solid red); 4) 50% increase in PO $_4$ intake (++PO_4, solid green); 5) vitamin D insufficiency, $k_{25-OH,D} = 0.75$ (-vit D, dashed blue); 6) 25% decrease in Ca intake (-Ca, dashed red); 7) 50% decrease in PO $_4$ intake (–PO_4, dashed green); 8) 50% decrease in Ca intake (–Ca, dotted red); 9) vitamin D deficiency, $k_{25-OH,D} = 0.55$ (–vit D, dotted blue). This case corresponds to *Exp2*.

6. Constants of the model

The model constants are provided in the table 1, except for those related to the microstructural damage model, which were given in the Section 2.7 of this document.

Constant	Value	Units	Reference
Cell constants: differentiation, proliferation, apoptosis, activity			
Ob_u	0.01	pM	[49]
Oc_u	0.01	pM	[49]
D_{Ob_u}	0.01	day^{-1}	[49]
D_{Ob_p}	0.0705	day^{-1}	[49]
$P^0_{Ob_p}$	3.47	day^{-1}	[49]
Ob_p^{sat}	0.005	pM	[49]
D_{Oc_u}	0.0388	day^{-1}	[49]
D_{Oc_p}	0.1216	day^{-1}	[49]
Δ_{Ob_a}	0.3502	day^{-1}	[49]
A_{Oc_a}	4.5	day^{-1}	[49]
η	$4.143 \cdot 10^{-4}$	pM / %	[49]
k_{res}	200	% day^{-1} pM^{-1}	[49]
k_{form}	40	% day^{-1} pM^{-1}	[49]
RANK-RANKL-OPG signalling pathway			
$\tilde{D}_{OPG}$	0.35	day^{-1}	[49]
$\tilde{D}_{RANKL}$	0.4053	day^{-1}	[49]
$\tilde{D}_{OPG-RANKL}$	559.67	day^{-1}	[49]
$\tilde{D}_{RANK-RANKL}$	10.132	day^{-1}	[49]
$K_{OPG-RANKL}$	2300	pM	[49]
$K_{RANK-RANKL}$	10	pM	[49]
$N^{RANK}_{Oc_p}$	4160	pM RANK / pM cell	[49]
β_{OPG,Ob_a}	$1.3 \cdot 10^5$	pM OPG / pM cell day^{-1}	[49]
$[OPG]_{max}$	131.4	pM	[49]
$[RANKL]_{max}$	5	pM	Optimized
$\beta_{RANKL,Ot}$	2836	pM RANKL / pM cell day^{-1}	[49]
β_{RANKL,Ob_p}	1395	pM RANKL / pM cell day^{-1}	Optimized
K^{RANKL}_{act,Oc_u}	2	pM	Optimized
K^{RANKL}_{act,Oc_p}	0.4	pM	Optimized
Upregulation of RANKL via damage			
ρ_{dam}	0.04	-	[49]
α_{dam}	1	-	[49]
δ_{dam}	0.3	-	Optimized
γ	1.4	-	Optimized
Competitive binding Wnt-Scl-LRP5/6			
$\tilde{D}^0_{Scl}$	5	day^{-1}	[49]
τ_{PMO}	10	years	Optimized
$\tilde{D}_{Scl-LRP5/6}$	50	day^{-1}	[49]
$K_{Wnt-LRP5/6}$	1079	pM	[49]

Continued on next page

Constant	Value	Units	Reference
$K_{Scl-LRP5/6}$	8.57	pM	[49]
$N_{OB_p}^{LRP5/6}$	5	pM LRP5/6 / pM cell	[49]
$\beta_{Scl,Ot}$	$5 \cdot 10^4$	pM Scl / pM cell day^{-1}	[49]
[Wnt]	170	pM	[49]
$[Scl]_{max}$	70	pM	[49]
$P_{Scl,d}$	0	pM day^{-1}	[49]
Co-regulation of RANKL via PTH and NO			
λ_s	0.457	-	[49]
λ_c	0.9	-	Optimized
K_{act}^{PTH}	3	pM	Optimized
K_{rep}^{PTH}	2.65	pM	Optimized
K_{rep}^{NO}	524	pM	[49]
$[NO]_{max}$	$2 \cdot 10^8$	pM	[49]
$\beta_{NO,Ot}$	1390	pM NO / pM cell day^{-1}	[49]
$\tilde{D}_{NO}$	$2.1 \cdot 10^{-3}$	day^{-1}	[49]
$P_{NO,d}$	0	pM day^{-1}	[49]
TGF-β related constants			
$\frac{\alpha_{TGF-\beta} k_{res}}{\tilde{D}_{TGF-\beta}}$	1	-	[49]
$K_{act}^{TGF-\beta}$	$5.633 \cdot 10^{-4}$	pM	[49]
$K_{rep}^{TGF-\beta}$	$1.754 \cdot 10^{-4}$	pM	[49]
Parameters of mechanical feedback			
α_{rep}	1	-	[49]
α_{act}	1	-	[49]
ρ_{rep}	0	-	[49]
ρ_{act}	0	-	[49]
δ_{rep}	1307	$\mu\varepsilon$	[49]
δ_{act}	619	$\mu\varepsilon$	
γ_{rep}	6	-	[49]
γ_{act}	11.5	-	[49]
Gut compartment			
D_1 (nominal)	25	mmol $\cdot$ day^{-1}	[1]
k_{1-f}	0.797	h^{-1}	[25]
k_{1-4}	0.406	h^{-1}	[25]
k_{pas}	0.1316	-	[25]
φ_{1-4}	0.0351	mmol	[25]
$\alpha_{1,1-4}$	0.55	mmol	[25]
$\delta_{1,1-4}$	0.2364	mmol	[25]
$\gamma_{1,1-4}$	1.7	-	[25]
$\rho_{6,2}$	0.0986	-	Optimized using [27]
$\alpha_{6,2}$	1.0	-	Optimized using [27]
$\delta_{6,2}$	300	pmol	Optimized using [27]
$\gamma_{6,2}$	3.0	-	Optimized using [27]

Continued on next page

Constant	Value	Units	Reference
D_3	45.16	$mmol \cdot day^{-1}$	[35]
k_{3-f}	0.801	h^{-1}	[35]
	Serum compartment		
V_{plasma}	3	L	[50]
k_{3-5}	1.762	h^{-1}	[35]
k_{5-3}	$7.467 \cdot 10^{-2}$	h^{-1}	[35]
$\rho_{6,4-u}$	0.85	-	Optimized
$\alpha_{6,4-u}$	7	-	Optimized
$\delta_{6,4-u}$	104.6	pmol	Optimized
$\gamma_{6,4-u}$	3.5	-	Optimized
$k_{4-u,filt}$	0.7086	-	Optimized
m_{4-u}	0.2185	L^{-1}	Optimized
n_{4-u}	0.1	mM	Optimized
$\rho_{7,4-u}$	0.998	-	Optimized
$\alpha_{7,4-u}$	1.002	-	[1]
$\delta_{7,4-u}$	10.2	pmol	Optimized
$\gamma_{7,4-u}$	1	-	Optimized
k_{4-13}	$7.086 \cdot 10^{-2}$	h^{-1}	[25]
k_{13-4}	0.1427	h^{-1}	[25]
k_{4-s}	0.0085	h^{-1}	[33]
k_{4-8}	0.1417	h^{-1}	[51]
k_{8-4}	$8.2 \cdot 10^{-2}$	h^{-1}	[51]
k_{5-14}	$7.467 \cdot 10^{-2}$	h^{-1}	[35]
k_{14-5}	0.159	h^{-1}	[35]
k_{5-9}	0.1417	h^{-1}	[1]
k_{9-5}	$2.6 \cdot 10^{-4}$	h^{-1}	Homeostasis
k_{5-u}	1.307	-	[35]
φ_{5-u}	1.046	mM	[35]
	Endocrine factors		
$k_{25-OH,D}$ (nominal)	1	h^{-1}	[1]
k_{6D}	0.1	h^{-1}	[28]
k_{7D}	10	h^{-1}	[52]
k_{7S}	102	pmol h^{-1}	Adjusted
k_{10D}	0.05	h^{-1}	[1]
k_{10S}	1.5	pmol h^{-1}	Homeostasis
$\rho_{7,10}$	0.97	-	Adjusted
$\alpha_{7,10}$	2.2	-	Adjusted
$\delta_{7,10}$	63.72	pmol	Adjusted
$\gamma_{7,10}$	2	-	[1]
$\rho_{5,10}$	0.18	-	Adjusted
$\alpha_{5,10}$	1.5	-	[1]
$\delta_{5,10}$	3.75	pmol	Adjusted
$\gamma_{5,10}$	12	-	Adjusted

Continued on next page

Constant	Value	Units	Reference
α_{11}	0.01	-	[1]
$b_{T6,4}$	0.01	-	Adjusted
$\delta_{T6,4}$	100	pM	Adjusted
$\gamma_{4,11}$	8	-	Adjusted
k_{12S}	0.0006	h^{-1}	Optimized
k_{12D}	0.0006	h^{-1}	Optimized
$\rho_{6,12}$	0.942	-	Adjusted
$\alpha_{6,12}$	5.5	-	Adjusted
$\delta_{6,12}$	153.3	pmol	Adjusted
$\gamma_{6,12}$	6.5	-	Adjusted
Composition of bone matrix and mineralisation model			
t_R	$2 \cdot 10^4$	days	[49]
k_{13-15}	3.435	h^{-1}	Optimized
v_m^{max}	0.442	-	[17, 49]
v_o	0.36	-	[53]
ρ_m	3.0	g/cm^3	[53]
ρ_o	1.4	g/cm^3	[53]
ρ_w	1	g/cm^3	[53]

Table 1: Values of the constants of the BCPM and homeostasis models.

References

[1] M.C. Peterson, M.M. Riggs. A physiologically based mathematical model of integrated calcium homeostasis and bone remodeling. Bone 2010;46(1):49–63. doi:10.1016/j.bone.2009.08.053.

[2] P. Pivonka, J. Zimak, D.W. Smith, et al. Model structure and control of bone remodeling: A theoretical study. Bone 2008;43(2):249–63. doi:10.1016/j.bone.2008.03.025.

[3] P. Pivonka, P. Buenzli, C.R. Dunstan. A Systems Approach to Understanding Bone Cell Interactions in Health and Disease. USA: InTech. ISBN 9789535107927; 2012, p. 169–204.

[4] M. Martin, V. Sansalone, Cooper D. M. L., M. R. Forwood, P. Pivonka. Mechanobiological osteocyte feedback drives mechanostat regulation of bone in a multiscale computational model. Biomech Model Mechanobiol 2019;18(5):1475–96. doi:10.1007/s10237-019-01158-w.

[5] T. Nakashima, M. Hayashi, T. Fukunaga, K. Kurata, M. Oh-hora, et al. Evidence for osteocyte regulation of bone homeostasis through rankl expression. Nat Med 2011;17(10):1231–4. doi:10.1038/nm.2452.

[6] J. Xiong, M. Piemontese, M. Onal, et al. Osteocytes, not osteoblasts or lining cells, are the main source of the rankl required for osteoclast formation in remodeling bone. PLOS ONE 2015;10(9):e0138189. doi:10.1371/journal.pone.0138189.

[7] J. Martínez-Reina, J.L. Calvo-Gallego, P. Pivonka. Combined effects of exercise and denosumab treatment on local failure in post-menopausal osteoporosis — insights from bone remodelling simulations accounting for mineralisation and damage. Front Bioeng Biotechnol 2021;9:635056. doi:10.3389/fbioe.2021.635056.

[8] A. R. Wijenayaka, M. Kogawa, H. P. Lim, L. F. Bonewald, D. M. Findlay, et al. Sclerostin stimulates osteocyte support of osteoclast activity by a RANKL-dependent pathway. PLOS ONE 6 2011;6(10):e25900. doi:https://doi.org/10.1371/journal.pone.0025900.

[9] H. M. Frost. Bone “mass” and the “mechanostat”: a proposal. Anat Rec 1987;219(1):1–9. doi:10.1002/ar.1092190104.

[10] P. Pivonka, P.R. Buenzli, S. Scheiner, et al. The influence of bone surface availability in bone remodelling — a mathematical model including coupled geometrical and biomechanical regulations of bone cells. Engineering Structures 2013;47:134–47.

[11] R. Ruiz-Lozano, J. L. Calvo-Gallego, P. Pivonka, M. McDonald, J. Martínez-Reina. An in-silico approach to elucidate the pathways leading to primary osteoporosis: age-related vs. postmenopausal. Biomech Model Mechan 2024;:1–17doi:10.1007/s10237-024-01846-2.

[12] J. Martínez-Reina, J. Ojeda, J. L. Calvo-Gallego, P. Pivonka, S. Martelli. Assessment of mechanical variables best describing bone remodelling responses based on their correlation with bone density. J Mech Behav Biomed Mater 2024;160:106773. doi:10.1016/j.jmbbm.2024.106773.

[13] A. Parfitt. Targeted and nontargeted bone remodeling: relationship to basic multicellular unit origination and progression. Bone 2002;30(1):5–7. doi:10.1016/s8756-3282(01)00642-1.

[14] J. Lemaitre, J. L. Chaboche. Mechanics of Solid Materials. Cambridge, UK: Cambridge University Press; 1990. doi:https://doi.org/10.1017/CBO9781139167970.

[15] P. Zioupos, J. Currey. Changes in the stiffness, strength, and toughness of human cortical bone with age. Bone 1998;22(1):57–66. doi:10.1016/s8756-3282(97)00228-7.

[16] C. Pattin, W. Caler, D. Carter. Cyclic mechanical property degradation during fatigue loading of cortical bone. J Biomech 1996;29(1):69–79. doi:10.1016/0021-9290(94)00156-1.

[17] J. Martínez-Reina, J.M. García-Aznar, J. Domínguez, M. Doblaré. On the role of bone damage in calcium homeostasis. J Theor Biol 2008;254(3):704–12. doi:10.1016/j.jtbi.2008.06.007.

[18] J. Martínez-Reina, J. García-Aznar, J. Domínguez, M. Doblaré. A bone remodelling model including the directional activity of BMUs. Biomech Model Mechanobiol 2009;8(2):111–27. doi:10.1007/s10237-008-0122-5.

[19] J. García-Aznar, T. Rueberg, M. Doblaré. A bone remodelling model coupling microdamage growth and repair by 3D BMU-activity. Biomech Model Mechanobiol 2005;4(2-3):147–67. doi:10.1007/s10237-005-0067-x.

[20] R. Juvinall. Engineering Considerations of Stress, Strain and Strength. New York, USA: McGraw-Hill. ISBN 978-0070331808; 1967,.

[21] J. Currey. Tensile yield in compact bone is determined by strain, postyield behaviour by mineral content. J Biomech 2004;37(4):549–56. doi:10.1016/j.jbiomech.2003.08.008.

[22] P. R. Buenzli, P. Pivonka, B. S. Gardiner, D. W. Smith. Modelling the anabolic response of bone using a cell population model. J Theor Biol 2012;307:42–52. doi:10.1016/j.jtbi.2012.04.019.

[23] M. K. Nguyen, I. Kurtz. Quantitative interrelationship between Gibbs-Donnan equilibrium, osmolality of body fluid compartments, and plasma water sodium concentration. J Appl Physiol 2006;100(4):1293–300. doi:10.1152/japplphysiol.01274.2005.

[24] J. E. Hall. Guyton and hall textbook of medical physiology. 13 ed.; London, England: W.B. Saunders; 2025. ISBN 978-1455770052.

[25] G. J. Strewler. Parathyroid Hormone and Calcium Homeostasis. Elsevier; 2003, p. 135–72. doi:10.1016/B978-012286551-0/50007-5.

[26] C.C. McCormick. Passive diffusion does not play a major role in the absorption of dietary calcium in normal adults. J Nutr 2002;132(11):3428–30. doi:10.1093/jn/132.11.3428.

[27] A. G. Need, P. D. O'Loughlin, H. A. Morris, P. S. Coates, M. Horowitz, et al. Vitamin D metabolites and calcium absorption in severe vitamin D deficiency. J Bone Miner Res 2008;23(11):1859–63. doi:10.1359/jbmr.080607.

[28] G. R. Bailie, C. A. Johnson. Comparative review of the pharmacokinetics of vitamin D analogues. Semin Dial 2002;15(5):352–7. doi:10.1046/j.1525-139x.2002.00086.x.

[29] A. Parfitt. Calcium homeostasis. In: G. R. Mundy, T. J. Martin, editors. Physiology and pharmacology of bone. Berlin: Springer-Verlag; 1993, p. 1–66.

[30] S. J. Silverberg, E. Shane, T. P. Jacobs, E. Siris, J. P. Bilezikian. A 10-year prospective study of primary hyperparathyroidism with or without parathyroid surgery. N Engl J Med 1999;341(17):1249–55. doi:10.1056/NEJM199910213411701.

[31] M. R. Rubin, J. P. Bilezikian, D. J. McMahon, T. Jacobs, E. Shane, et al. The natural history of primary hyperparathyroidism with or without parathyroid surgery after 15 years. J Clin Endocrinol Metab 2008;93(9):3462–70. doi:10.1210/jc.2007-1215.

[32] F. Bronner. Mechanisms of intestinal calcium absorption. J Cell Biochem 2003;88(2):387–893. doi:10.1002/jcb.10330.

[33] P. Evenepoel, H. S. Jørgensen, J Bover, A. Davenport, J. Bacchetta, et al. Recommended calcium intake in adults and children with chronic kidney disease – A European consensus statement. Nephrol Dial Transplant 2024;39(2):341–366. doi:https://doi.org/10.1093/ndt/gfad185.

[34] G. H. Kim. Renal mechanisms for hypercalciuria induced by metabolic acidosis. Am J Nephrol 2022;53(11-12):839–46. doi:10.1159/000528089.

[35] C. A. Wagner. The basics of phosphate metabolism. Nephrol Dial Transplant 2024;39(2):190–201. doi:10.1093/ndt/gfad188.

[36] K. D. Cashman. Calcium intake, calcium bioavailability and bone health. Br J Nutr 2002;87(S2):S169–77. doi:10.1079/BJNBJN/2002534.

[37] D. A. Goldstein. Clinical Methods: The History, Physical, and Laboratory Examinations. 3rd edition; chap. Serum Calcium. Boston: Butterworths. ISBN 0-409-90077-X; 1990,.

[38] C. Hindorf, G. Glatting, C. Chiesa, O. Lindén, G. Flux, et al. EANM Dosimetry Committee guidelines for bone marrow and whole-body dosimetry. Eur J Nucl Med Mol Imaging 2010;37(6):1238–50. doi:10.1007/s00259-010-1422-4.

[39] U. A. Gurkan, O. Akkus. An implantable magnetoelastic sensor system for wireless physiological sensing of viscosity. In: Proceeding of the ASME Summer Bioengineering Conference. Amer. Soc. Mechanical Engineers; 2007, p. 759–760. doi:https://doi.org/10.1115/SBC2007-176707.

[40] D. R. White, H. Q. Woodard, S. M. Hammond. Average soft-tissue and bone models for use in radiation dosimetry. Br J Radiol 1987;60(717):907–13. doi:10.1259/0007-1285-60-717-907.

[41] S-C. A. Yeh, J. Hou, J. W. Wu, S. Yu, Y. Zhang, et al. Quantification of bone marrow interstitial pH and calcium concentration by intravital ratiometric imaging. Nat Commun 2022;13(1):393. doi:10.1038/s41467-022-27973-x.

[42] H. A. Qadeer, K. Bashir. Physiology, Phosphate. In: StatPearls [Internet]. Treasure Island (FL): StatPearls Publishing; 2025,.

[43] B. L. Langdahl, S. Ferrari, D. W. Dempster. Bone modeling and remodeling: potential as therapeutic targets for the treatment of osteoporosis. Ther Adv Musculoskelet Dis 2016;8(6):225–35. doi:10.1177/1759720X16670154.

[44] L. Cardoso, S. P. Fritton, G. Gailani, M. Benalla, S. C. Cowin. Advances in assessment of bone porosity, permeability and interstitial fluid flow . J Biomech 2013;46(2):253–65. doi:10.1016/j.jbiomech.2012.10.025.

[45] D. Ulrich, B. van Rietbergen, A. Laib, P. Rüegsegger. The ability of three-dimensional structural indices to reflect mechanical aspects of trabecular bone. Bone 1999;25(2):55–60. doi:10.1016/s8756-3282(99)00098-8.

[46] W. S. S. Jee. Histology: Cell and Tissue Biology; chap. The Skeletal Tissues. 5th ed.; Amsterdam: Elsevier; 1983,.

[47] S. K. Bhadada, J. Ghosh, R. Pal, S. Mukherjee. Phosphate: An underrated component of primary hyperparathyroidism. Best Pract Res Clin Endocrinol Metab 2024;38(2):101837. doi:10.1016/j.beem.2023.101837.

[48] M. Rix, H. Andreassen, P. Eskildsen, B. Langdahl, K. Olgaard. Bone mineral density and biochemical markers of bone turnover in patients with predialysis chronic renal failure. Kidney Int 1999;56(3):1084–93. doi:10.1046/j.1523-1755.1999.00617.x.

[49] R. Ruiz-Lozano, J. L. Calvo-Gallego, P. Pivonka, J. Martínez-Reina. Optimisation of romosozumab plus denosumab sequential treatments against postmenopausal osteoporosis. Insights from in silico simulations. Biomech Model Mechanobiol 2025;doi:10.1007/s10237-024-01900-z.

[50] R. G. Hahn, J. H. Zdolsek. Estimating the plasma volume by infusing albumin: a retrospective feasibility study. Intensive Care Med Exp 2025;13(1):35. doi:10.1186/s40635-025-00743-x.

[51] P. Felig, L. A. Frohman, editors. Endocrinology & Metabolism. 2 ed.; McGraw-Hill; 1987. ISBN 978-0070203907.

[52] C. Bieglmayer, G. Prager, B. Niederle. Kinetic analyses of parathyroid hormone clearance as measured by three rapid immunoassays during parathyroidectomy. Clin Chem 2002;48(10):1731–8. doi:10.1093/clinchem/48.10.1731.

[53] R. Martin. Porosity and specific surface of bone. Critical Reviews in Biomedical Engineering 1984;10(3):179–222.
